# High-density lipoprotein regulates angiogenesis by long non-coding RNA HDRACA

**DOI:** 10.1038/s41392-023-01558-6

**Published:** 2023-08-14

**Authors:** Zhi-Wei Mo, Yue-Ming Peng, Yi-Xin Zhang, Yan Li, Bi-Ang Kang, Ya-Ting Chen, Le Li, Mary G. Sorci-Thomas, Yi-Jun Lin, Yang Cao, Si Chen, Ze-Long Liu, Jian-Jun Gao, Zhan-Peng Huang, Jia-Guo Zhou, Mian Wang, Guang-Qi Chang, Meng-Jie Deng, Yu-Jia Liu, Zhen-Sheng Ma, Zuo-Jun Hu, Yu-Gang Dong, Zhi-Jun Ou, Jing-Song Ou

**Affiliations:** 1 Division of Cardiac Surgery, Cardiovascular Diseases Institute, The First Affiliated Hospital, Sun Yat-sen University, Guangzhou, China; 2 National-Guangdong Joint Engineering Laboratory for Diagnosis and Treatment of Vascular Diseases, NHC Key Laboratory of Assisted Circulation (Sun Yat-sen University), Guangdong Provincial Engineering and Technology Center for Diagnosis and Treatment of Vascular Diseases, Guangzhou, China; 3 Division of Vascular Surgery, The First Affiliated Hospital, Sun Yat-sen University, Guangzhou, China; 4 Division of Hypertension and Vascular Diseases, Department of Cardiology, Heart Center, The First Affiliated Hospital, Sun Yat-sen University, Guangzhou, China; 5 Medical College of Wisconsin, Milwaukee, WI USA; 6 Center for Translational Medicine, Institute of Precision Medicine, The First Affiliated Hospital, Sun Yat-sen University, Guangzhou, China; 7 Department of Cardiology, Heart Center, The First Affiliated Hospital, Sun Yat-sen University, Guangzhou, China; 8 Department of Pharmacology, Cardiac and Cerebral Vascular Research Center, Zhongshan School of Medicine of Sun Yat-sen University, Guangzhou, China; 9 Guangdong Provincial Key Laboratory of Brain Function and Disease, Zhongshan School of Medicine, Sun Yat-sen University, Guangzhou, 510080 P.R. China

**Keywords:** Cardiology, Non-coding RNAs

## Abstract

Normal high-density lipoprotein (nHDL) can induce angiogenesis in healthy individuals. However, HDL from patients with coronary artery disease undergoes various modifications, becomes dysfunctional (dHDL), and loses its ability to promote angiogenesis. Here, we identified a long non-coding RNA, HDRACA, that is involved in the regulation of angiogenesis by HDL. In this study, we showed that nHDL downregulates the expression of HDRACA in endothelial cells by activating WW domain-containing E3 ubiquitin protein ligase 2, which catalyzes the ubiquitination and subsequent degradation of its transcription factor, Kruppel-like factor 5, via sphingosine 1-phosphate (S1P) receptor 1. In contrast, dHDL with lower levels of S1P than nHDL were much less effective in decreasing the expression of HDRACA. HDRACA was able to bind to Ras-interacting protein 1 (RAIN) to hinder the interaction between RAIN and vigilin, which led to an increase in the binding between the vigilin protein and proliferating cell nuclear antigen (PCNA) mRNA, resulting in a decrease in the expression of PCNA and inhibition of angiogenesis. The expression of human HDRACA in a hindlimb ischemia mouse model inhibited the recovery of angiogenesis. Taken together, these findings suggest that HDRACA is involved in the HDL regulation of angiogenesis, which nHDL inhibits the expression of HDRACA to induce angiogenesis, and that dHDL is much less effective in inhibiting HDRACA expression, which provides an explanation for the decreased ability of dHDL to stimulate angiogenesis.

## Introduction

Coronary artery disease (CAD), also known as ischemic heart disease, is the leading cause of death worldwide. In the past two decades, pre-clinical research has stimulated clinical trials aimed at stimulating coronary vascular growth as a treatment for CAD.^1^ Unfortunately, these trials failed.^2^ Therefore, no effective methods are currently available to promote coronary vascular growth in patients with CAD. The reasons for failure are likely many, but one consensus is that the myriad risk factors contributing to vascular disease and endothelial dysfunction in patients with CAD present a far more complicated task than stimulating blood vessel growth in pre-clinical models without vascular disease. A notable and perhaps underestimated risk frequently involves modification of high-density lipoproteins (HDL).

HDL is a mixture of multiple proteins and lipids, including apolipoprotein A-I (ApoA I), apolipoprotein A-II (ApoII), apolipoprotein M (ApoM), and sphingosine 1-phosphate (S1P).^3,4^ Normal HDL (nHDL) can protect vascular function through reverse cholesterol transport, anti-oxidation, anti-inflammation, and endothelial protection.^5,6^ However, clinical trials have demonstrated that elevated plasma HDL levels, using cholesteryl ester transfer protein inhibitors or niacin, fail to reduce cardiovascular events.^7,8^ The components of HDL are altered or modified in CAD, and HDL is no longer protective or even damages the vessels.^5,6,9^ This type of HDL is called dysfunctional HDL (dHDL). We and others have previously reported that nHDL from healthy individuals can induce angiogenesis.^5,10^ However, we and others have recently found that HDL from patients with CAD is dysfunctional and less effective in inducing angiogenesis.^5,9^ Although we and others have reported that several important signaling pathways are involved in the regulation of angiogenesis by HDL,^5,10,11^ the mechanisms by which nHDL promotes angiogenesis and dHDL is less effective are not yet fully understood.

Non-coding RNAs (ncRNAs) are transcripts without protein-coding potential. ncRNAs have been recognized as high-quality biomarkers for risk stratification, diagnosis, and prognosis of multiple cardiovascular diseases.^12^ Recent reports indicate that HDL can serve as an important vector of ncRNAs in the plasma, and it can deliver ncRNAs to target cells, inducing a series of cellular actions that are important in angiogenesis.^5,13,14^ Additionally, HDL can regulate ncRNA expression to affect angiogenesis.^5,15^ MicroRNA (miRNA) is one of the important ncRNAs that regulates angiogenesis.^16^ Our previous study revealed that nHDL suppresses miR-24-3p expression in endothelial cells via scavenger receptor class B type 1 (SRB1), whereas dHDL delivers miR-24-3p into endothelial cells via SRB1. nHDL and dHDL differentially regulate miR-24-3p expression to affect angiogenesis via vinculin.^5^ However, miR-24-3p inhibition doesn’t completely restored dHDL-induced angiogenesis,^5^ suggesting that other factors may affect the regulation of angiogenesis by HDL. Although HDL can regulate miRNAs that affect angiogenesis, little is known about HDL and other ncRNAs. Long non-coding RNAs (lncRNAs) are arbitrarily defined as a class of ncRNAs comprising more than 200 nucleotides.^17^ Some previous studies have indicated the association between lncRNAs and vascular biology. For example, MALAT1 regulates endothelial function and vessel growth.^18^ SMRIL drives vascular smooth muscle cell cycle progression.^19^ Compared with miRNAs, lncRNA has a significantly larger quantity. To date, the recorded amount of human lncRNAs has surpassed 100,000, which is far below its actual quantity due to limited analysis.^20^ Moreover, the functions of lncRNA are more complex. Their functions are classified into cis and trans based on their proximity to their transcriptional sites, which are also related to their subcellular localization.^17,21^ Their multifaceted structures contribute to their multiple functions.^22^ In general, lncRNAs participate in transcriptional and post-transcriptional regulation, cell organellar and structural organisation and genome integrity by interacting with other factors including other ncRNAs.^23^ LncRNAs can also regulate miRNA functions by sponging or generating miRNAs.^24,25^ Therefore, studying HDL-regulated lncRNAs is of great significance in further comprehending the mechanisms underlying HDL-mediated angiogenesis. In a previous study, we identified lncRNAs that are differentially expressed in endothelial cells after treatment with nHDL or dHDL.^26^ However, the biological functions of the majority remain unclear.

In the present study, by analyzing our previous lncRNA-seq results, we identified a lncRNA that is differentially expressed in endothelial cells stimulated with nHDL or dHDL and is involved in HDL regulation of angiogenesis in CAD. We termed this lncRNA high-density lipoprotein-regulated angiogenesis in coronary artery disease (HDRACA). Our results showed that nHDL-bound S1P interacted with S1P receptor 1 (S1P1), leading to the activation of WW domain-containing E3 ubiquitin protein ligase 2 (WWP2), which induced the ubiquitination and degradation of Kruppel-like factor 5 (KLF5). Consequently, HDRACA expression was downregulated because of the reduced activation of its promoter by KLF5. However, dHDL was less effective because of its lower S1P content. We also found that HDRACA bound to Ras-interacting protein 1 (RAIN) and hindered its interaction with vigilin, which increased the interaction between vigilin and proliferating cell nuclear antigen (PCNA) mRNA, ultimately inhibiting angiogenesis. Our findings suggest that HDRACA can serve as a novel therapeutic target for angiogenesis in patients with CAD.

## Results

### nHDL and dHDL cause different expression of HDRACA in endothelial cells

**Fig. 1:**
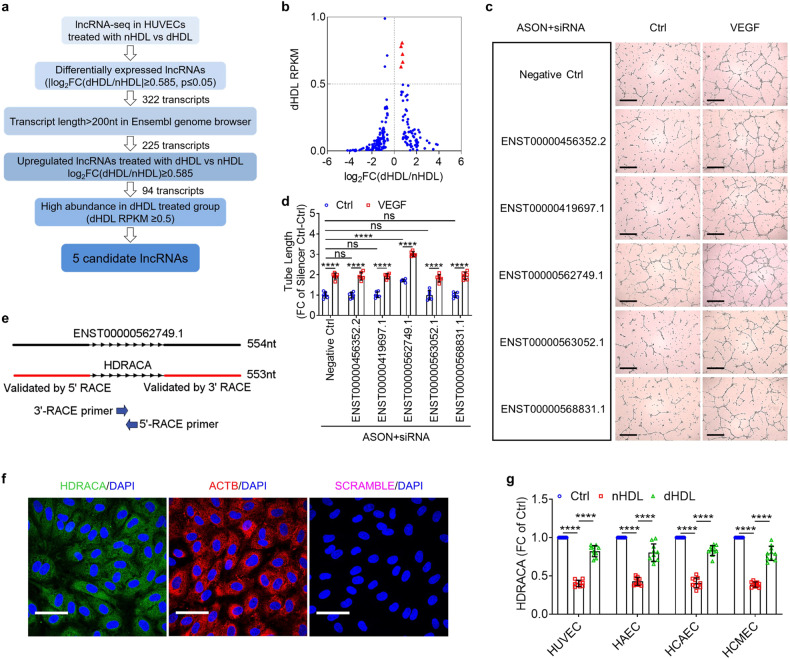
nHDL and dHDL cause different expression of HDRACA in endothelial cells. **a** Selection strategy of lncRNA in HUVECs treated with nHDL or dHDL. **b** Scatter plot of differentially expressed lncRNAs in HUVECs induced by nHDL and dHDL (Transcripts length > 200 nt in Ensembl genome browser database). Red symbols represent the 5 candidate lncRNAs. **c**, **d** Tube formation assays showed the effects of respectively knocking down 5 candidate lncRNAs using specific ASONs (66 nM) and siRNAs (33 nM) on angiogenesis. The representative images (**c**) and quantification (**d**) are shown. Scale bars, 500 μm. **e** RACE assays measured the full length of ENST00000562749.1 termed HDRACA. **f** FISH assays showed the subcelluar location of HDRACA in HUVECs. Representative images of HDRACA (green), ACTB (red) and Scramble-ISH (magenta) are shown. ACTB probes are shown as a positive control, and Scramble-ISH probes are shown as a negative control. The nuclei were stained with DAPI (blue). Scale bars, 50 μm. **g** RT-qPCR confirmed the expression of HDRACA in multiple kinds of endothelial cells treated with nHDL or dHDL. Data are presented as the mean ± SD. For (**c**) and (**d**), *n* = 6. For (**g**), *n* = 10. ****p < 0.0001; ns not significant

To identify lncRNAs involved in the nHDL and dHDL regulation of angiogenesis, we analyzed previous lncRNA sequencing results to select lncRNAs for their regulatory effects on angiogenesis.^26^ We preferred transcripts longer than 200nt in the Ensembl genome browser database to obtain preliminary information on the transcripts. We searched for transcripts with higher expression levels in the dHDL than in the nHDL group (*P* value ≤ 0.05, Fold Change ≥ 1.5). We focused on transcripts with relatively high abundance in the dHDL group (RPKM ≥ 0.5).^27^ Five candidate transcripts were identified (Fig. 1a, b). These five transcripts were knocked down using specific small interfering RNAs (siRNAs) and antisense oligonucleotides (ASONs) (Supplementary Fig. S1a) to evaluate their effects on angiogenesis. Knockdown of ENST00000562749.1 promoted both tube formation and proliferation of human umbilical vein endothelial cells (HUVECs), whereas knocking down the other four candidate transcripts had no effect (Fig. 1c, d; Supplementary Fig. S1b, d). Furthermore, knockdown of any of these five transcripts had no effect on endothelial cell migration (Supplementary Fig. S1c, e). Therefore, ENST00000562749.1 was chosen for further study.

In the Ensembl genome browser, ENST00000562749.1 is encoded by MRFAP1P1 gene that is located on the forward strand of Chromosome X. To determine the exact sequences of ENST00000562749.1, we performed 5ʹ and 3ʹ rapid amplification of complementary DNA ends (RACE) assays and found a 553-nt transcript (Fig. 1e; Supplementary Fig. S2a). We termed it HDRACA. Data from the coding potential assessment tool (CPAT) showed that HDRACA possessed very low coding potentials (Supplementary Fig. S2b). According to ORFfinder, HDRACA does not possess an open reading frame (ORF) longer than 300 nt (Supplementary Fig. S2c). Furthermore, we overexpressed these ORFs in HEK-293T cells and confirmed that none of the ORFs encoded peptides (Supplementary Fig. S2d). A fluorescence in situ hybridization (FISH) assay demonstrated that HDRACA was primarily localized in the cytoplasm of HUVECs (Fig. 1f), as verified by nuclear and cytoplasmic fractionation (Supplementary Fig. S2e). Real-time quantitative PCR (RT-qPCR) results indicated no significant changes in HDRACA levels at various time points during tube formation (Supplementary Fig. S2f). To examine the distribution of HDRACA in different types of vascular cells, droplet digital PCR (ddPCR) was performed to quantify the HDRACA levels in HUVECs, human aortic smooth muscle cells (HASMCs), human aortic fibroblasts (FBs), and THP-1 macrophages. We verified that HDRACA was expressed in various human blood vessel cells, but its expression was the highest in endothelial cells (Supplementary Fig. S2g). RT-qPCR verified the expression of HDRACA in endothelial cells stimulated with nHDL and dHDL. We found that nHDL reduced HDRACA expression in various types of endothelial cells, whereas dHDL was less effective than nHDL at downregulating HDRACA (Fig. 1g). We further verified this result using FISH assays (Supplementary Fig. S2h). These data suggest that HDRACA is a 553-nt lncRNA primarily localized in the cytoplasm of HUVECs, and nHDL can downregulate HDRACA expression levels in HUVECs, whereas dHDL is less effective than nHDL, which is consistent with the lncRNA-seq results.

### HDL-bound S1P regulate the transcription of HDRACA in the endothelial cells by affecting the ubiquitination of KLF5

**Fig. 2:**
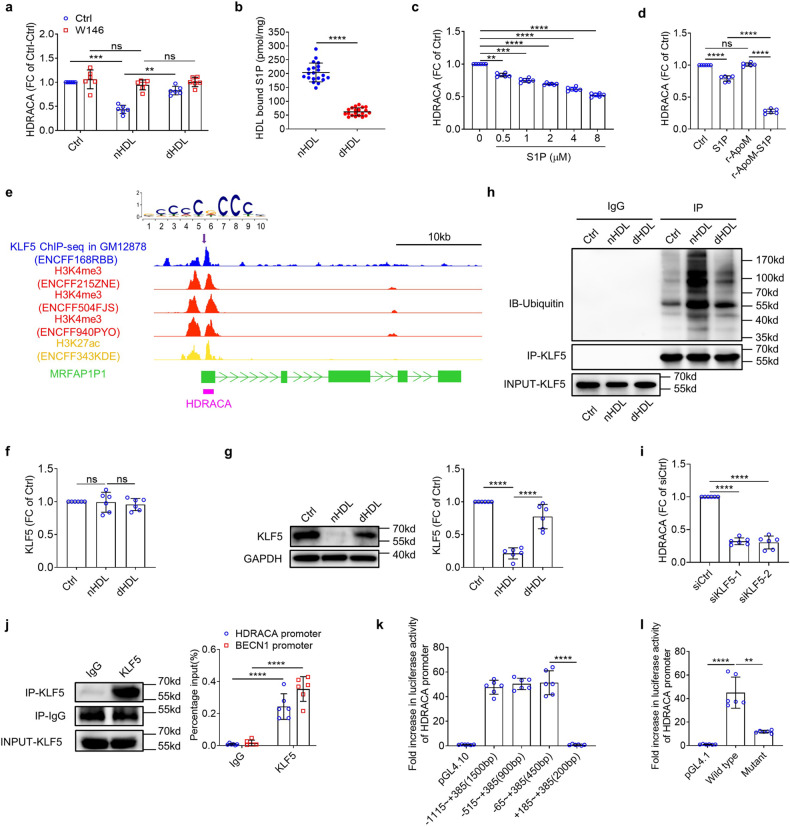
HDL-bound S1P regulates the transcription of HDRACA in the endothelial cells by affecting the ubiquitination of KLF5. **a** RT-qPCR showed the effect of W146 on HDRACA levels in HUVECs treated with nHDL or dHDL. **b** The S1P content in nHDL (*n* = 20) and dHDL (*n* = 20). **c** The levels of HDRACA in HUVECs treated with increasing concentrations of S1P (from 0.5 μM to 8 μM) were assayed by RT-qPCR. **d** The levels of HDRACA in HUVECs treated with S1P (1 μM), r-ApoM (1 μM), and r-ApoM-S1P (1 μM) were assayed by RT-qPCR. **e** The middle tracks display the online dataset of KLF5 ChIP-seq in GM12878 (blue; GSE127670), H3K4me3 ChIP-seq in HUVECs (red; GSM945181, GSM733673, GSE96250) and H3K27ac ChIP-seq in HUVECs (yellow; GSM733691). The purple arrow indicates the putative KLF5 binding site in HDRACA promoter based on the conserved KLF5 binding motif (shown on the top) and peaks. **f** The mRNA levels of KLF5 in HUVECs treated with nHDL or dHDL were assayed by RT-qPCR. **g** Immunoblot of KLF5 in HUVECs treated with nHDL or dHDL. The representative plots (left) and quantification (right) are shown. **h** KLF5 ubiquitination in HUVECs treated with nHDL or dHDL was assayed by immunoprecipitation (IP) and immunoblotting (IB). MG132 was added to inhibit KLF5 degradation. **i** RT-qPCR confirmed the mRNA levels of HDRACA in HUVECs transfected with negative control siRNA or KLF5-siRNAs. **j** ChIP-RT-qPCR analysis (right) for KLF5 binding to the HDRACA promoter in HUVECs. Normalized data are shown as percentages of the input controls. KLF5 binding to BECN1 promoter served as a positive control. The whole-cell lysate (INPUT) and immunoprecipitated proteins (IP) of each immunoprecipitation were analyzed by immunoblotting (left) for KLF5 or IgG. **k** Luciferase reporter assays for HUVECs with endogenous KLF5 expression transfected with pGL4.1 reporter plasmids containing deletion HDRACA promoter constructs. HUVECs transfected with a blank pGL4.1 plasmid served as a negative control. **l** Luciferase reporter assays for HUVECs with endogenous KLF5 expression transfected with pGL4.1 reporter plasmids containing wild type or mutated HDRACA promoters. HUVECs transfected with a blank pGL4.1 plasmid served as a negative control. Data are presented as the mean ± SD. For all the experiments, *n* = 6. **p < 0.01; ***p < 0.001; ****p < 0.0001; ns not significant

The differential regulation of HDRACA by nHDL and dHDL prompted us to explore its potential mechanism. As HDL usually regulates endothelial cell activities by interacting with cellular receptors, we designed siRNAs to knock down the five most common HDL receptors on endothelial cells (Supplementary Fig. S15a–e and S15l–p). Knockdown of ATP-binding cassette transporter A1 (ABCA1), ATP-binding cassette transporter G1 (ABCG1), SRB1, and S1P receptor 3 (S1P3) did not affect the regulatory effects of nHDL and dHDL on HDRACA (Supplementary Fig. S3a–d). However, when S1P1 was knocked down, both nHDL and dHDL failed to down regulate HDRACA expression (Supplementary Fig. S3e). W146, an S1P1 inhibitor, blocked the ability of nHDL and dHDL to decrease HDRACA levels (Fig. 2a).

S1P is an endogenous agonist of S1P1, and S1P is primarily carried by HDL particles in the plasma.^28^ Both S1P and HDL plasma levels were lower in patients with CAD than in healthy individuals (Supplementary Fig. S3f, g). However, when we extracted HDL from the plasma to measure its S1P content, dHDL from patients with CAD contained significantly lower S1P content than did nHDL from healthy individuals (Fig. 2b), which is consistent with a previous report.^29^ Plasma HDRACA levels were not significantly different between CAD patients and healthy individuals (Supplementary Fig. S3h). RT-qPCR results showed that S1P decreased HDRACA expression in HUVECs in a dose-dependent manner (Fig. 2c). Most S1P is bound to ApoM in HDL.^30^ ApoM levels were not significantly different between nHDL and dHDL (Supplementary Fig. S3i). Recombinant human ApoM (r-ApoM)-bound S1P (r-ApoM-S1P) showed a stronger inhibitory effect than free S1P on HDRACA in HUVECs (Fig. 2d). We administered nHDL and dHDL along with exogenous S1P. The results suggested that both nHDL and dHDL could efficiently take up S1P and S1P-loading could rescue the inhibitory effect of dHDL on HDRACA (Supplementary Fig. S3j, k). Additionally, we examined whether nHDL or dHDL could carry and deliver HDRACA into endothelial cells. We extracted RNA from nHDL and dHDL for lncRNA microarray analysis. The results showed that nHDL and dHDL carried numerous lncRNAs, including HDRACA (Supplementary Fig. S3l). Further, RT-qPCR indicated that there was no significant difference in the HDRACA content between nHDL and dHDL (Supplementary Fig. S3m). We used W146 to block the inhibitory effect of HDL-bound S1P on HDRACA expression in HUVECs, followed by treatment with α-amanitin, a specific inhibitor of RNA polymerases II and III, to inhibit endogenous RNA transcription. Neither nHDL nor dHDL affected HDRACA levels in HUVECs (Supplementary Fig. S3n). Collectively, nHDL and dHDL affected the levels of HDRACA by activating S1P1 in endothelial cells via S1P, rather than delivering HDRACA into endothelial cells.

According to JASPAR, a database of transcription factor binding profiles, KLF5, a transcription factor closely related to angiogenesis,^31^ was predicted to bind to the promoter of HDRACA with a canonical binding motif (Fig. 2e). An online chromatin immunoprecipitation (ChIP) sequencing dataset (GSE127670) revealed the peaks of KLF5 at the HDRACA promoter in GM12878 cells (Fig. 2e). Moreover, several online histone ChIP-seq datasets (GSM945181, GSM733673, GSE96250, and GSM733691) showed that H3K4me3 and H3K27ac peaked in HUVECs in the same region as the two KLF5 peaks in the HDRACA promoter in GM12878 cells (Fig. 2e). Therefore, we evaluated whether nHDL and dHDL differentially regulated KLF5 activity to affect HDRACA levels. Although neither nHDL nor dHDL affected the levels of KLF5 mRNA in HUVECs (Fig. 2f), nHDL effectively downregulated KLF5 protein levels, whereas dHDL had less of an effect (Fig. 2g). In addition, r-ApoM-S1P did not affect KLF5 mRNA levels in HUVECs but significantly downregulated KLF5 protein levels (Supplementary Fig. S4a, b). Furthermore, S1P1 knockdown blocked the ability of nHDL, dHDL, and r-ApoM-S1P to downregulate KLF5 protein levels without affecting KLF5 mRNA levels (Supplementary Fig. S4c–f). Previous studies have reported that KLF5 is an unstable protein that can be ubiquitinated by multiple ubiquitin-protein ligases and degraded by the ubiquitin-proteasome system.^32^ Treatment of HUVECs with the proteasome inhibitor MG132 increased KLF5 protein levels and attenuated the decrease in KLF5 protein levels after nHDL stimulation (Supplementary Fig. S4g). As shown in Fig. 2h, nHDL increased the ubiquitination of KLF5 in HUVECs, while dHDL had much less effect, suggesting that nHDL and dHDL differentially regulate the ubiquitination of KLF5 to affect its degradation. r-ApoM-S1P promoted KLF5 ubiquitination (Supplementary Fig. S5a).

WWP2, an E3 ubiquitin ligase, can facilitate multiple types of ubiquitination of its substrates, leading to proteolysis or changes in activity, depending on the type of E2 ubiquitin-conjugating enzyme it associates with.^33–35^ S1P1 is constitutively associated with WWP2, and WWP2 is able to catalyze KLF5 ubiquitination.^36,37^ A recent study also indicated the important role of WWP2 in protecting vessels from oxidative stress-associated endothelial injury.^38^ Therefore, we investigated whether S1P activates WWP2 to induce KLF5 ubiquitination in HUVECs. WWP2 knockdown effectively attenuated r-ApoM-S1P-induced ubiquitination and downregulation of KLF5 (Supplementary Fig. S5b, c; Supplementary Fig. S15f, q). The WW2-WW3 linker (2,3-linker) is critical for the autoinhibition of WWP2 enzymatic activity.^39^ However, phosphorylation at two tyrosine sites, Tyr369 and Tyr392, in the 2,3-linker region can disrupt the inhibitory effects of 2,3-linker on WWP2 catalysis.^39^ S1P-S1P1 signaling promotes the phosphorylation of protein tyrosine sites by activating receptor protein tyrosine kinases or non-receptor tyrosine kinase.^40,41^ To investigate whether S1P-S1P1 signaling activated WWP2 by promoting the phosphorylation of its tyrosine sites, we transduced HUVECs with lentiviruses encoding FLAG-tagged wild-type WWP2 or mutant WWP2, in which Tyr369 or Tyr392 was replaced with phenylalanine (Y369F or Y392F) (Supplementary Fig. S5d). Supplementary Fig. S5e shows that r-ApoM-S1P promoted the tyrosine phosphorylation of WWP2, which was attenuated by the Y369F or Y392F mutation. The Y369F and Y392F mutations in WWP2 decreased r-ApoM-S1P induced KLF5 ubiquitination (Supplementary Fig. S5f). WWP2 interacts with the PY2 motif of KLF5 to promote KLF5 ubiquitination.^37^ PY2 has been demonstrated to directly degraded KLF5.^42^ Transducing HUVECs with lentiviruses encoding KLF5 with a PY2 deletion (KLF5-ΔPY2) effectively upregulated KLF5 levels, even after nHDL treatment (Supplementary Fig. S5g; Supplementary Fig. S15v). Knocking down KLF5 using siRNAs downregulated HDRACA levels in HUVECs, while overexpressing KLF5-ΔPY2 significantly upregulated HDRACA levels and antagonized the inhibition of nHDL on HDRACA (Fig. 2i; Supplementary Fig. S5h, i; Supplementary Fig. S15g, r).

Next, we searched for the binding sites for KLF5 based on the conserved binding motif and online ChIP-seq datasets. We found a canonical-GCCCCGCCCC-binding sequence in the common region of KLF5 and histone peaks in the promoter region of HDRACA [+73 to +82] (Fig. 2e). To validate the association between KLF5 and the predicted transcription factor-binding site, we performed ChIP-RT-qPCR and confirmed the interaction between KLF5 and the HDRACA promoter region (Fig. 2j). We used the BECN1 promoter as a positive control, which has been previously shown to bind to KLF5.^43^ Additionally, KLF5 knockdown attenuated KLF5 enrichment in the HDRACA promoter (Supplementary Fig. S5j). We further transfected HUVECs with a series of luciferase reporter constructs carrying sequential deletions of the 5ʹ-flanking regions of HDRACA. We observed enhanced luciferase activity with sequence deletion from −1115 to −65 base pairs upstream of the HDRACA transcription start site (Fig. 2k). Additionally, a nucleotide mutation at the KLF5 binding site [+73 to +82] abrogated luciferase activity following transfection of the pGL4.1 reporter plasmid (Fig. 2l).

Taken together, these data suggest that nHDL-bound S1P interacts with S1P1 to activate WWP2, promotes the ubiquitination-mediated degradation of KLF5, and inhibits the transcription of HDRACA, whereas dHDL does not significantly affect KLF5 ubiquitination and degradation because of its lower S1P content, resulting in less effective regulation of HDRACA transcription.

### nHDL and dHDL differently regulate HDRACA expression to affect endothelial cell proliferation and tube formation

**Fig. 3:**
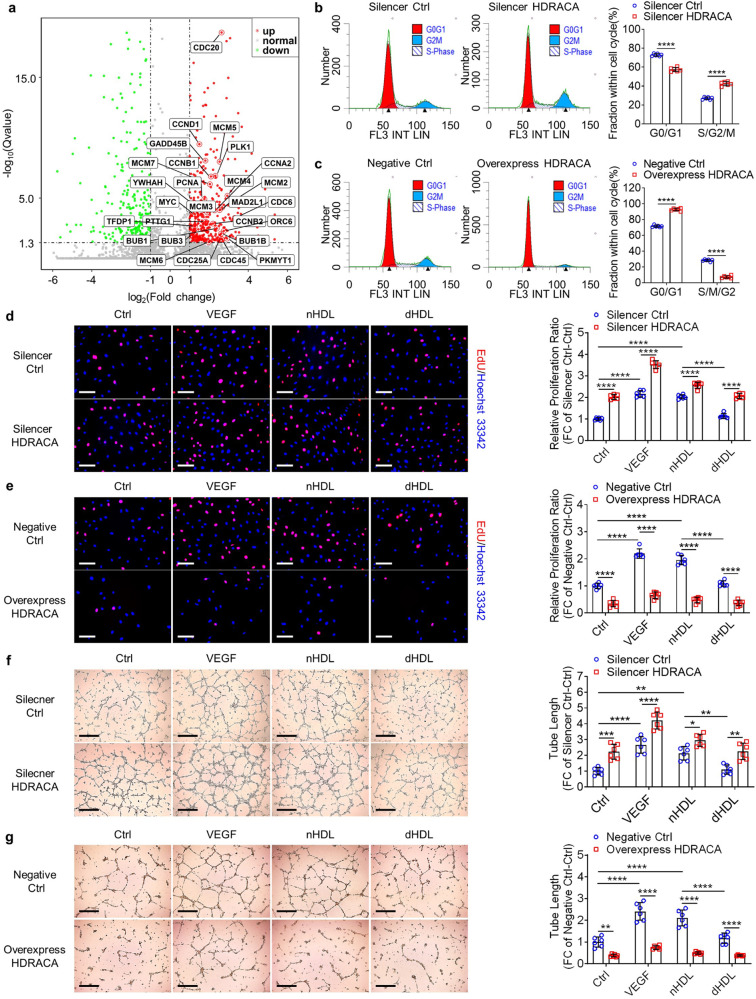
nHDL and dHDL differently regulate HDRACA expression to affect endothelial cell proliferation and tube formation. **a** Volcano plot shows differentially expressed genes in HUVECs after treatment with lncRNA Smart Silencer HDRACA (Silencer HDRACA) compared with lncRNA smart silencer negative control (Silencer Ctrl), *n* = 3. **b**, **c** Flow cytometric analysis of HUVECs showing the cell cycle at 30 min after addition of PI/RNAse and 48 h after lncRNA Smart Silencer transfection (**b**) or 72 h after lentivirus vector transfection (**c**). The representative plots (left and middle) and quantification (right) are shown. **d**, **e** The representative images (left) and quantification (right) of EdU incorporation assay in HUVECs treated with VEGF or nHDL or dHDL after silencing (**d**) or overexpressing (**e**) HDRACA. The proliferative HUVECs were labeled with EdU (red) and the nuclei were stained with Hoechst 33342 (blue). Scale bars, 100 μm. **f**, **g** The representative images (left) and quantification (right) of tube formation assay in HUVECs treated with VEGF or nHDL or dHDL after silencing (**f**) or overexpressing (**g**) HDRACA. Scale bars, 500 μm. Data are presented as the mean ± SD. For (**b**–**g**), *n* = 6. *p < 0.05; **p < 0.01; ***p < 0.001; ****p < 0.0001

To further address the role of HDRACA in angiogenesis, we silenced HDRACA using a specific mixture of siRNAs and ASONs (lncRNA Smart Silencer), and overexpressed HDRACA by transducing HUVECs with lentiviral vectors. The lncRNA Smart Silencer efficiently silenced HDRACA (downregulated more than 80%), and the lentivirus carrying the full-length sequence of HDRACA significantly increased HDRACA levels in HUVECs (Supplementary Fig. S6a, b). After silencing HDRACA in HUVECs, we performed mRNA sequencing and subjected the differentially expressed genes to GO and KEGG pathway analyses (Fig. 3a; Supplementary Fig. S6c–e). These results indicate that the cell cycle was the most regulated pathway upon HDRACA silencing in HUVECs (Supplementary Fig. S6d). Flow cytometry analysis revealed that silencing HDRACA increased the fraction of HUVECs in the S/G2/M phase (Fig. 3b). In contrast, transfection of HUVECs with a lentivirus encoding HDRACA decreased the proportion of HUVECs in S/G2/M (Fig. 3c). We evaluated the role of HDRACA in HUVECs apoptosis using Annexin V-FITC/PI staining and TdT-mediated dUTP nick-end labelling (TUNEL) assays. Silencing or overexpression of HDRACA did not change the proportion of apoptotic cells (Supplementary Fig. S6f, g). These data suggest that HDRACA negatively regulates the endothelial cell cycle, but does not affect endothelial cell apoptosis.

We performed Cell Counting Kit 8 assays and 5-Ethynyl-2ʹ-deoxyuridine (EDU) incorporation assays to verify the potential effect of HDRACA on endothelial cell proliferation. nHDL and r-ApoM-S1P promoted HUVECs proliferation; however, dHDL were less effective than nHDL (Fig. 3d, e; Supplementary Fig. S7a–d). Silencing of HDRACA promoted HUVECs proliferation (Fig. 3d; Supplementary Fig. S7a). Overexpression of HDRACA not only significantly inhibited HUVECs proliferation but also attenuated the promoting effect of nHDL and r-ApoM-S1P (Fig. 3e; Supplementary Fig. S7b–d). These data support the notion of an important role in regulating endothelial cell proliferation. nHDL promoted HUVEC migration; however, dHDL were less effective (Supplementary Fig. S7e, f). Unlike proliferation, the silencing or overexpression of HDRACA did not affect HUVEC migration (Supplementary Fig. S7e, f). Furthermore, we determined the effect of HDRACA on tube formation regulated by nHDL, dHDL, and r-ApoM-S1P, which was consistent with our findings regarding cell proliferation (Fig. 3f, g; Supplementary Fig. S7g). Moreover, knocking down KLF5 or overexpressing KLF5-ΔPY2 showed similar effects to those of HDRACA knockdown or overexpression on HUVECs proliferation and tube formation (Supplementary Fig. S8a–d). Taken together, our data showed that nHDL and r-ApoM-S1P promoted cell proliferation and tube formation by downregulating HDRACA in endothelial cells, whereas dHDL had less of an effect on decreasing HDRACA levels, resulting in less effective promotion of endothelial proliferation and tube formation.

### HDRACA hinders the interaction between RAIN and vigilin in endothelial cells

lncRNAs are known to regulate the expression of neighboring protein-coding genes. Many protein-coding genes are located in the region [−200 to +200 bp] around MRFAP1P1. However, the transfection of HUVECs with HDRACA-siRNAs did not affect the levels of these genes (Supplementary Fig. S9; Supplementary Fig. S15h). These data suggest that HDRACA does not affect the transcription of nearby protein-coding genes in cis.

**Fig. 4:**
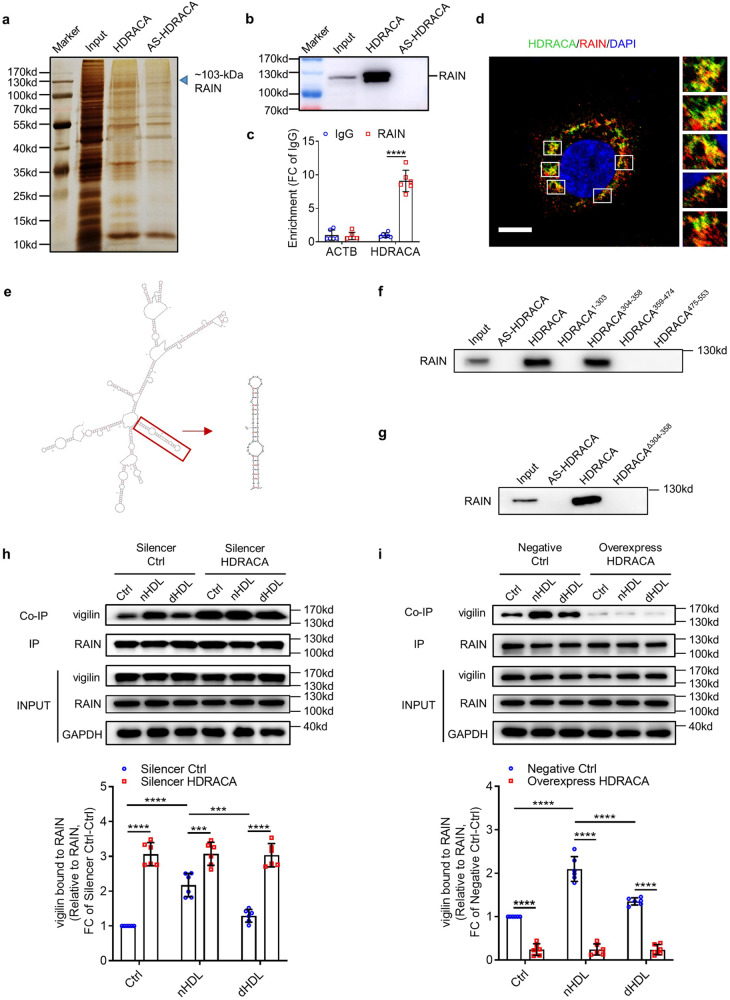
HDRACA hinders the interaction between RAIN and vigilin in endothelial cells. **a** Silver staining of proteins bound to HDRACA or antisense for HDRACA in HUVECs after RNA pull-down. RAIN (arrow) was identified by mass spectrometry. AS-HDRACA means antisense for HDRACA. **b** Immunoblotting for RAIN on protein lysate from HUVECs after RNA pull-down. **c** RIP assays demonstrated the interaction of HDRACA and RAIN in HUVECs. ACTB was used as a negative control. **d** Confocal images showing the colocalization of HDRACA (green) and RAIN (red) in HUVECs. The nuclei were stained with DAPI (blue). Scale bars, 10 μm. **e** Predicted secondary structure of HDRACA and HDRACA^304–358^ by Mfold software. **f** RNA pull-down assays determined the interaction between HDRACA domain constructs and RAIN. **g** RNA pull-down analysis of biotinylated HDRACA nucleotides 304–358 deletion construct and RAIN. **h**, **i** Immunoprecipitation analysis of the interaction of RAIN and vigilin in HUVECs treated with nHDL or dHDL after silencing (**h**) or overexpressing (**i**) HDRACA. Representative plots (up) and quantitation (down) are shown. Data are presented as the mean ± SD. For (**b**–**i**), *n* = 6. ***p < 0.001; ****p < 0.0001

Next, we examined the trans role of HDRACA. Previous studies have reported that the regulatory roles of lncRNAs are closely related to its subcellular localization.^44^ HDRACA is located in the cytoplasm of HUVECs; thus, it may interact with cytoplasmic RNAs and proteins to perform its biological functions. As the abundance of HDRACA in HUVECs was not high enough to sponge microRNAs, we did not consider it to be a competitive endogenous RNA (ceRNA).^45^ RNA pull-down assays were performed using biotinylated HDRACA, followed by mass spectrometry. Among the proteins specifically associated with HDRACA, RAIN was one of the most enriched proteins (Fig. 4a; Supplementary Table S3). Previous studies have reported that RAIN plays an essential role in endothelial cell morphogenesis and blood vessel tubulogenesis.^46,47^ The interaction between RAIN and HDRACA was confirmed using RNA pull-down and RNA immunoprecipitation (RIP) assays (Fig. 4b, c). In addition, HDRACA FISH and RAIN immunofluorescence demonstrated the co-localization of HDRACA with RAIN in the cytoplasm of HUVECs (Fig. 4d). EdU incorporation assay results verified that the knockdown of RAIN inhibited nHDL-induced HUVECs proliferation (Supplementary Fig. S10a). To further determine the segment of HDRACA that interacts with RAIN, we constructed a series of truncated versions of HDRACA according to the secondary structure and found that nucleotides 304–358 of HDRACA form a stem-loop structure and are the only motifs that bind to RAIN (Fig. 4e, f; Supplementary Fig. S10b). Deleting nucleotides 304–358 of HDRACA abrogated the interaction between HDRACA and RAIN (Fig. 4g), suggesting that this segment participates in binding to RAIN.

However, the silencing or overexpression of HDRACA did not affect RAIN protein levels (Supplementary Fig. S10c, d). It has been reported that lncRNAs affect the interactions between proteins.^48,49^ After silencing HDRACA, we performed co-immunoprecipitation (Co-IP) with RAIN, followed by mass spectrometry (Supplementary Fig. S10e; Supplementary Tables S4 and S5). Among significantly different proteins (log_2_(normalized ratio Silencer HDRACA/Silencer Ctrl) > log_2_(1.5) or <−log_2_(1.5) and unique peptides ≥2), vigilin and keratin type I cytoskeletal 9 (KRT9) were the top two enriched proteins (ranked based on MS2 spectral counts). KRT9 is involved in keratin filament assembly; however, little is known about its other functions. Vigilin, also termed HDL-binding protein (HDLBP), was upregulated after silencing HDRACA (Supplementary Table S4). Previous studies have indicated that vigilin can regulate post-transcriptional gene expression with RNA-binding potential.^50,51^ It is likely that vigilin is involved in the regulation of cell cycle-related gene expression. Co-IP assays confirmed that vigilin was bound to RAIN (Supplementary Fig. S10f). nHDL enhanced the interaction between RAIN and vigilin; however, dHDL was less effective than nHDL (Fig. 4h, i). Silencing HDRACA promoted RAIN binding to vigilin (Fig. 4h), whereas overexpression of HDRACA significantly hindered the interaction between RAIN and vigilin and attenuated the ability of nHDL to enhance the interaction between RAIN and vigilin (Fig. 4i). Moreover, knocking down KLF5 or overexpressing KLF5-ΔPY2 showed similar effects as HDRACA knockdown or overexpression on the interaction between RAIN and vigilin (Supplementary Fig. S10g, h).

To investigate the mechanism by which HDRACA disrupts the interaction between RAIN and vigilin, we identified the binding sites between RAIN with HDRACA and vigilin. RAIN contains an N-terminal Ras-associating (RA) domain and a C-terminal diluting (DIL) domain. We transduced HUVECs with lentiviruses encoding FLAG-tagged full-length or truncated RAIN (F1:1-259; F2:260-599; F3:600-963) (Supplementary Fig. S11a). RIP and Co-IP assays showed that both HDRACA and vigilin bound to the F3 segment of RAIN (Supplementary Fig. S11b, c), which contains the DIL domain that binds to cargo, such as protein complexes and RNA.^52^ Therefore, to evaluate whether HDRACA and vigilin bind to the DIL domain of RAIN, we transduced HUVECs with lentiviruses encoding the FLAG-tagged DIL domain of RAIN. RIP assays verified that HDRACA bound to the DIL domain of RAIN (Supplementary Fig. S11d), and co-IP assays indicated that vigilin also bound to the DIL domain of RAIN (Supplementary Fig. S11e). Moreover, silencing of HDRACA increased the interaction between the DIL domain of RAIN and vigilin, whereas overexpression of HDRACA hindered their interaction (Supplementary Fig. S11f, g). This finding indicated that HDRACA bound to the DIL domain of RAIN to interrupt the interaction between the DIL domains of RAIN and vigilin.

### HDRACA increases the inhibitory effect of vigilin on PCNA expression in endothelial cells

**Fig. 5:**
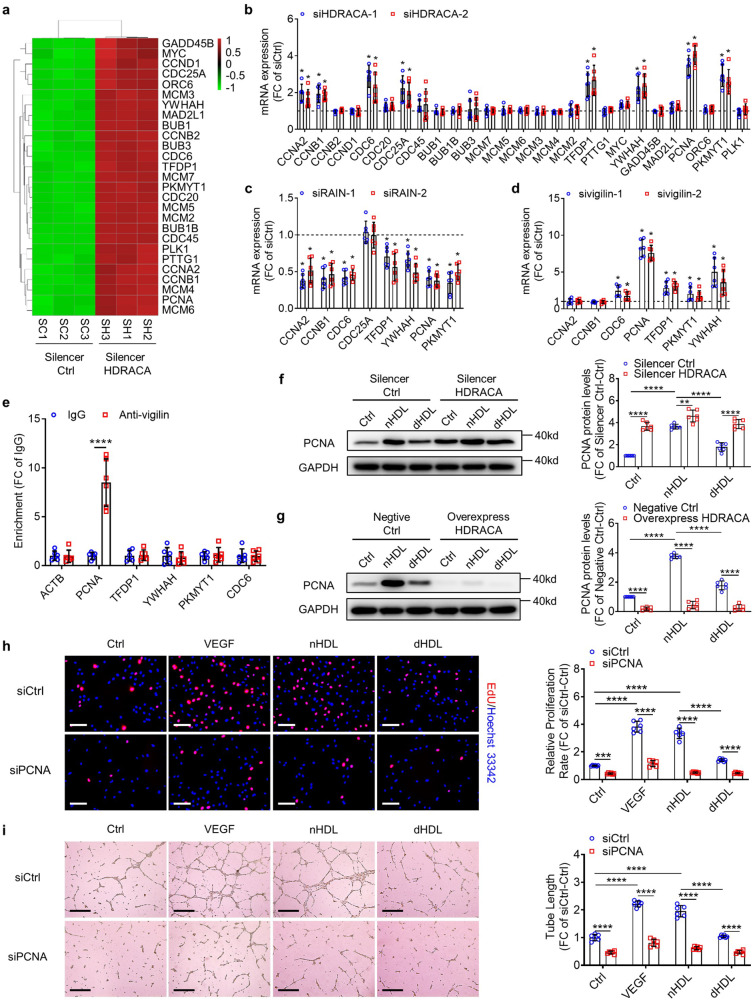
HDRACA increases the inhibitory effect of vigilin on PCNA expression in endothelial cells. **a** Heatmap shows the differentially expressed cell cycle genes after transfection with Silencer Ctrl or Silencer HDRACA. **b** Cell cycle genes expression in HUVECs after transfection with negative control siRNA or HDRACA-siRNAs were determined by RT-qPCR. **c** RT-qPCR determined the expression of cell cycle genes upregulated>1.5 Fold in panel (**b**) after transfection with negative control siRNA or RAIN-siRNAs. **d** The expression of cell cycle genes differentially expressed in panel (**c**) after transfection with negative control siRNA or Vigilin-siRNAs was determined by RT-qPCR. Data in panel (**b**–**d**) were normalized to the negative control siRNA group (siCtrl), and the dotted lines represent the value of the siCtrl. **e** The binding of vigilin protein with PCNA, TFDP1, YWHAH, PKMYT1, or CDC6 mRNAs in HUVECs was determined by RIP assays. **f**, **g** The representative plots (left) and quantitation (right) of immunoblot analysis of PCNA in HUVECs treated with nHDL or dHDL after silencing (**f**) or overexpressing (**g**) HDRACA. **h** The representative images (left) and quantification (right) of EdU incorporation assay in HUVECs treated with VEGF or nHDL or dHDL after transfection with negative control siRNA or PCNA-siRNA (siPCNA-2). The proliferative HUVECs were labeled with EdU (red) and the nuclei were stained with Hoechst 33342 (blue). Scale bars, 100 μm. **i** The representative images (left) and quantification (right) of tube formation assay in HUVECs treated with VEGF or nHDL or dHDL after transfection with negative control siRNA or PCNA-siRNA (siPCNA-2). Scale bars, 500 μm. Data are presented as the mean ± SD. For (**b**–**i**), *n* = 6. For (**b**–**d**), *p < 0.05 compared to siCtrl. For (**e**–**i**), **p < 0.01; ***p < 0.001; ****p < 0.0001

Studies have revealed that vigilin has 14 or 15 RNA-binding hnRNP K homologous (KH) domains and functions in chromosome maintenance, mRNA stability, regulation of mRNA location, and regulation of protein translation.^50,53^ Accordingly, we speculated that the HDRACA-RAIN-vigilin interaction regulated the expression of genes involved in the cell cycle, according to the KEGG analysis (Fig. 5a). To further explore the effect of the HDRACA-RAIN-vigilin interaction on these genes, we performed a series of RT-qPCR assays. HDRACA knockdown elevated the mRNA levels of the cell cycle-related genes CCNA2, CCNB1, CCNB2, CDC6, CDC25A, TFDP1, MYC, YWHAH, MAD2L1, PCNA, and PKMYT1 (Fig. 5b). We selected the genes whose expression increased by more than 50% after HDRACA knockdown for further studies. Among the upregulated genes, RAIN knockdown downregulated the mRNA levels of CCNA2, CCNB1, CDC6, TFDP1, YWHAH, PCNA, and PKMYT1 (Fig. 5c; Supplementary Fig. S15i, s), whereas knockdown of vigilin upregulated the mRNA levels of CDC6, PCNA, TFDP1, PKMYT1, and YWHAH (Fig. 5d; Supplementary Fig. S15j, t).

To date, the most reported functional mechanism of vigilin involves binding to mRNAs and regulating mRNA stability and translation. RIP assays confirmed that the vigilin protein bound to PCNA mRNA but not to HDRACA in HUVECs (Fig. 5e; Supplementary Fig. S12a). Knockdown of KLF5 inhibited the interaction between vigilin and PCNA mRNA in HUVECs, whereas overexpression of HDRACA promoted their interaction (Supplementary Fig. S12b, c). We overexpressed RAIN by transducing HUVECs with RAIN-overexpressing lentivirus (Supplementary Fig. S15w). Overexpression of RAIN in HUVECs inhibited the interaction between vigilin and PCNA mRNA (Supplementary Fig. S12d).

We evaluated the regulatory effects of vigilin on PCNA mRNA stability and translation. We initially transfected HUVECs with negative control siRNA or viglin siRNAs. We then treated the cells with the transcription inhibitor α-amanitin. As shown in Supplementary Fig. S12e, PCNA mRNA levels were gradually decreased by inhibiting transcription; however, knockdown of vigilin effectively decelerated PCNA mRNA decay. Next, we treated the transfected cells with the translation inhibitor cycloheximide and sedimented RNAs at a high velocity through a sucrose gradient (Supplementary Fig. S12f). The majority of PCNA mRNA was associated with polysomes, and the knockdown of vigilin did not affect the relative distribution (Supplementary Fig. S12g). These data suggest that vigilin regulates the expression of PCNA mRNA by decreasing its stability instead of affecting its translation.

nHDL increased the expression of PCNA protein, but dHDL were less effective than nHDL (Fig. 5f, g). Silencing HDRACA effectively enhanced the expression of PCNA protein (Fig. 5f), whereas overexpression of HDRACA downregulated PCNA protein expression and attenuated the ability of nHDL to increase PCNA protein expression (Fig. 5g). We further performed EdU incorporation and tube formation assays to determine the role of PCNA in nHDL- and dHDL-regulated endothelial cell proliferation and tube formation. PCNA knockdown inhibited HUVEC proliferation and tube formation and attenuated the promoting effect of nHDL (Fig. 5h, i; Supplementary Fig. S15k, u).

These data suggest that nHDL enhances the interaction between RAIN and vigilin, contributing to the promotion of PCNA expression and PCNA-mediated angiogenesis, but dHDL is less effective in enhancing the interaction between RAIN and vigilin, which resultes in less effective promotion of PCNA expression and PCNA-mediated angiogenesis.

### Ectopic expression of human HDRACA in mouse endothelial cells inhibits angiogenesis

**Fig. 6:**
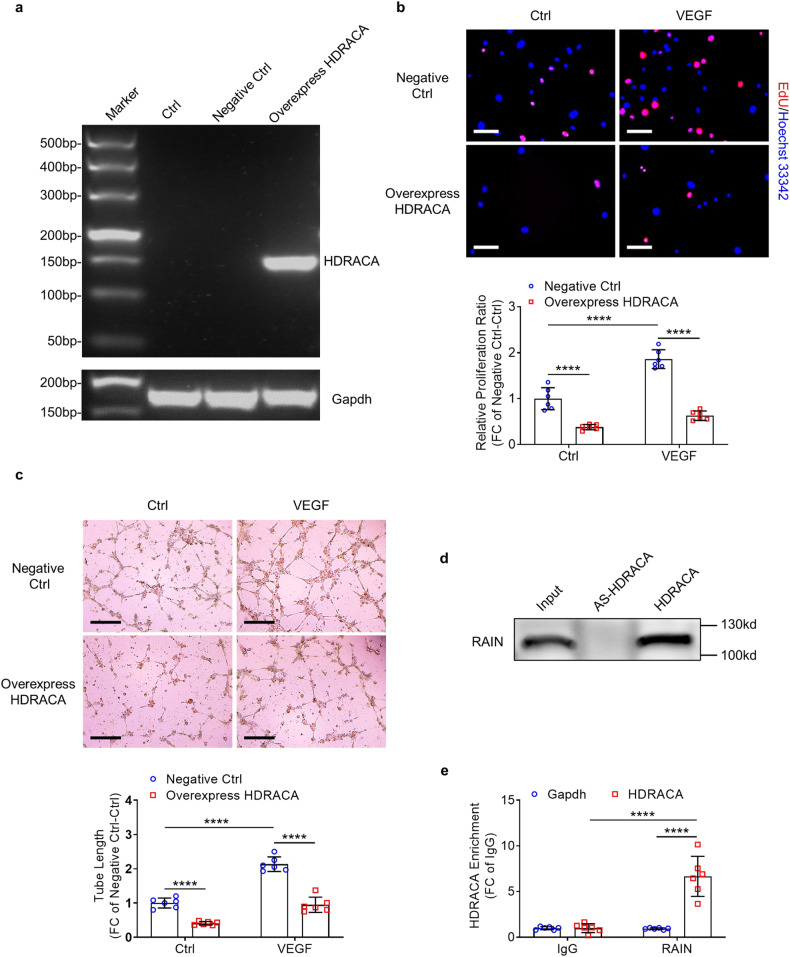
Ectopic expression of human HDRACA in mouse endothelial cells inhibits angiogenesis. **a** RT-PCR indicated the presence of human HDRACA in mFAECs transfected with lentiviruses. **b** The representative images (up) and quantification (down) of EdU incorporation assays in mFAECs treated with or without VEGF after overexpressing HDRACA. The proliferative mFAECs were labeled with EdU (red) and the nuclei were stained with Hoechst 33342 (blue). Scale bars, 100 μm. **c** The representative images (up) and quantification (down) of tube formation assays in mFAECs treated with or without VEGF after overexpressing HDRACA. Scale bars, 500 μm. **d** Immunoblotting for RAIN on protein lysate from mFAECs after RNA pull-down with biotin-labeled HDRACA or HDRACA antisense (AS-HDRACA). **e** RIP assays demonstrated the interaction between HDRACA and RAIN in mFAECs transfected with HDRACA-overexpressing lentiviruses. Gapdh was used as a negative control. Data are presented as the mean ± SD. *n* = 6. ****p < 0.0001

HDRACA is poorly conserved across species. We could not find a clear orthologous counterpart in the mouse genome via BLAST (Basic Local Alignment Search Tool). Therefore, we ectopically expressed human HDRACA in mouse femoral artery endothelial cells (mFAECs) to verify its regulatory effect on angiogenesis. We successfully expressed human HDRACA in mFAECs by using HDRACA-overexpressing lentiviruses (Fig. 6a). HDRACA overexpression inhibited mFAEC proliferation and tube formation (Fig. 6b, c). Because the protein sequence of RAIN is highly conserved and identical between humans and mice, we examined whether human HDRACA could bind to RAIN in mFAECs. RNA pull-down and RIP assays confirmed that human HDRACA could also bind to mouse RAIN in mFAECs (Fig. 6d, e). These results indicated that HDRACA inhibited endothelial angiogenesis in humans and mice.

### HDRACA inhibits angiogenesis in vivo

**Fig. 7:**
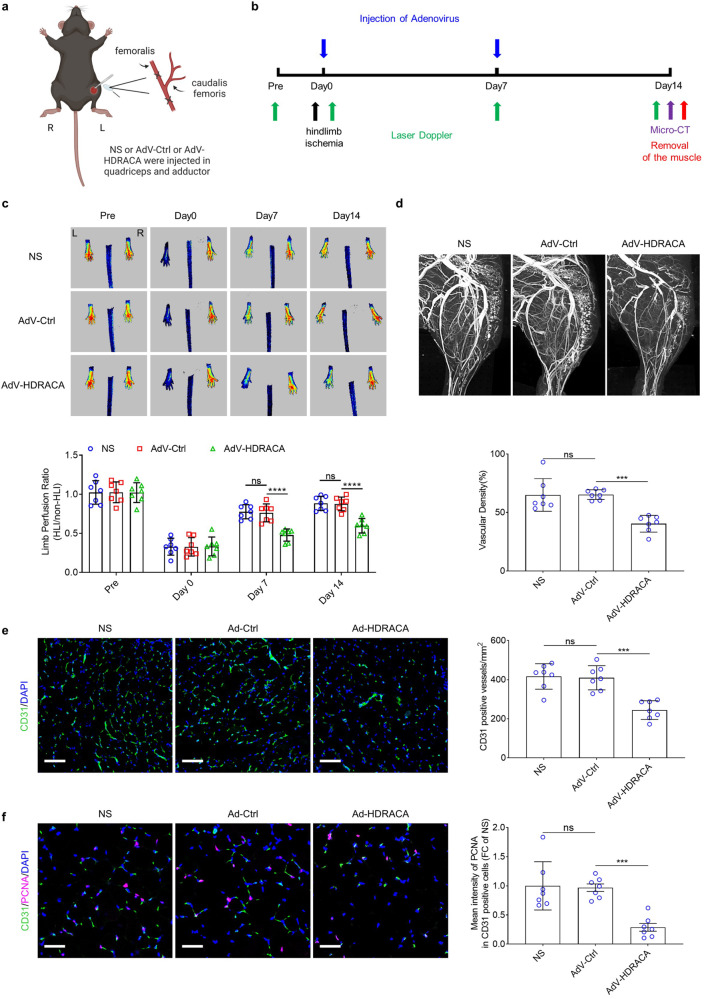
HDRACA inhibits angiogenesis in vivo. **a** Schematic graphic of the ischemia model used in this study. **b** Overview of experimental process in vivo. **c** Representative images (up) and quantification (down) of laser doppler flow before and at various time points after femoral artery ligation in mice treated with normal saline (NS), control adenovirus vector (AdV-Ctrl), or adenovirus vector carrying HDRACA (AdV-HDRACA). R (right) and L (left) foot. **d** Representative images (up) and quantification (down) of micro-CT analysis at day 14 after femoral artery ligation in mice treated with NS or AdV-Ctrl or AdV-HDRACA. **e** Representative images (left) and quantification (right) of CD31 staining (green) at day 14 after femoral artery ligation in mice treated with NS, AdV-Ctrl or AdV-HDRACA. The nuclei were stained with DAPI (blue). Scale bars, 100 μm. **f** Representative images (left) and quantification (right) of CD31 (green) and PCNA (magenta) co-staining at day 14 after femoral artery ligation in mice treated with NS, AdV-Ctrl or AdV-HDRACA. The nuclei were stained with DAPI (blue). Scale bars, 50 μm. Data are presented as the mean ± SD. For (**c**–**f**), *n* = 7. ***p < 0.001; ****p < 0.0001; ns not significant

To verify the regulatory effects of HDRACA in vivo, blood flow in the left hindlimbs of C57BL/6 mice was surgically reduced and we ectopically expressed human HDRACA in these ischemic hindlimbs (Fig. 7a, b). The hindlimbs of the C57BL/6 mice successfully expressed human HDRACA (Supplementary Fig. S13a–c). Laser Doppler perfusion imaging showed that transduction of mice with adenoviral vectors carrying HDRACA (AdV-HDRACA) inhibited blood flow recovery compared with the control vector (AdV-Ctrl) or normal saline (NS) groups (Fig. 7c). Micro-computed tomography (micro-CT) analysis showed that the ectopic expression of HDRACA significantly decreased collateral vessel density in ischemic hindlimbs (Fig. 7d). Immunofluorescence staining of CD31 showed that the ectopic expression of HDRACA decreased capillary density (Fig. 7e). Ectopically expressing HDRACA downregulated PCNA levels in endothelial cells in ischemic hindlimbs without affecting RAIN and vigilin levels (Fig. 7f; Supplementary Fig. S13d–e).

We further confirmed the functional contribution of HDRACA in the regulation of angiogenesis using a Matrigel plug assay. Matrigel mixed with normal saline (NS), control adenovirus vector (AdV-Ctrl), or Adenovirus vector carrying HDRACA (AdV-HDRACA) was subcutaneously injected into the abdomen of mice and removed on day 7 for histological examination (Supplementary Fig. S14a). Matrigel plugs that incorporated AdV-HDRACA showed reduced neovascularization compared with AdV-Ctrl plugs, which was further confirmed by hematoxylin-eosin (HE) staining and CD31 immunohistochemistry (IHC) (Supplementary Fig. S14b–d).

### HDRACA is upregulated in lower extremity artery intima of patients with arteriosclerosis obliterans

**Fig. 8:**
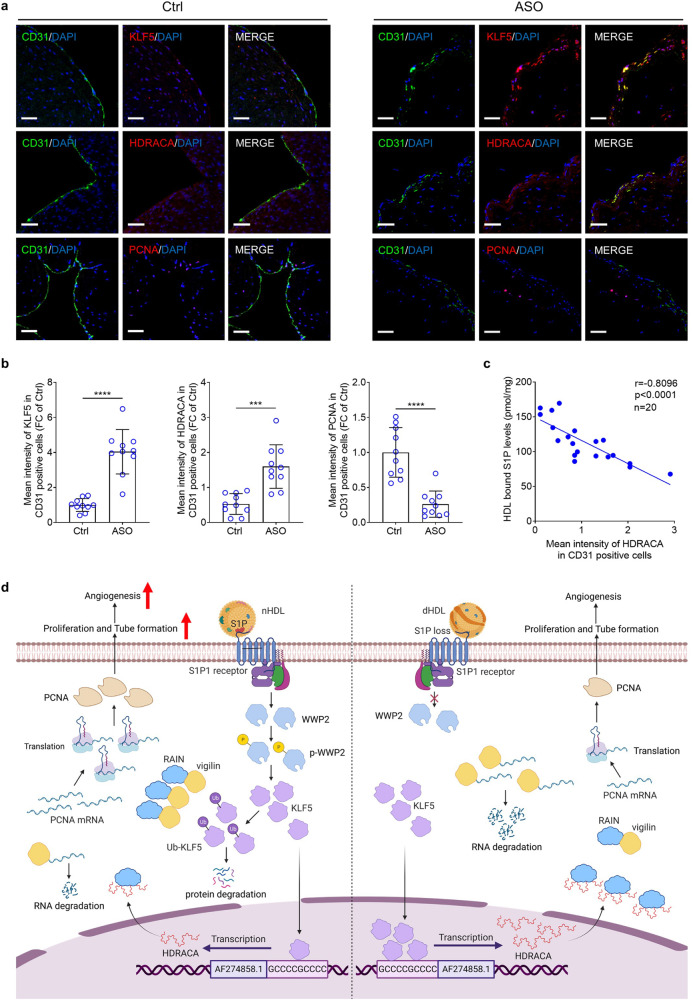
HDRACA is upregulated in lower extremity artery intima of patients with ASO. **a** Representative images of co-stain for CD31 (green) with KLF5 (red; up), HDRACA (red; middle) and PCNA (red; down) in artery tissues from donors for organ transplantation (Ctrl; *n* = 10) and patients with ASO (*n* = 10). The nuclei were stained with DAPI (blue). Scale bars, 50 μm. **b** Quantification of KLF5, HDRACA, or PCNA mean fluorescence intensity in CD31 positive cells of artery tissues from Ctrl and ASO groups. **c** Correlation between S1P content in HDL and HDRACA mean fluorescence intensity in CD31 positive cells of artery tissues. *n* = 20. **d** Graphical illustration of HDL-HDRACA regulatory mechanism. (1) nHDL-bound S1P interacts with S1P1, leading to the phosphorylation and activation of WWP2. The activated WWP2 catalyzes the ubiquitination and subsequent degradation of KLF5, resulting in reduced KLF5-induced HDRACA transcription. Consequently, non-HDRACA-bound RAIN increases and interacts with vigilin to inhibit its binding to PCNA mRNA, leading to increased stability of PCNA mRNA, increased PCNA expression, and increased PCNA-induced endothelial cells proliferation and tube formation, ultimately promoting angiogenesis. (2) dHDL does not inhibit KLF5-induced HDRACA transcription via S1P1-WWP2 signaling pathway, due to the loss of S1P. HDRACA binds to RAIN, which disrupts the interaction between RAIN and vigilin, resulting in the binding of vigilin and PCNA mRNA, thereby decreasing the stability of PCNA mRNA and PCNA expression. As a result, dHDL fails to promote angiogenesis by increasing the expression of PCNA and PCNA-induced proliferation and tube formation. Data are presented as the mean ± SD. ***p < 0.001; ****p < 0.0001; ns not significant

Arteriosclerosis obliterans (ASO) and CAD are manifestations of atherosclerosis that cause arterial stenosis in different parts of the body with similar risk factors and metabolic disorders. To evaluate the potential translational relevance of HDRACA in human tissues, we marked endothelial cells in the lower extremity artery using CD31 immunofluorescence assays, followed by co-staining with KLF5, HDRACA, or PCNA. The endothelial cells of the lower limb arteries in patients with ASO showed upregulation of KLF5 and HDRACA, and downregulation of PCNA (Fig. 8a, b). Furthermore, the S1P content in HDL was inversely correlated with HDRACA levels in the endothelial cells of the lower limb arteries (Fig. 8c). This finding preliminarily confirmed the change in S1P-KLF5-HDRACA-PCNA signaling in ASO.

## Discussion

HDL in patients with CAD undergoes changes in components and becomes dysfunctional (dHDL), as defined by a number of different assays, one of which measures the ability of HDL to induce angiogenesis.^5,54,55^ Some studies have reported a regulatory role of lncRNAs in angiogenesis.^18,56^ In the present study, we showed for the first time that nHDL reduced lncRNA-HDRACA to induce angiogenesis, whereas dHDL from patients with CAD was less able to effectively decrease HDRACA and stimulate angiogenesis.

Previous studies have found that the HDL composition may change in various diseases, including CAD.^29,55,57–59^ For example, HDL in patients with acute coronary syndrome (ACS) contains more proinflammatory proteins, including serum amyloid A protein and complement C3.^59^ The levels of chlorinated Tyr192 and oxidized Met148 in ApoA-I are higher in patients with stable CAD or ACS than in healthy individuals.^55^ The S1P content of HDL in patients with CAD is lower than that in healthy individuals.^29^ Additionally, HDL interacts with endothelial cell receptors to regulate various endothelial activities.^60,61^ In the present study, we found that nHDL and dHDL interacted with S1P1 to regulate HDRACA expression in HUVECs. S1P is the ligand of S1P1.^62^ The interaction between S1P and S1P1 contributes to many important endothelial functions including nitric oxide-dependent vasodilation, endothelial adhesion, and angiogenesis.^9,30,63^ HDL is the primary carrier of S1P in plasma.^28^ We found that nHDL downregulated the expression of HDRACA through S1P1, and dHDL was less effective because it contained less S1P, as reported in previous studies.^9,29^ The plasma levels of both S1P and HDL were lower in patients with CAD than in healthy individuals; however, HDL levels decreased more than S1P levels. This suggests that the lower S1P content in dHDL is not simply due to an endogenous S1P deficiency. A previous study suggested that the cause of lower S1P levels in dHDL might be HDL oxidation or abnormal S1P distribution in HDL subfractions in CAD.^9^ Another potential cause is the competition between S1P and other ApoM-bound lipids. Patients with CAD often experience disturbances in lipid metabolism, especially hyperlipidemia. Fatty acids, including myristic, palmitic, and stearic acids have been shown to compete with S1P for the lipophilic pocket of ApoM.^64^ These lipids may occupy the binding sites of S1P in ApoM, leading to the release of S1P from HDL in CAD. Overall, the decrease in S1P levels of HDL in CAD is likely to be multifactorial. However, because S1P in HDL is responsible for many pleiotropic functions of HDL, a low S1P level is a critical characteristic of HDL dysfunction in CAD.^65^ ApoM affects the biological functions of S1P, and ApoM-bound S1P exerts more potent effects on S1P1 in endothelial cells.^30,60,66^ Here, we showed that ApoM enhanced the inhibitory effect of S1P on HDRACA in endothelial cells. Previous studies indicated that HDL induces angiogenesis through S1P3 activation in endothelial cells.^10,67^ However, we showed that S1P3 did not involve the impact of HDL on HDRACA.

To explore how nHDL downregulates HDRACA expression, we searched for transcription factors that potentially bind to the HDRACA promoter. KLF5 regulates various functions related to cell cycle, angiogenesis, and inflammation.^31,68^ In the present study, we found that nHDL induced KLF5 protein degradation by increasing its ubiquitination and that dHDL was less effective than nHDL in inducing KLF5 ubiquitination. In contrast to dHDL, nHDL carried sufficient S1P to interact with S1P1, increase the ubiquitination and degradation of KLF5, and inhibit HDRACA transcription in endothelial cells.

We found that nHDL downregulated HDRACA to increase the interaction between RAIN and vigilin, resulting in a reduction in the binding between vigilin protein and PCNA mRNA. The interaction between RAIN and vigilin may change the domains of the vigilin protein by catalyzing modifications and attenuating the RNA-binding capacity of vigilin. RAIN can also directly bind to the RNA-binding site of vigilin, thereby competitively preventing vigilin from adsorbing RNA. However, the detailed mechanisms require further investigation. The role of vigilin in mRNA stability is to restrict the access of effector proteins to target mRNAs.^50^ Consistent with many KH-proteins, vigilin can bind to the pyrimidine rich 3′ untranslated region (3′ UTR) to inhibit the binding of ribonucleases that catalyze mRNA degradation.^50^ However, vigilin can also compete with other RNA-binding proteins for binding sites on mRNA, thereby preventing other RNA-binding proteins from stabilizing mRNA.^50^ In the present study, we found that vigilin destabilizes PCNA mRNA expression. RNA-binding proteins and non-coding RNA, including hnRNPLL and PCNA-AS1, increase PCNA mRNA stability.^69,70^ Therefore, it is possible that vigilin interrupts their interactions with PCNA mRNA.

PCNA is a well-known marker of cell proliferation. PCNA is an essential cofactor for DNA polymerase in replication and can induce different DNA polymerases to bind to DNA at different stages of the cell cycle and promote DNA replication and cell cycle progression.^71^ In the present study, we found that nHDL downregulated HDRACA to increase PCNA expression and promote angiogenesis, whereas dHDL was less effective in increasing PCNA expression and promoting angiogenesis. nHDL can increase the expression of PCNA in endothelial cells, and the promoting effect of nHDL on PCNA expression is significantly attenuated by pre-treatment with myeloperoxidase.^72^ Myeloperoxidase oxidation of HDL may lead to S1P reduction in HDL.^9^ One possible factor that may contribute to the limited effect of dHDL in increasing PCNA expression levels is the direct interaction between HDRACA and PCNA mRNA. LncRNA-mRNA interactions can regulate alternative mRNA splicing, stability, and translation.^20^ Some lncRNAs mediate the degradation of its bound mRNA.^73,74^ However, our RNA antisense purification (RAP) assays results suggested no significant interaction between HDRACA and PCNA mRNA in dHDL-treated HUVECs (Supplementary Fig. S12h). It is possible that these interactions are unstable or temporary. Therefore, we were unable to detect this phenomenon. Another possible factor is the indirect effects of HDRACA on PCNA gene transcription, which includes the recruitment of transcription factors, binding of RNA polymerase, and chromatin modification.^21^ Thus, we transfected HUVECs with pGL4.1 reporter plasmid carrying the 2000 bp upstream promoter region of the PCNA gene. However, the luciferase reporter assay did not show a significant change in PCNA promoter activity in dHDL-treated HUVECs after silencing HDRACA (Supplementary Fig. S12i). This factor may be involved in complex signal transduction or regulatory elements. Therefore, we can not exclude the possibility that HDRACA directly interacts with PCNA mRNA or indirectly affects PCNA transcription. However, this issue remains to be addressed.

Although our previous study demonstrated that nHDL and dHDL differentially regulate miR-24-3p levels via SRB1 to affect angiogenesis,^5^ previous studies also indicated that lncRNAs could regulate miRNAs to perform their functions.^24,25^ Therefore, we investigated whether HDRACA regulates miR-24-3p expression to affect angiogenesis. However, we found that neither silencing nor overexpression of HDRACA affected miR-24-3p expression in the endothelial cells (Supplementary Fig. S12j, k). Furthermore, SRB1 knockdown did not affect nHDL and dHDL regulating HDRACA. These data suggest that HDRACA does not regulate miR-24-3p expression in the endothelial cells. HDRACA and miR-24-3p may be regulated by different HDL components.

Although we could not recognize the mouse homology of HDRACA based on sequence conservation, human and mouse RAIN were highly conserved. lncRNAs with low sequence conservation may be conserved at other levels, including secondary or high-order structures, short motifs for protein binding, and positions and functions of lncRNAs.^75^ Compared with the conservation of sequences, the conservation of structure is more important for qualitative analysis of RNA function.^76^ RNA is more conserved among species in terms of structure.^76^ These structures overlapped significantly with the functional elements of RNA. This implies that although HDRACA has no RNA homology with similar sequences in mice, there may be endogenous RNA in mouse endothelial cells with a structure similar to that of HDRACA’s RAIN-binding motif and a similar regulatory function on RAIN. Therefore, we hypothesized that human HDRACA binds to mouse RAIN and exerts a similar regulatory effect on angiogenesis. Other studies have shown that human lncRNAs with poorly conserved sequences can bind to homologous mouse proteins and regulate the biological behavior of mouse cells.^49,77^

One of the important findings of the present study was that the level of HDRACA in the lower extremity artery intima was significantly higher in the group of patients with ASO than in the control group. In addition to the in vitro data, we demonstrated in vivo that HDRACA could inhibit angiogenesis and blood flow recovery in a mouse model of hindlimb ischemia. We and others have previously shown that the proinflammatory properties of HDL can be partly reduced by statins, which are prescribed to almost all patients with CAD.^78,79^ To avoid the influence of medications, we selected patients who were diagnosed with CAD for the first time and without statin treatment three months before HDL isolation. However, we could not avoid the influence of medications in patients with ASO because they were diagnosed with arteriosclerosis, and all had been treated with statins. Therefore, statins may partly decrease the proinflammatory properties of HDL in patients with ASO, which may affect the expression of HDRACA in the lower extremity artery intima. Importantly, although patients with ASO were treated with statins, the levels of HDRACA in the lower extremity artery intima were still significantly higher than those in controls, indicating that the levels of HDRACA in the atherosclerotic intima may be even higher in patients without statin treatment.

In summary, the present study provides direct evidence that nHDL increases the ubiquitination of KLF5 to downregulate its protein levels, which inhibit the expression of HDRACA through S1P1. As a result, the interaction between RAIN and vigilin increased and the binding between vigilin and PCNA mRNA decreased, which increased the expression of PCNA to stimulate angiogenesis. In contrast, dHDL is far less effective in stimulating angiogenesis. Our findings reveal a novel mechanism by which nHDL induces angiogenesis and explain why dHDL does not induce angiogenesis.

## Materials and methods

A detailed description of the methodology is provided in the Online Expanded Materials and Methods section.

### Study populations and sample acquisition

Peripheral venous blood for the isolation of HDL was drawn from sex- and age-matched patients with CAD or healthy human participants (Supplementary Table S1). Arterial samples were acquired from patients with ASO and control participants (without arteriosclerosis) (Supplementary Table S2). This study was approved by the Ethics Review Board of the First Affiliated Hospital, Sun Yat-sen University. Written informed consent was obtained from all the participants.

### HDL isolation

HDL was isolated from healthy individuals or patients with CAD via sequential ultracentrifugation as described previously,^5,26^ and 100 μg/ml of HDL was used unless otherwise stated.

### Cell culture

Human and mouse endothelial cells were cultured in endothelial cell medium and HEK-293T cells were cultured in DMEM. Different types of vascular cells were cultured to examine the HDRACA distribution. All the cells were cultured at 37 °C, 5% CO_2_.

### Cell transfection and transduction

siRNAs, ASONs, or lncRNA Smart Silencer were transfected into HUVECs to interfere with or silence the expression of specific lncRNAs or mRNAs using Lipofectamine RNAiMAX Transfection Reagent (Cat. 13778030, USA, Thermo Fisher Scientific) according to the manufacturer’s instructions. HUVECs were transduced with lentiviral particles (multiplicity of infection (MOI) of 5) to overexpress KLF5-ΔPY2, HDRACA or RAIN.

### RNA isolation, reverse transcription, RT-qPCR and RT-PCR analysis

RNA was extracted and reverse transcribed into cDNA. RT-qPCR analysis was performed to quantify the expression levels of lncRNAs, mRNAs and microRNAs.

Reverse transcription PCR (RT-PCR) was performed to evaluate whether human HDRACA was successfully expressed in mouse endothelial cells or vessels. The extracted RNA was reverse transcribed, amplified, and subjected to DNA agarose gel electrophoresis.

### LncRNA identification and characterization

5′ and 3′ RACE assays were performed to acquire the transcriptional initiation and termination sites of ENST00000562749.1. For the 3′ RACE assay, extracted RNA was polyadenylated, reversely transcripted and amplified with specific primers. For the 5′ RACE assay, the first strand of cDNA was tailed with polycytidine and amplified with specific primers. The amplified cDNA was sequenced.

The coding potential of HDRACA was also predicted and verified. The ORFs were cloned upstream of the p3xFLAG-CMV-14 expression vector, transfected into HEK-293T cells, and immunoblotted with the FLAG antibody.

To determine the subcellular location of HDRACA in HUVECs, nuclear and cytoplasmic fractions were isolated using a PARIS kit (Cat. AM1921, USA, Thermo Fisher Scientific) according to the manufacturer’s instructions.

### ddPCR

ddPCR was performed to quantify HDRACA levels in endothelial cells, vascular smooth muscle cells, fibroblasts, and macrophages.

### Preparation and quantification of S1P and ApoM

Excess S1P was mixed with r-ApoM to form the r-ApoM-S1P complex. HDL was directly loaded with S1P as previously described.^9^ S1P in HDL and the r-ApoM-S1P complex were extracted via one-step methanol precipitation and quantified by liquid chromatography-tandem mass spectrometry (LC-MS/MS) as previously described.^80^ ApoM in the r-ApoM-S1P complex was measured by an enzyme-linked immunosorbent assay as previously described.^81^

### Immunoblot analysis

The immunoblot analysis was performed as previously described.^82^

### Immunoprecipitation

Immunoprecipitation was performed using protein A/G magnetic beads (Cat. B23201, USA, Bimake), according to the manufacturer’s instructions.

### LncRNA microarrays

Arraystar LncRNA Expression Microarray was performed by Kangchen Biotech (Shanghai, China) to detect HDL-bound lncRNAs.

### mRNA sequencing

HUVECs were transfected with lncRNA Smart Silencer targeting HDRACA and Negative Control Smart Silencer. RNAs were extracted from the HUVECs and subjected to poly-A-seq. PolyA-seq and data analyses were performed by RiboBio (Guangzhou, China).

### Cell cycle and apoptosis analysis

The cell cycle was detected using the CellCycle PI/RNAase Staining Solution (Cat. A056, USA, ABP Biosciences), according to the manufacturer’s instructions. Apoptosis was determined using Annexin V-FITC/PI staining and TUNEL assays. Annexin V-FITC/PI staining was performed using an Annexin V-FITC/PI Apoptosis Detection Kit (Cat. E606336-0100, China, BBI Life Sciences) according to the manufacturer’s instructions. TUNEL assays were performed using riboAPO^TM^One-Step TUNEL Apoptosis Kit (Cat. C11026-1, China, RiboBio) according to the manufacturer’s instructions.

### FISH and ISH assays

For FISH assays, cultured cells or frozen sections were hybridized with HDRACA detection probes to determine the subcellular location of HDRACA in HUVECs and to detect HDRACA expression in HUVECs or adductor muscles of C57BL/6 mice.

### Immunofluorescence assays

Immunofluorescence assays were performed with a RAIN antibody to analyze the co-localization of HDRACA and RAIN in HUVECs after being subjected to FISH of HDRACA. Capillaries in tissue sections of the adductor muscles were stained with the CD31 antibody. FISH of HDRACA was performed after immunofluorescence assays for CD31 to examine whether HDRACA was successfully expressed in muscle endothelial cells. Co-staining of CD31 with RAIN, vigilin and PCNA was performed to detect the levels of these indicators in the endothelial cells of the adductor muscles. Co-staining for CD31 with KLF5, HDRACA, and PCNA was performed to detect the levels of these indicators in the endothelial cells of the lower extremity artery.

### ChIP and luciferase reporter assay

ChIP and luciferase reporter assays were performed to examine the interaction between KLF5 and the HDRACA promoter. ChIP assays were performed using the SimpleChIP Plus Enzymatic Chromatin IP Kit (Cat. 9005, USA, Cell Signaling Technology) according to the manufacturer’s instructions. Luciferase reporter assays were performed using reporter plasmids containing deletions in the HDRACA promoter constructs and mutation of KLF5 binding sites according to the manufacturer’s instructions for the Dual-Lucy Assay Kit (Cat. D0010, China, Solarbio).

### Endothelial cell proliferation, migration, and tube formation

Cell Counting Kit 8 (Cat. CK04, Japan, Dojindo) and Cell-Light EdU Apollo567 In Vitro Kits (Cat. C10310-1, China, RiboBio) was used to measure HUVEC proliferation according to the manufacturer’s instructions. HUVEC migration and tube formation assays were performed as previously described.^5,83^

### HDRACA and proteins interaction analysis

The interactions between HDRACA and proteins were determined using RNA pull-down and RIP assays. RNA pull-down assays were performed according to the manufacturer’s instructions of Pierce Magnetic RNA-Protein Pull-down Kit (Cat. 20164, USA, Thermo Fisher Scientific). RIP assays were performed using a Magna RIP RNA-Binding Protein Immunoprecipitation Kit (Cat. 17700, USA, Millipore Sigma) according to the manufacturer’s instructions.

### Vigilin protein and PCNA mRNA interaction analysis

The effect of PCNA mRNA binding to vigilin was analyzed using RNA stability and polysome gradient assays.

### Measurements of vascular growth in vivo

The animal experiments were approved by the Ethics Review Board of the First Affiliated Hospital, Sun Yat-sen University (approval number: [2020]019). A model of lower-limb ischemia was established in C57BL/6 mice, which were subjected to intramuscular injections of AdV-HDRACA, AdV-Ctrl, or NS. Recovery of blood flow to the ischemic foot was sequentially monitored using a laser Doppler imaging system. After perfusion with a contrast agent, the hindlimb vasculature was imaged and analyzed using a micro-CT imaging system. In addition, Immunofluorescence assays for CD31 were performed on adductor muscles harvested on day 14 post-surgery to compare the capillary densities of the different groups. Co-staining for CD31 with RAIN, vigilin and PCNA was performed to investigate the changes in these indicators in the adductor muscles.

In vivo Matrigel plug assays with AdV-HDRACA, AdV-Ctrl, or NS were performed in C57BL/6 mice, as previously described, to examine the role of HDRACA in angiogenesis.^84,85^ Hematoxylin and eosin (HE) staining and CD31 IHC were performed to detect neovascularization in the Matrigel plugs.

### Statistical analysis

Data are presented as the mean ± SD. The differences among the test groups were determined with one-way ANOVA followed by Tukey’s test or Kruskal-Wallis test for more than two groups or with Student’s *t*-test or Mann-Whitney test for two groups. *p* < 0.05 was considered statistically significant.

### Supplementary information

Supplementary Table S7

Supplementary Materials

Supplementary Table S6

## Data Availability

Data supporting the findings of this study are available upon reasonable request from the corresponding authors.
