# Supplementary material for: High-density lipoprotein regulates angiogenesis by long non-coding RNA HDRACA
Source: Signal Transduct Target Ther. 2023 Aug 14;8:299. doi: 10.1038/s41392-023-01558-6 (PMC10423722; doi:10.1038/s41392-023-01558-6)
Supplement: Supplementary file 2 — Supplementary Materials [file 41392_2023_1558_MOESM2_ESM.docx]

Supplementary Materials for

**High-density lipoprotein regulates angiogenesis by long non-coding RNA HDRACA**

Zhi-Wei Mo, Yue-Ming Peng, Yi-Xin Zhang, Yan Li, Bi-Ang Kang, Ya-Ting Chen, Le Li, Mary G. Sorci-Thomas, Yi-Jun Lin, Yang Cao, Si Chen, Ze-Long Liu, Jian-Jun Gao, Zhan-Peng Huang, Jia-Guo Zhou, Mian Wang, Guang-Qi Chang, Meng-Jie Deng, Yu-Jia Liu, Zhen-Sheng Ma, Zuo-Jun Hu, Yu-Gang Dong, Zhi-Jun Ou*, Jing-Song Ou*

*Correspondence to

Z.J.O: zhijunou@163.com

J.S.O: oujs@mail.sysu.edu.cn, oujs2000@163.com

**This PDF file includes:**

Expanded Materials and Methods

Supplementary Figures S1 to S15

Supplementary Tables S1 to S5

**Other Supplementary Materials for this manuscript include the following:**

Supplementary Tables S6 and S7.

**Expanded Materials &Methods**

**Study populations and sample acquisition**

Gender and age-matched patients with coronary artery disease (CAD) and healthy volunteers without cardiovascular risk factors were recruited for the isolation of high-density lipoprotein (HDL). Patients are diagnosed as CAD first time with coronary stenosis ≥70% in coronary artery angiography and without statins treatment before three months were chose. Patients were excluded with accompanying infection, diabetes, other inflammatory or autoimmune disorders, advanced kidney or liver failure, neoplastic disorders, or a history of major surgery or trauma within the previous month. Subjects fasted overnight prior to blood collection and peripheral venous blood was acquired. Artery samples were acquired from patients who were confirmed as arteriosclerosis obliterans (ASO) and accepted amputation at the First Affiliated Hospital, Sun Yat-sen University. Control artery samples were acquired from donors for organ transplantation without arteriosclerosis. Detailed information of patients and controls is provided in Supplementary table S1 and S2. Written informed consent to participate in the study was obtained from each participant. Plasma was collected from blood anti-coagulated with ethylene diamine tetraaceticacid (EDTA), and then subject to HDL isolation or stored at -80 ℃ for further use. Artery samples were fixed using 4% paraformaldehyde (PFA) and then were embedded by paraffin for further study. This study was approved by the Ethics Review Board of the First Affiliated Hospital, Sun Yat-sen University (Approval number: [2020]078).

**HDL isolation**

HDL was isolated from healthy individuals or patients with CAD via sequential ultracentrifugation as described previously^1, 2^. Protein concentration of isolated HDL was determined by bicinchoninic acid protein assay (Cat. 23225, USA, Thermo Fisher Scientific). The isolated HDL was stored at 4 ℃ and used within 3 weeks.

**Cell culture and HDL or S1P incubation**

Human umbilical vein endothelial cells (HUVECs; Cat. 8000), human aortic endothelial cells (HAECs; Cat. 6100), human coronary artery endothelial cells (HCAECs; Cat. 6020) and human cardiac microvascular endothelial cells (HCMECs; Cat. 6000) were purchased from ScienCell company, USA. Mouse femoral artery endothelial cells (mFAEC; Cat. MIC-iCell-c013) were purchased from iCell company, China. All types of endothelial cells were cultured in endothelial cell medium (ECM; Cat.1001, USA, ScienCell) containing 5% fetal bovine serum (FBS; Cat.0025, USA, ScienCell), 1% endothelial cell growth supplement (ECGS; Cat.1052, USA, ScienCell) and 1% penicillin/streptomycin (P/S; Cat.0503, USA, ScienCell). HUVECs, HAECs, HCAECs and HCMECs were cultured until the third passage. mFAECs were cultured until the second passage. All the cells were cultured at 37 ℃, 5% CO_2_ and the cell number was determined by cell count plate (Cat. 177-112C, Japan, WATSON).

HEK-293T cells (Cat. CL-0005, China, Procell) were purchased and cultured at 37 ℃, 5% CO_2_ in DMEM medium (Cat. C11995500BT, USA, Gibco) containing 10% FBS (Cat. A3160802, USA, Gibco) and 1% P/S (Cat. 15140-122, USA, Gibco).

Human aortic smooth muscle cells (HASMCs; Cat. 6110, USA, ScienCell) were purchased and cultured at 37 ℃, 5% CO_2_ in smooth muscle cell medium (SMCM; Cat. 1101, USA, Sciencell) containing 2% FBS (Cat. 0010, USA, Sciencell), 1% smooth muscle cell growth supplement (SMCGS; Cat. 1152, USA, Sciencell) and 1% P/S (Cat. 0503, USA, Sciencell).

Human aortic fibroblasts (FB; Cat. CTCC-206-HUM, China, Juno) were purchased and cultured at 37 ℃, 5% CO_2_ in DMEM medium (Cat. C11995500BT, USA, Gibco) containing 10% FBS (Cat. A3160802, USA, Gibco) and 1% P/S (Cat. 15140-122, USA, Gibco).

THP-1 monocytes (Cat. THP-1, China, Procell) were purchased and cultured at 37 ℃, 5% CO_2_ in RPMI Medium 1640 basic (1×) (Cat. C11875500BT, USA, Gibco) containing FBS (Cat. A3160802, USA, Gibco) and 1% P/S (Cat. 15140-122, USA, Gibco). THP-1 monocytes were stimulated by 100 ng/mL Phorbol 12-myristate 13-acetate (PMA) for 48 h to differentiate into macrophages.

Endothelial cells were serum-starved overnight in ECM containing 0.5% FBS prior to incubation with 100 μg protein/ml nHDL or dHDL for 24 h. 50 ng/ml recombinant human vascular endothelial growth factor (VEGF) 165 protein (Cat. 293-VE, USA, [R&D Systems](https://www.rndsystems.com/)) was used in all human endothelial cell studies requiring  VEGF. 50 ng/ml recombinant mouse VEGF 164 protein (Cat. 50159-MNAB, China, Sino Biological) was used in all mouse endothelial cell studies requiring VEGF. As for sphingosine 1-phosphate (S1P) assays, serum-starved endothelial cells were incubated with different concentrations of S1P (Cat. 860492P, USA, Avanti Polar Lipid). The S1P receptor 1 (S1P1) antagonist W146 (Cat. W1020, USA, Sigma-Aldrich) were used at 10 μM in this study, and W146 was added to the cells 1h prior to addition of HDL.

**RNAi and antisense silencing studies**

lncRNA Smart Silencer, designed and synthesized by RiboBio, China, was a mixture of three small interfering RNAs (siRNAs) and three antisense oligonucleotides (ASONs). We used lncRNA Smart Silencer to target HDRACA. Negative Control Smart Silencer was designed as a negative control. All siRNAs and ASONs were also designed and synthesized by RiboBio, China. Negative control siRNAs and ASONs were used as transfection control respectively. The target sequences of lncRNA Smart Silencers, ASONs and siRNAs were listed in Supplementary Table S6.

Cells were transfected with lncRNA Smart Silencer (200 nM), siRNA (100 nM), siRNA (33 nM)+ASON (66 nM) or corresponding negative controls using Lipofectamine RNAiMAX Transfection Reagent (Cat. 13778030, USA, Thermo Fisher Scientific) according to the manufacturer’s instructions.

**RNA extraction, reverse transcription, and RT-qPCR analysis**

Total RNA in cells were extracted using Trizol Reagent (Cat. T9424, USA, Sigma-Aldrich). Reverse transcription was performed with Transcriptor cDNA Synth. kit (Cat. 4897030001, Switzerland, Roche) according to the manufacturer’s instructions. Real-time quantitative PCR (RT-qPCR) was performed in a Bio-rad CFX96 system. The primers used for RT-qPCR were listed in Supplementary Table S6. To quantify gene relative expression, Ct values were calculated with the efficiency-corrected 2^−△△CT^ method normalized to the endogenous reference. For lncRNAs and mRNAs, GAPDH or ACTB were used as endogenous reference. For microRNAs, U6 was used as endogenous reference. Data were presented as fold change relative to control groups.

Total RNA in nHDL and dHDL of equal protein mass or plasma were extracted using Trizol LS Reagent (Cat. 10296028, USA, Invitrogen). Cel-miR-39-3p standard RNA (Cat. miRB0000010, China, RiboBio) were added as before RNA extraction. Reverse transcription with miRNA-specific RT-primers and qRT-PCR was performed as mentioned above. Ct values were calculated and normalized to the cel-miR-39-3p.

**RT-PCR**

Reverse transcription PCR (RT-PCR) was performed as following. The extracted RNA were reversely transcripted into cDNA. Then cDNA was amplified with specific primers using 2×Pro Taq Master Mix (Cat. AG11109, China, Accurate Biology) according to the manufacturer’s instructions. The amplified product was further subjected to DNA agarose gel electrophoresis. The primers used for RT-PCR were consistent with RT-qPCR.

**ddPCR**

Droplet Digital PCR (ddPCR) was performed to determine HDRACA concentration as following. RNA was extracted and reversely transcripted as described in the qRT-PCR. Synthesized cDNA, 2×ddPCR EvaGreen Supermix (Cat. 1864033, USA, bio-rad) and primers were mixed. 20 μl reaction mixture was mixed with 70 μl QX200 Droplet Generation Oil for EvaGreen (Cat. 1864005, USA, bio-rad) in DG8 Cartridge for QX200/QX100 Droplet Generator (Cat. 1864008, USA, bio-rad) applied to the QX200 droplet generator device for droplet generation (Bio-Rad). Next, each sample was transferred into a 96-well ddPCR plate and PCR performed according to the manufacturer’s protocol. At the end of the PCR reaction, the QX200 droplet reader (Bio-Rad) was used to count positive and negative droplets. The fraction of PCR-positive droplets enabled the target to be quantified according to a Poisson distribution. The primers used for ddPCR were consistent with RT-qPCR.

**RACE**

3’ rapid-amplification of cDNA ends (RACE) assays were performed as follow. Extracted RNA was polyadenylated and reversely transcripted. Next, cDNA products were amplified with specific primers by PCR and further subjected to DNA agarose gel electrophoresis. Then, the obtained band was purified and TA-cloned. Finally, identifying clones containing gene-specific inserts by gene specific PCR and sequencing.

5’RACE assays were performed as follow. Firstly, extracted RNA was reversely transcripted by MMLV reverse transcriptase to obtain the first strand of cDNA. Then, poly cytidine tail was added to the first strand of cDNA by terminal deoxyribonucleotidyl transferase (TDT) tailing reaction. Next, the cDNA was amplified by two-step PCR with designed adaptor primers and 5’RACE gene specific primers. Finally, the amplified cDNA was purified and sequenced.

**Protein coding potential**

Coding potential assessment tool (CPAT) was used for prediction of coding potential. Furthermore, NCBI ORFfinder was used to search for the open reading frames (ORFs) of lncRNA candidate. For validation of peptide coding potential, different ORFs were cloned upsteam of p3xFLAG-CMV-14 expression vector. Next, HEK-293T cells were transfected with different ORFs expressing plasmids using Lipofectamine^TM^ 3000 Transfection Reagent (Cat. L3000015, USA, Invitrogen) according to the manufacturer’s instructions. After protein lysate was isolated, immunoblot analysis was performed to detect FLAG tag. MYC was used as a positive control.

**Nuclear/cytoplasm fractionation**

To determine the location of HDRACA in HUVECs, the nuclear and cytoplasmic fractions were isolated with PARIS kit (Cat. AM1921, USA, Thermo Fisher Scientific) according to the manufacturer’s instructions. In brief, 1×10^7^ fresh cultured cells were collected, washed with PBS and placed on ice. The cells were resuspended in cell fractionation buffer, followed by 10 min incubation. After centrifugation, the supernatant (cytoplasmic fraction) was collected and nuclear precipitation (nuclear pellet) was washed in ice-cold cell fractionation buffer. Then lyse nuclear pellet in cell disruption buffer. Next, the lysates were mixed with equal volume of 2X lysis/binding solution and equal volume of 100% ethanol. Draw the sample mixture through a filter cartridge and wash once with wash solution 1 and twice with wash solution 2/3. The RNA was eluted with elution solution. Finally, the eluted RNA was reversely transcripted and RT-qPCR was performed with nuclear and cytoplamic fraction respectively.

**Preparation of r-ApoM-bound S1P**

Recombinant soluble human apolipoprotein M (r-ApoM; residues 23-188, Swiss-Prot entry O95445) was expressed in E.coli, purified from inclusion bodies and refolded as previously described^3^. Eight times excess of sphingosine-1-phosphate (S1P; Cat. 860492, USA, Avanti Polar Lipid) was added to r-ApoM and incubated for 30 min at room temperature to load r-ApoM with S1P. The unbound S1P in the mixture was removed by running through a desalting PD10 column (Cat. 17-0851-01, USA, GE Healthcare) with serum-free M200 medium. The mean S1P/r-ApoM ratio was 1.1. The r-ApoM-bound S1P (r-ApoM-S1P) was stored at -20°C.

**S1P-loading of isolated HDL**

HDL was loaded with S1P as previously described^4^. S1P (Cat. 860492, USA, Avanti Polar Lipid) dissolved in methanol was evaporated under a cell culture flow bank for two hours. 0.1 mg of HDL (at a concentration of 1 mg/mL HDL protein) was added to 6 nmol of S1P, vigorously vortexed and left at room temperature for one hour. After dialysis, the S1P-loaded HDL was subjected to S1P quantification and subsequent assays.

**S1P quantification**

S1P was quantified as previously described^5, 6^. In brief, HDL samples were diluted with tris-buffered saline (50 mM Tris-HCL pH 7.5, 0.15 mM NaCl). The diluted HDL and r-ApoM-S1P were mixed with precipitation solution (methanol containing 20 nM internal standard d7-S1P (Cat. 860659P, USA, Avanti Polar Lipid)), followed by 30 sec of vortexing. The samples were centrifuged at 17000 g for 2 min, and supernatant were injected for liquid chromatography tandem mass spectrometric (LC-MS/MS) analysis.

**ApoM quantification**

ApoM in r-ApoM-S1P complex was measured by enzyme linked immunosorbent assay (ELISA) as previously described^7^

**Immunoblot analysis**

The immunoblot analysis were performed as previously described^8, 9^. In brief, the cells were lysed with 1×RIPA buffer (Cat. 9806S, USA, Cell Signaling Technology) supplemented with proteinase inhibitor. Then, after centrifugation, supernatant was collected and protein concentration was measured by bicinchoninic acid protein assay (Cat. 23225, USA, Thermo Fisher Scientific). Equal amounts of protein mixed with SDS-PAGE loading buffer (Cat. P0015F, China, Beyotime) were loaded and separated by SDS-PAGE. Then, the samples were transferred to PVDF Western Blotting Membranes (Cat. 03010040001, Switzerland, Roche). After blocked with Tris-buffered saline (TBS)/Tween-20 containing 5% bovine serum albumin (BSA), the membranes were probed using the primary antibodies: DYKDDDDK Tag (1:1000; Cat. 14793, USA, Cell Signaling Technology), ATP-binding cassette transporter A1 (ABCA1; 1:500; Cat. ab18180, UK, Abcam), ATP-binding cassette transporter G1 (ABCG1; 1:1000; Cat. Ab52617, UK, Abcam), sphingosine-1-phosphate receptor 1 (S1P1; 1:500; Cat. sc-48356, USA, Santa Cruz Biotechnology), scavenger receptor class B type 1 (SR-BI; 1:500; Cat. ab106572, UK, Abcam), sphingosine-1-phosphate receptor 3 (S1P3; 1:1000; Cat. ab108370, UK, Abcam), Apolipoprotein M (ApoM; 1:2000, Cat. ab91656, UK, Abcam), krueppel-like factor 5 (KLF5; 1:1000; Cat. 61099, USA, Active Motif or 1:1000; Cat.51586, Cell Signaling Technology), WWP2 (1:500; Cat.12197-1-AP, USA, Proteintech), Ubiquitin (1:1000; Cat. 3936, USA, Cell Signaling Technology), Phosphotyrosine (1:500; Cat.05-321, USA, Millipore Sigma), Ras-interacting protein 1 (RAIN, 1:250; Cat. ab129820, UK, Abcam), Vigilin (1:500; Cat. sc-271523, USA, Santa Cruz Biotechnology), proliferating cell nuclear antigen (PCNA, 1:1000; Cat. 13110, USA, Cell Signaling Technology) and GAPDH (1:1000; Cat. 60004-1-Ig, USA, Proteintech) at 4 °C overnight. Next, the membranes were incubated with the appropriate secondary antibodies for 1 h at room temperature (RT). Immunoreactive bands were visualized using ECL (AI600, GE Healthcare, USA).

**Immunoprecipitation**

The immunoprecipitation was performed as following. Cells were lysed in 1×Cell Lysis Buffer (Cat. 9803S, USA, Cell Signaling Technology) supplemented with Proteinase Inhibitor. The protein concentration was measured by bicinchoninic acid protein assay (Cat. 23225, USA, Thermo Fisher Scientific). The antibody or IgG were incubated with prepared Protein A/G magnetic beads (Cat. B23201, USA, bimake) for 30 min at RT. Next, the protein supernatant was incubated with antibody-magnetic beads complexes at 4 ℃ overnight. Then, after washing the complexes, the SDS-PAGE loading buffer were added and the immunocomplexes were heated at 95 °C for 5 min. Finally, the denatured proteins were detected by mass spectrometry or immunoblot analysis. The following antibody was used in this study: RASIP1 antibody (Cat. 17971-1-AP, USA, Proteintech), KLF5 antibody (Cat. 61099, USA, Active Motif) and DYKDDDDK Tag antibody (Cat. 14793, USA, Cell Signaling Technology)

**Arraystar Human LncRNA Arrays**

Arraystar Human LncRNA Arrays were performed by Kangchen Biotech, China to detect the lncRNA profiles in nHDL and dHDL. In brief, total RNA were isolated with Trizol LS Reagent (Cat. 10296028, USA, Invitrogen) and quantified using NanoDrop ND-1000 (Thermo Fisher Scientific, USA). RNA integrity was assessed by Agilent 2100 Bioanalyzer. After removal of rRNA (mRNA-ONLY™ Eukaryotic mRNA Isolation Kit, Epicentre), each sample was amplified and transcribed into fluorescent cRNA along the entire length of the transcripts without 3' bias utilizing a random priming method (Arraystar Flash RNA Labeling Kit, Arraystar). The labeled cRNAs were purified by RNeasy Mini Kit (Qiagen). The concentration and specific activity of the labeled cRNAs (pmol Cy3/μg cRNA) were measured by NanoDrop ND-1000. Each labeled cRNA was fragmented and heated. 50 μl of hybridization solution was dispensed into the gasket slide and assembled to the LncRNA expression microarray slide. The slides were incubated for 17 h at 65°C in an Agilent Hybridization Oven. The hybridized arrays were washed, fixed and scanned using the Agilent DNA Microarray Scanner (part number G2505C). The data was extracted using Agilent Feature Extraction Software. Fold change cut-off of differential expression was |log2FoldChange|>1. Scatter plot of all the lncRNAs detected was generated using RStudio.

**mRNA sequencing**

Extracted RNAs from HUVECs were put into PolyA-seq and analyzed to determine the expression profile of mRNA after HDRACA was knocked down. The polyA-seq and data analysis were performed by RiboBio, China. In brief, total RNA was isolated with Trizol Reagent (Cat. T9424, USA, Sigma-Aldrich) and quantified using NanoDrop ND-1000 (Thermo Fisher Scientific, USA). RNA integrity was evaluated using Agilent 2200 TapeStation (Agilent Technologies, USA). After rRNAs were removed using EpicentreRibo-Zero rRNA Removal Kit (Illumina, USA), the purified RNAs were fragmented and subjected to cDNA synthesis following by adaptor ligation and enrichment with a low-cycle according to instructions of NEBNext Ultra RNA Library Prep Kit for Illumina (NEB, USA). The purified library products were paired-end sequenced (PE150, Sequencing reads were 150 bp) using IlluminaHiSeq 3000 platform. |log2FoldChange|>1 and q-value (Corrected p-value) <0.05 was considered to be the criteria of differential expression. All the differentially expressed genes were used for gene ontology analysis and KEGG pathway analysis. Volcano plot and Heatmap represented differentially regulated genes were generated using RStudio. The sequencing data were provided in the Supplementary Table S7.

**Cell cycle analysis**

Cell cycle was detected using Cell Cycle PI/RNAase Statining Solution (Cat. A056, USA, ABP Biosciences) according to the manufacturer’s instructions. In brief, after washing the harvested HUVECs with PBS, the resuspended pellet were fixed in 70% prechilled ethanol at 4 ℃ for 12 h. Then, the ethanol was removed and cells were washed with PBS again before adding 500 μl Cell Cycle PI/RNAse Staining Solution to the pellet. Finally, the mixture was incubated for 30 min at 37 ℃ away from light followed by flow cytometric analysis (Gallios, Beckman, USA). The data were analyzed by ModFit LT software.

**Apoptosis assay**

Cells were dissociated and collected to determine apoptosis using Annexin V-FITC/PI Apoptosis Detection Kit (Cat. E606336-0100, China, BBI Life Sciences) according to the manufacturer’s instructions. Briefly, the harvested HUVECs were washed with PBS and then resuspended in 1×Binding Buffer. After Annexin V-FITC was added, the mixture was incubated for 15 min at RT away from light. Then the cells were washed and resuspended in 1×Binding Buffer. Finally, the cells were incubated with Propidium Iodide for 5 min and immediately analyzed by flow cytometry (Accuri C6, BD, USA). Data analysis was performed using FlowJo software.

**TUNEL assay**

For TdT-mediated dUTP nick end labelling (TUNEL) assays, the cells were stained using riboAPO^TM^One-Step TUNEL Apoptosis Kit (Cat.C11026-1, China, RiboBio) according to the manufacturer’s instructions. Images were captured using fluorescence microscope (Leica, Germany).

**FISH and immunofluorescence assay**

Fluorescence in situ hybridization (FISH) assays were performed as following. HUVECs were seeded on the glass coverslip for 4 h until the cells attachment. Then the cells were fixed in RNAase-free 4% PFA and permeabilized in freshly made 0.5% TritonX-100. After rinsed with 2×SSC, the cells were hybridized with 5’digoxin (5’-DIG) and 3’digoxin (3’-DIG) double labeled locked nucleic acid (LNA)-modified HDRACA detection probes (customized by Exiqon) at 53°C overnight in a moist chamber. 5’-DIG labeled LNA-modified hsa-ACTB detection probes (Exiqon) were designed as positive control, and 5’-DIG and 3’-DIG double labeled locked nucleic acid (LNA)-modified Scramble-ISH were designed as negative control. After washing, the cells were incubated at 4 ℃ overnight with an FITC Anti-Digoxigenin antibody (Cat. ab119349, UK, Abcam).

After HUVECs were subjected to FISH, immunofluorescence staining was performed to analyze the co-location of HDRACA and RAIN. Immunofluorescence assays were performed as following. HUVECs were fixed in 4% PFA and permeabilized in freshly made 0.5% TritonX-100. Then, cells were incubated with RAIN Antibody (Cat. 26064-1-AP, USA, Proteintech) overnight at 4 ℃, followed by 30 min incubation with Goat Anti-Rabbit IgG H&L (Alexa Fluor® 555) (Cat. ab150078, UK, Abcam) at RT.

FISH assays with HDRACA detection probes were performed to examine whether HDRACA was sufficiently expressed in the endothelial cells of mice muscle at day 7 post-surgery. After fixed and permeabilized, the frozen sections of adductor muscles were hybridized with the DIG double labeled LNA-modified HDRACA detection probes as described above. Then, the sections were incubated with anti-DIG antibody (Cat. ab76907, UK, Abcam) at 4°C overnight, followed by 1 h incubation with Alexa Fluor 647-conjugated secondary antibody (Cat. ab150131, UK, Abcam). Next, the sections were incubated with CD31 antibody (Cat. ab264486, UK, Abcam) overnight at 4 ℃, followed by incubation with FITC-conjugated secondary antibody (Cat. SA00003-11, USA, Proteintech) for 1 h.

Immunofluorescence staining was performed to evaluate the vascular density of the frozen muscle tissue sections. After fixed and permeabilized, frozen muscle tissue sections were incubated with anti-CD31 antibody (Cat. ab24590, UK, Abcam), Then, the tissue sections were incubated with Alexa Fluor 488-conjugated secondary antibody (Cat. A-11008, USA, Invitrogen). Furtherly, we evaluated the levels of RAIN, Vigilin or PCNA in the endothelial cells of muscle tissue by performing immunofluorescence co-staining for CD31 with RAIN, Vigilin or PCNA using the following primary antibodies: CD31 rat antibody (Cat. ab264486, UK, Abcam) and RAIN rabbit antibody (Cat. 26064-1-AP, USA, Proteintech) or Vigilin rabbit antibody (Cat. 15406-1-AP, USA, Proteintech) or PCNA rabbit antibody (Cat. 13110, USA, Cell Signaling Technology), followed by incubation with FITC-conjugated secondary antibody (Cat. SA00003-11, USA, Proteintech) and Alexa Fluor 647-conjugated secondary antibody (Cat. ab150079, UK, Abcam).

We performed co-staining for HDRACA, KLF5 or PCNA with CD31 to evaluate to their expression levels in the endothelial cells of lower limb arteries. We marked HDRACA by hybridizing with DIG double labeled LNA-modified HDRACA detection probes and incubating with anti-DIG antibody (Cat.ab420, UK, Abcam) and Alexa Fluor 555-conjugated secondary antibody (Cat.150106, UK, Abcam). We marked KLF5 or PCNA by incubating with primary antibodies against KLF5 (Cat.21017-1-AP. USA. Proteintech) or PCNA (Cat.13110. USA. Cell Signaling Technology), followed by incubation with Alexa Fluor 555-conjugated secondary antibody (Cat.150078, UK, Abcam). Then, we marked the endothelial cells by incubating with CD31 antibody (Cat.ab264486, UK, Abcam) and FITC-conjugated secondary antibody (Cat.SA00003-11, USA, Proteintech).

All the cells or sections were counterstained with 4',6-diamidino-2-phenylindole (DAPI) for the nuclei and imaged were obtained using a confocal laser scanning microscope equipped with a core data acquisition system (Nikon Eclipse Ni-E, Japan or LSM780, Zeiss, German).

**ChIP assay**

Chromatin immunoprecipitation (ChIP) assays were performed according to the manufacturer’s instructions of SimpleChIP Plus Enzymatic Chromatin IP Kit (Cat. 9005, USA, Cell Signaling Technology). In brief, HUVECs were crosslinked with 1% formaldehyde. Then, the cells were lysed and sonicated to generate DNA fragments. The chromatin was immunoprecipitated by KLF5 antibody (Cat. 61099, USA, Active Motif) or IgG (Cat. 3900S, USA, Cell Signaling Technology) conjugated to Protein G magnetic beads. The precipitated chromatin DNA were detected by RT-qPCR to quantify the binding of the HDRACA or BECN1 promoter to KLF5 or IgG. The specific primers used to detect KLF5 binding sites were listed in supplementary table S6. The whole-cell lysate and immunoprecipitated proteins of each immunoprecipitation were subjected to immunoblot for KLF5 or IgG.

**Luciferase reporter assay**

Series of pGL4.10 reporter plasmids carrying sequential deletions of the 5′-flanking region of HDRACA and pRL-TK-renilla-luciferase plasmid were transfected into cells. Luciferase activity was assayed 72 h after transfection

We cloned the promoter sequence of HDRACA from -65 to +385 and generated a mutant, then inserted them into the pGL4.10 reporter plasmids. The reporter plasmids carrying wild type or mutated promoter constructs and pRL-TK-renilla-luciferase plasmid were transfected into HUVECs using Lipofectamine^TM^ 3000 Transfection Reagent (Cat. L3000015, USA, Invitrogen). Luciferase activity was assayed 72 h after transfection using Dual-Lucy Assay Kit (Cat.D0010, China, Solarbio) according to the manufacturer’s instructions. The mutation strategy was as follow: wild type: +72GCCCCGCCCC+83; mutant: +72ATTTCTAAAC+83.

The promoter sequence of human PCNA from -2000 to -1 was cloned and inserted into the pGL4.10 reporter plasmids. Then, this reporter plasmids and pRL-TK-renilla-luciferase plasmid were transfected into HUVECs. Luciferase activity was assayed 72 h after transfection.

All the pGL4.10 reporter plasmids and pRL-TK-renilla-luciferase plasmid were transfected using Lipofectamine^TM^ 3000 Transfection Reagent (Cat. L3000015, USA, Invitrogen) according to the manufacturer’s instructions. Luciferase activity was assayed using Dual-Lucy Assay Kit (Cat.D0010, China, Solarbio) according to the manufacturer’s instructions. Firefly luciferase activity was normalized to renilla luciferase activity.

**Cell transduction and overexpression**

KLF5-ΔPY2-overexpressing lentiviruses, FLAG tagged wild type or mutant WWP2-overexpressing lentiviruses, HDRACA-overexpressing lentiviruses and RAIN-overexpressing lentiviruses and FLAG tagged full length or truncated RAIN-overexpressing lentiviruses were designed and purchased from Umine-biology, China. Cells were cultured in 6-well plates until 30%-50% confluent. Then, the cells were infected with lentivirus particles at a multiplicity of infection (MOI) of 5 and continued to culture for at least 72 h before further experiments.

**CCK8 proliferation assays**

Cell counting kit-8 (CCK8) proliferation assays were performed using Cell Counting Kit 8 (Cat. CK04, Japan, Dojindo) according to the manufacturer’s instructions. In brief, 2500 cells/well were seeded in 96-well plate overnight before incubated in ECM containing 0.5% FBS and 100 μg/ml HDL (nHDL or dHDL) or 50 ng/ml VEGF or 1μM r-ApoM-S1P for 24 h. Next, the medium was replaced with 100 μl ECM containing 0.5% FBS and 10% CCK8 solution followed by 3 h incubation. Absorbance was measured using a microplate reader at 450 nm.

**EdU incorporation assays**

5-Ethynyl-2’-deoxyuridine (EdU) incorporation assays were performed using Cell-Light EdU Apollo567 In Vitro Kit (Cat. C10310-1, China, RiboBio) according to the manufacturer’s instructions. In brief, 2500 cells per well were seeded in 96-well plate overnight. Then, the cells were incubated in ECM containing 0.5% FBS and 100 μg/ml HDL (nHDL or dHDL) or 50 ng/ml VEGF or 1 μM r-ApoM-S1P for 24 h. Next, after incubated in the ECM containing 5% FBS and 50 μM EdU solution, the cells were fixed in 4% PFA and permeabilized in freshly made 0.5% Triton X-100. After washed with PBS, the cells were incubated with Apollo^®^567 for 30 min and washed with PBS containing with 0.5% Triton X-100. Finally, cells were counterstained with Hoechst33342 and imaged using fluorescence microscope (Leica, Germany).

**Transwell migration assays**

Transwell migration assays were performed as previously described^10^. In brief, HUVECs were cultured in the ECM with 0.5% FBS containing 100 μg/ml HDL (nHDL or dHDL) or 50 ng/ml VEGF or 1 μM r-ApoM-S1P for 24 h. Then, cell culture inserts with 8.0 μm pore size PET track-etched membranes (Cat. 353097, USA, Falcon) were coated with 0.1% gelatin for 1 h and then blocked with 1% BSA at 37°C for 30min. Next, the cells were detached and resuspended in ECM with 0.5% FBS at a concentration of 1×10^5^ cells/ml. Furthermore, we added the cells to the upper chamber at a density of 5×10^4^ cells/insert. Simultaneously, ECM with 5% FBS was added to the lower compartment. The cells were incubated at 37°C for 4 h. At the end of the incubation, the inserts were washed with PBS, and the cells were fixed with 4% PFA and stained with crystal violet. Cells that had not migrated were scraped off gently with a cotton swab. The migrant cells on the lower surface of the membrane were imaged in five randomly chosen fields using photomicroscope (Leica, Germany).

**Tube Formation assay**

Tube formation assays were performed as previously described^2^. In brief, 24-well culture plates were coated with Matrixgel (REF.354234, USA, Corning), followed by polymerization for 30 min at 37°C. Then, HUVECs were seeded on coated plates at a density of 1×10^5^ cells/well in ECM containing 0.5% FBS and 100 μg/mL HDL (nHDL or dHDL) or 50 ng/ml VEGF or 1 μM r-ApoM-S1P and allowed to incubated for 12 h at 37°C. Pictures were taken using photomicroscope (Leica, Germany) and tube lenght was measured using Image J program (NIH).

**RNA-pull-down**

RNA probes were synthesized using MAXIscript® Kit (Cat. AM1314M, USA, Thermo Fisher Scientific) according to manufacturer’s instructions. The RNA pull down assays were performed using the Pierce™ Magnetic RNA-Protein Pull-Down Kit (Cat. 20164, USA, Thermo Fisher Scientific) following the instructions of the manufacturer. In brief, RNA probes were biotin-labeled using the included Thermo Scientific Pierce RNA 3´ Desthiobiotinylation Kit (Cat. 20163, USA, Thermo Fisher Scientific). Then, biotinylated RNAs in RNA structure buffer were heated at 95°C for 2 min, cooled on ice for 3 min, and left at RT for 30 min to form a proper secondary structure. Streptavidin magnetic beads were mixed with the folded RNAs and incubated at RT for 30 min, followed by gently washed. RNA-bead mixtures were incubated with the cell lysate at RT overnight. After the beads were collected and washed, the RNA-binding protein complexes were eluted and then denatured in boiled water for 10 min. The denatured proteins were resolved by SDS-PAGE followed by silver staining. Finally, mass spectrometry or immunoblot analysis were performed to identify retrieved proteins.

**RIP assay**

RNA-immunoprecipitation (RIP) assays were performed using Magna RIP™ RNA-Binding Protein Immunoprecipitation Kit (Cat. 17700, USA, Millipore Sigma) according to manufacturer’s instructions. In brief, Protein A/G magnetic beads were washed twice, collected, and resuspended. After antibody or IgG were mixed with the beads, followed by incubation with rotation for 30 min at RT. Then, cell lysates were added to beads-antibody complex and incubated at 4°C overnight. Next, immunoprecipitated products were collected, washed, and treated with proteinase K. Finally, total RNAs were extracted from the immunoprecipitated samples and subjected to RT-qPCR analysis. The antibodies used in this study: RASIP1 Antibody (Cat. 17971-1-AP, USA, Proteintech), Vigilin antibody (Cat. sc-271523, USA, Santa Cruz Biotechnology).

**RNA stability assay**

HUVECs were treated with 10 μM α-amanitin (Cat. A4548, USA, APExBIO) to block mRNA transcription. Then, the cells were collected at the indicated time points. The total RNA was extracted using Trizol Reagent (Cat. T9424, USA, Sigma-Aldrich) and further analyzed by RT-qPCR.

**Polysome gradient assays**

We performed polysome gradient assays as described previously with modifications^11, 12^. In brief, HUVECs were incubated with 100 μg/ml cycloheximide (Cat. GC17198, USA, Glpbio) for 10 min. Then, the cells were lysed in the buffer containing 20 mM Tris-HCl (pH 7.5), 100 mM KCl, 5 mM MgCl_2_, 0.5% Nonidet P-40, 100 µg/ml cycloheximide, 1×proteinase inhibitor and 1:1,000 dilution of RiboLock RNase inhibitor (Cat. EO0381, USA, Thermo Fisher Scientific). After centrifugation, the supernatant were loaded on to 10–50% sucrose gradients, and the gradients were centrifuged at 260,343 g at 4°C for 2 h. Next, RNAs were extracted with an equal volume of Trizol:chloroform (5:1), Then RNA precipitation was performed with equal isopropanol containing 30 μg/ml glycoblue (Cat. AM9515, USA, Thermo Fisher Scientific) overnight at -20°C, and pellet was washed once with ice-cold 70% ethanol. Extracted RNAs were subjected to reverse transcription, and RT-qPCR was performed to analyze the distribution of PCNA mRNA.

**RNA antisense purification**

RNA antisense purification (RAP) assays were performed as previously described with modification^13^. Biotin-labeled probes were denatured at 85 ℃ for 3 min and mixed with streptavidin beads in 1×RAP hybridization buffer, followed by 30 min incubation at RT. Next, HUVECs were crosslinked in a fixing solution containing 1% formaldehyde at RT for 10 min, followed by neutralized with 0.1g glycine per 10 mL fixation solution at RT for 5 min. The crosslinked cells were further lysed in pre-cool lysis buffer containing protease inhibitor and RNase inhibitor, and DNA was removed using DNase. After centrifugation, the supernatant was diluted with 2×RAP hybridization buffer and denatured at 65 ℃ for 10 min. Then, the denatured samples were mixed with beads-probe complex, followed by 30 min hybridization at 45 ℃, 5 min denaturation at 50 ℃ and 3 h hybridization at 45 ℃. Next, the beads were collected, washed, and eluted, followed by 1 h digestion with proteinase K. Finally, RNAs were purified and subjected to RT-qPCR analysis. The probes used in this study were listed in the supplementary table S6.

**Measurements of vascular growth in vivo**

Adenovirus vector carrying HDRACA (AdV-HDRACA) and control adenovirus vector (AdV-Ctrl) were designed and purchased from Umine-bio, China. Both adenovirus vectors carried mCherry proteins coding gene. Animal experiments were approved by the Ethics Review Board of the First Affiliated Hospital, Sun Yat-sen University (Approval number: [2020]019).

A model of lower limb ischemia was produced in C57BL/6 mice to evaluate the regulatory function of angiogenesis by HDRACA. 5-8 week-old C57BL/6 mice were obtained from Laboratory Animal Center of Sun Yat-sen University (Guangzhou, China). Mice were randomly (simple randomization) assigned to three groups. Investigator was unaware of allocations. After anesthesia of mice, the femoral artery was dissected and ligated at two positions. One was proximal to the caudal femoral artery and the other was distal to it and proximal to popliteal artery. AdV-HDRACA, AdV-Ctrl or normal saline (NS) were injected at two sites of the adductor muscle and at two sites into gastrocnemius muscle. The injections were administered twice. The first time was at the time of operation, and the second time was at day 7 post-surgery. The adenovirus dosage is 2×10^8^ plaque-forming units at a time. The feet of the mice were imaged to measure the blood flow using laser Doppler imaging system (PSI-ZR, Perimed, Järfälla, Sweden) at day 1 pre-surgery and at day 0, 7, 14 post-surgery^14^. Mice were euthanized at day 14 and the hindlimb vasculature were flushed with heparinized normal saline and then fixed with 2% PFA. MICROFIL contrast agent (Cat. MV-122, USA, Flow Tech Inc) was injected to perfuse the hindlimb vasculature according to manufacturer’s instructions, followed by 2 weeks’ decalcification with 10% EDTA. Hindlimb vascular network morphology was imaged and analyzed with a micro-computed tomography (Micro-CT) imaging system (Inveon PET/CT, Siemens, Munich, Germany) as previously described^2^.

In addition, adductor muscles of mice sacrificed at day 7 or 14 post-surgery were harvested. For evaluating transfection efficiency with adenovirus, the muscles acquired at day 7 post-surgery were subjected to detect the distribution of adenovirus vector expression in the injured limb muscle via analyzing the co-location of mCherry fluorescence and CD31 staining. For detecting the expression effect of HDRACA, the muscles acquired at day 7 post-surgery were subjected to detect the expression of HDRACA by RT-PCR and FISH assays. For RT-PCR assays, extracted RNA was reversely transcripted into cDNA, amplified with HDRACA or Gapdh specific primers and further subjected to DNA agarose gel electrophoresis. For immunofluorescence assays, endothelial cells in tissue sections of adductor muscles were stained using CD31 antibody (Cat. ab24590, UK, Abcam). FISH of HDRACA was performed after immunofluorescence of CD31 to further confirm the expression of HDRACA in endothelial cells. The muscles harvested at day 14 post-surgery were subjected to immunofluorescence of CD31 to compare capillaries densities in adductor muscles of different groups. Immunofluorescence co-staining for RAIN, Vigilin or PCNA with CD31 was performed to evaluate these indicators levels in in the endothelial cells of adductor muscle.

Furtherly, we performed Matrigel plug assay in vivo to examine the role of HDRACA in angiogenesis as previously described^15, 16^. 5-8 week-old C57BL/6 mice were obtained from Laboratory Animal Center of Sun Yat-sen University (Guangzhou, China). Mice were randomly (simple randomization) assigned to three groups. Investigator was unaware of allocations. AdV-Ctrl, AdV-HDRACA or NS were mixed with Matrigel solution (Cat. 354262, USA, Corning) and 30 U/ml heparin on ice (2×108 plaque-forming units of virus/500 μl Matrigel). After anesthesia, Matrigels mixed with NS or AdV-Ctrl or AdV-HDRACA were subcutaneously injected into the abdomen of mice. Mice were euthanized at day 7 after injection. The Matrigel plugs were carefully taken out and remove connective and adipose tissues surrounding. After photographing the gross morphology, the plugs were destined for paraffin sectioning.

Hematoxylin-eosin (HE) staining and CD31 immunohistochemistry (IHC) were performed on paraffin sectioning. For HE staining, paraffin sections were dewaxed and rehydrated. After staining with hematoxylin for 5 min，the sections were continued to stain with eosin for 5 min. Then the sections were dehydrated with ethanol and treated with xylene. Finally, the sections were visualized and imaged under a photomicroscope (Nikon, Tokyo). For IHC staining, the paraffin sections were deparaffinized, hydrated, and boiled in sodium citrate buffer (pH 6.0) to facilitate antigen retrieval. After blocking with 5% normal goat serum, the slides were incubated with the CD31 antibody (Cat. GB113151, China, servicebio) at 4 ℃ overnight, followed by 50 min incubation with HRP-labeled secondary antibody (Cat. GB23303, China Servicebio). The color was developed with diaminobezidin (DAB). The sections were counterstained with hematoxylin, dehydrated with ethanol and treated with xylene. Finally, the images were taken using a photomicroscope (Nikon, Tokyo).

**Statistical analysis**

Data are presented as the mean ± SD. Statistical analyses were performed using SPSSv.13.0 or Prism GraphPad 9.0. The differences among the test groups were determined with one-way ANOVA followed by Tukey’s test or Kruskal-Wallis test for more than two groups or with Student T-test or Mann-Whitney test for two groups. p<0.05 was considered statistically significant. Statistical details and methods used are indicated in the figure legends, text or methods.

**Graphic Illustration**

The graphical representation of 3′ and 5′ RACE assays (Figure 1e), schematic diagram of wild type or mutant FLAG tagged WWP2 (Supplementary Figure 5d) and schematic diagram of FLAG tagged RAIN truncated mutants (Supplementary Figure 11a) were made with the Illustrator for Biological Sequences (IBS) software^17^. The schematic graphic of the ischemia model (Figure 7a), graphical illustration of HDL-HDRACA regulatory mechanism (Figure 8d) and schematic graphic of Matrigel plug assay (Supplementary Figure 14a) were created with BioRender.com. The schematic diagram of ChIP-seq signals and KLF5 binding motif (Figure 2e) were made with Integrative Genomics Viewer (IGV) software^18^. The secondary structures of HDRACA and HDRACA^304-358^ (Figure 4e) were predicted by Mfold software^19^.

**Reference**

1. Liu, X. *et al.* High density lipoprotein from coronary artery disease patients caused abnormal expression of long non-coding RNAs in vascular endothelial cells. *Biochem Biophys Res Commun.* **487**, 552-559 (2017).

2. Li, H.M. *et al.* Angiogenic and Antiangiogenic mechanisms of high density lipoprotein from healthy subjects and coronary artery diseases patients. *Redox Biol.* **36**, 101642 (2020).

3. Ahnström, J., Faber, K., Axler, O. & Dahlbäck, B. Hydrophobic ligand binding properties of the human lipocalin apolipoprotein M. *J Lipid Res.* **48**, 1754-1762 (2007).

4. Sattler, K. *et al.* Defects of High-Density Lipoproteins in Coronary Artery Disease Caused by Low Sphingosine-1-Phosphate Content: Correction by Sphingosine-1-Phosphate-Loading. *J Am Coll Cardiol.* **66**, 1470-1485 (2015).

5. Chirinos, J.A. *et al.* Reduced Apolipoprotein M and Adverse Outcomes Across the Spectrum of Human Heart Failure. *Circulation.* **141**, 1463-1476 (2020).

6. Frej, C. *et al.* Quantification of sphingosine 1-phosphate by validated LC-MS/MS method revealing strong correlation with apolipoprotein M in plasma but not in serum due to platelet activation during blood coagulation. *Anal Bioanal Chem.* **407**, 8533-8542 (2015).

7. Bosteen, M.H., Dahlbäck, B., Nielsen, L.B. & Christoffersen, C. Protein unfolding allows use of commercial antibodies in an apolipoprotein M sandwich ELISA. *J Lipid Res.* **56**, 754-759 (2015).

8. Ou, Z.J. *et al.* 25-Hydroxycholesterol impairs endothelial function and vasodilation by uncoupling and inhibiting endothelial nitric oxide synthase. *Am J Physiol Endocrinol Metab.* **311**, E781-e790 (2016).

9. Yuan, H.X. *et al.* Endothelial extracellular vesicles induce acute lung injury via follistatin-like protein 1. *Sci China Life Sci.* http://dx.doi.org/10.1007/s11427-022-2328-x (2023).

10. Zhang, Y. *et al.* Secreted monocytic miR-150 enhances targeted endothelial cell migration. *Mol Cell.* **39**, 133-144 (2010).

11. Panda, A.C., Martindale, J.L. & Gorospe, M. Polysome Fractionation to Analyze mRNA Distribution Profiles. *Bio Protoc.* **7** (2017).

12. Xu, H. *et al.* Inducible degradation of lncRNA Sros1 promotes IFN-γ-mediated activation of innate immune responses by stabilizing Stat1 mRNA. *Nat Immunol.* **20**, 1621-1630 (2019).

13. Engreitz, J.M. *et al.* RNA-RNA interactions enable specific targeting of noncoding RNAs to nascent Pre-mRNAs and chromatin sites. *Cell.* **159**, 188-199 (2014).

14. Mathiyalagan, P. *et al.* Angiogenic Mechanisms of Human CD34(+) Stem Cell Exosomes in the Repair of Ischemic Hindlimb. *Circ Res.* **120**, 1466-1476 (2017).

15. Meng, S. *et al.* Reservoir of Fibroblasts Promotes Recovery From Limb Ischemia. *Circulation.* **142**, 1647-1662 (2020).

16. Han, B. *et al.* Exosomal EPHA2 derived from highly metastatic breast cancer cells promotes angiogenesis by activating the AMPK signaling pathway through Ephrin A1-EPHA2 forward signaling. *Theranostics.* **12**, 4127-4146 (2022).

17. Liu, W. *et al.* IBS: an illustrator for the presentation and visualization of biological sequences. *Bioinformatics.* **31**, 3359-3361 (2015).

18. Robinson, J.T., Thorvaldsdottir, H., Turner, D. & Mesirov, J.P. igv.js: an embeddable JavaScript implementation of the Integrative Genomics Viewer (IGV). *Bioinformatics.* **39** (2023).

19. Zuker, M. Mfold web server for nucleic acid folding and hybridization prediction. *Nucleic Acids Res.* **31**, 3406-3415 (2003).


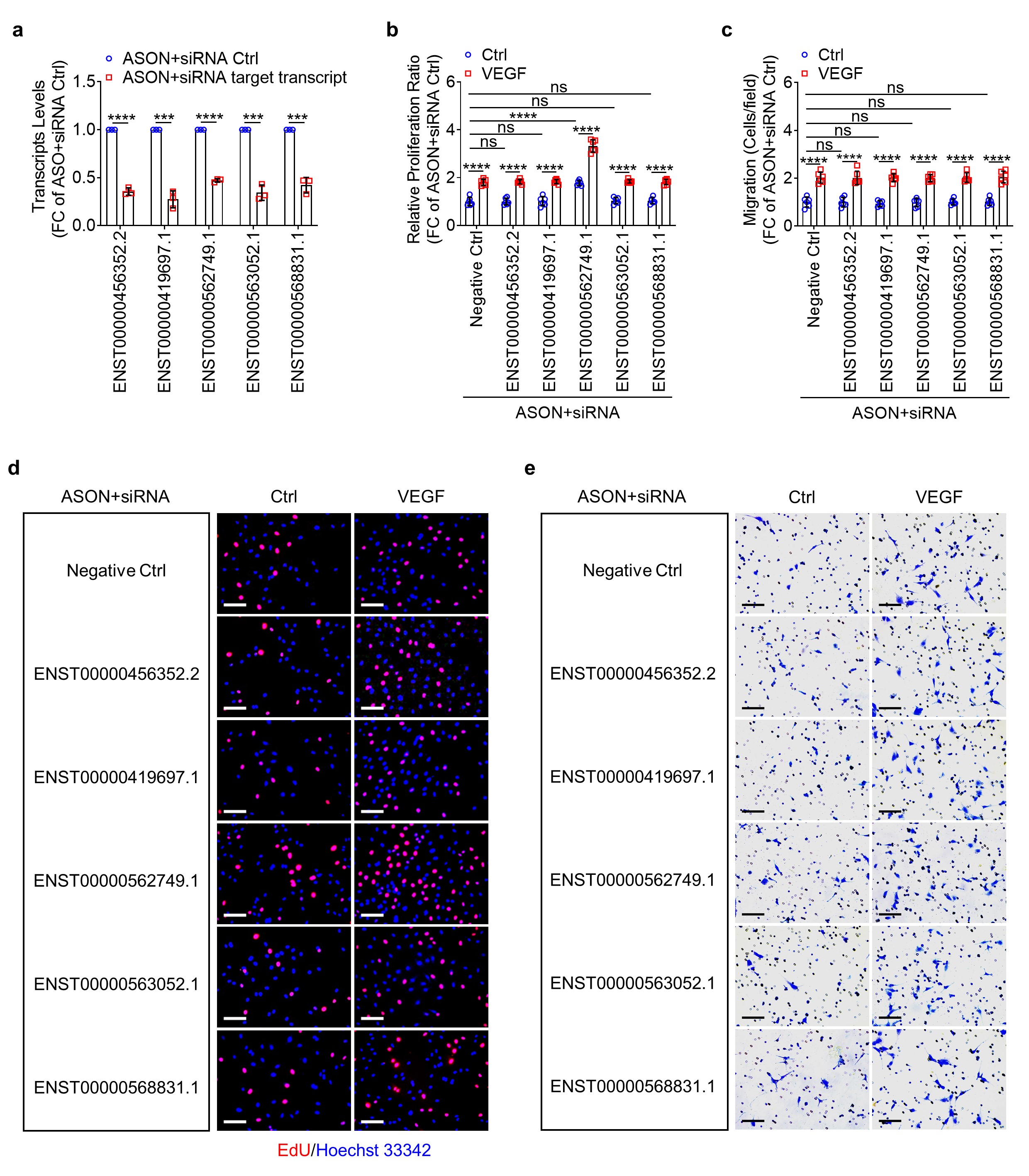


**Supplementary Figure S1. Effects of five candidate transcripts on angiogenesis**

**a.** The 5 candidate transcripts expression in human umbilical vein endothelial cells (HUVECs) transfected with antisense oligonucleotides (ASONs) and small interfering RNAs (siRNAs) respectively were determined by real-time quantitative PCR (RT-qPCR). **b, d.** The quantification (**b**) and representative images (**d**) of 5-Ethynyl-2’-deoxyuridine (EdU) incorporation assay after knocking down 5 candidate transcripts using ASONs (66 nM) and siRNAs (33 nM) in HUVECs. The proliferative HUVECs were labeled with EdU (red) and the nuclei were stained with Hoechst 33342 (blue). Scale bars, 100 μm. **c, e.** The quantification (**c**) and representative images (**e**) of transwell migration assay after knocking down 5 candidate transcripts using ASONs (66 nM) and siRNAs (33 nM) in HUVECs. Scale bars, 100 μm. For **a**, n=3. For **b-e**, n=6. *******p<0.001; ********p<0.0001; ns, not significant.

**Supplementary Figure S2. Identification and characterization of HDRACA**


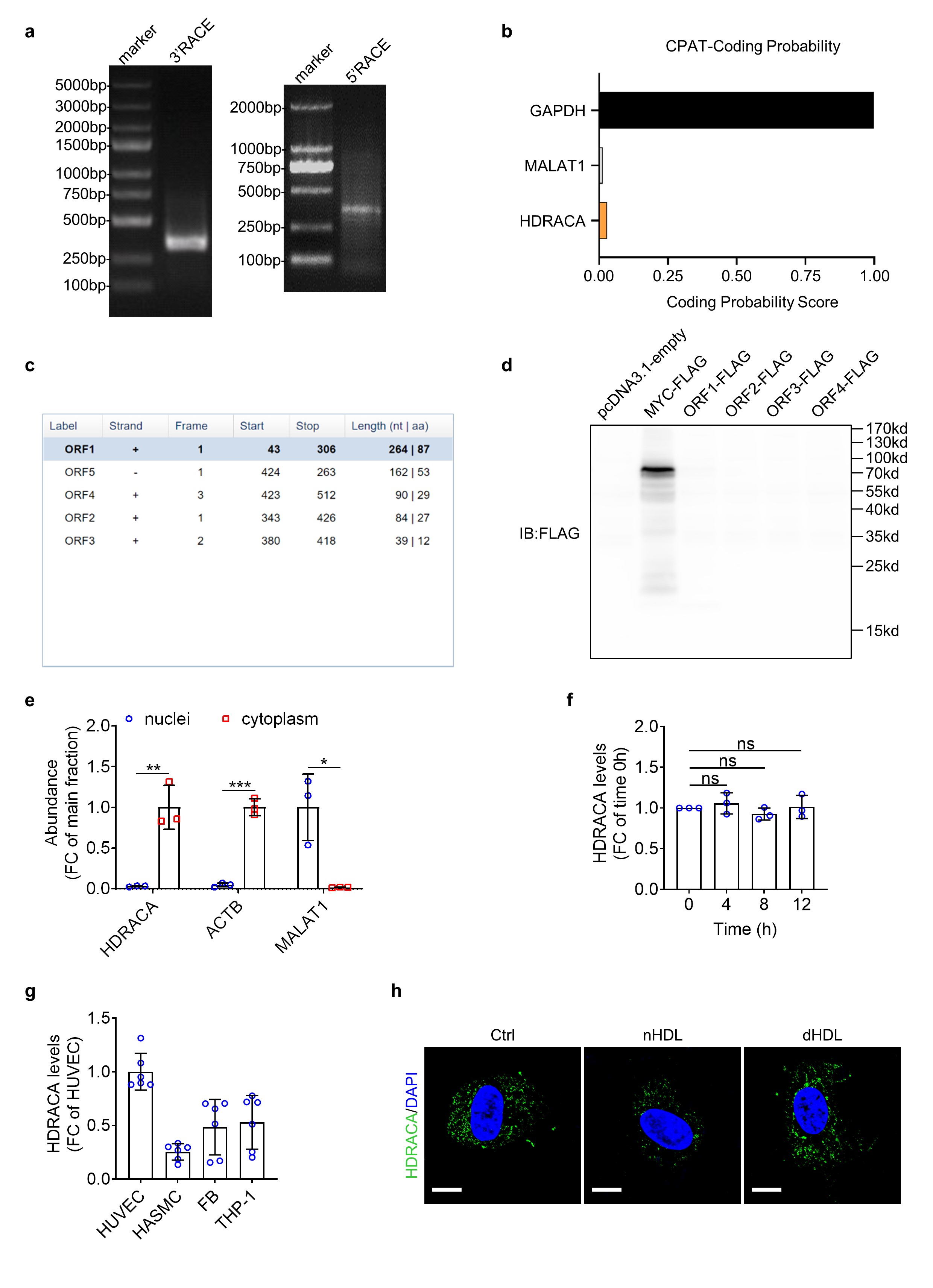


**a.** Reverse transcription PCR (RT-PCR) showed the amplification products of 5ʹ and 3ʹ rapid-amplification of cDNA ends (RACE) of ENST00000562749.1. **b.** Coding potential assessment tool (CPAT) predicted for coding potentials of HDRACA. **c.** NCBI ORFfinder predicted for the open reading frames (ORFs) of HDRACA. **d.** The predicted ORF sequences of HDRACA were cloned upstream of 3xFlag-Tag cassette, transfected in HEK-293T cells, and immunoblotted for Flag tag. **e.** Subcellular abundance of HDRACA in human umbilical vein endothelial cells (HUVECs) was determined by RT-qPCR. **f.** The levels of HDRACA during different time points of tube formation assays were determined by RT-qPCR. **g.** Droplet digital PCR (ddPCR) was used to determine HDRACA levels in different types of vascular cell, including HUVEC, human aortic smooth muscle cell (HASMC), human aortic fibroblast (FB) and THP-1 macrophage (THP-1). **h.** Fluorescence in situ hybridization (FISH) assays confirmed the expression of HDRACA in HUVECs treated with normal HDL (nHDL) or dysfunctional HDL (dHDL). Representative images of HDRACA (green) are shown. The nuclei are stained with DAPI (blue). Scale bars, 10 μm. Data are presented as the mean ± SD. For **d-f**, n=3. For **g** and **h**, n=6. *****p<0.05; ******p<0.01; *******p<0.001; ns, not significant.

**Supplementary Figure S3. HDL bound S1P interacts with S1P1 to regulate HDRACA levels in the endothelial cells**


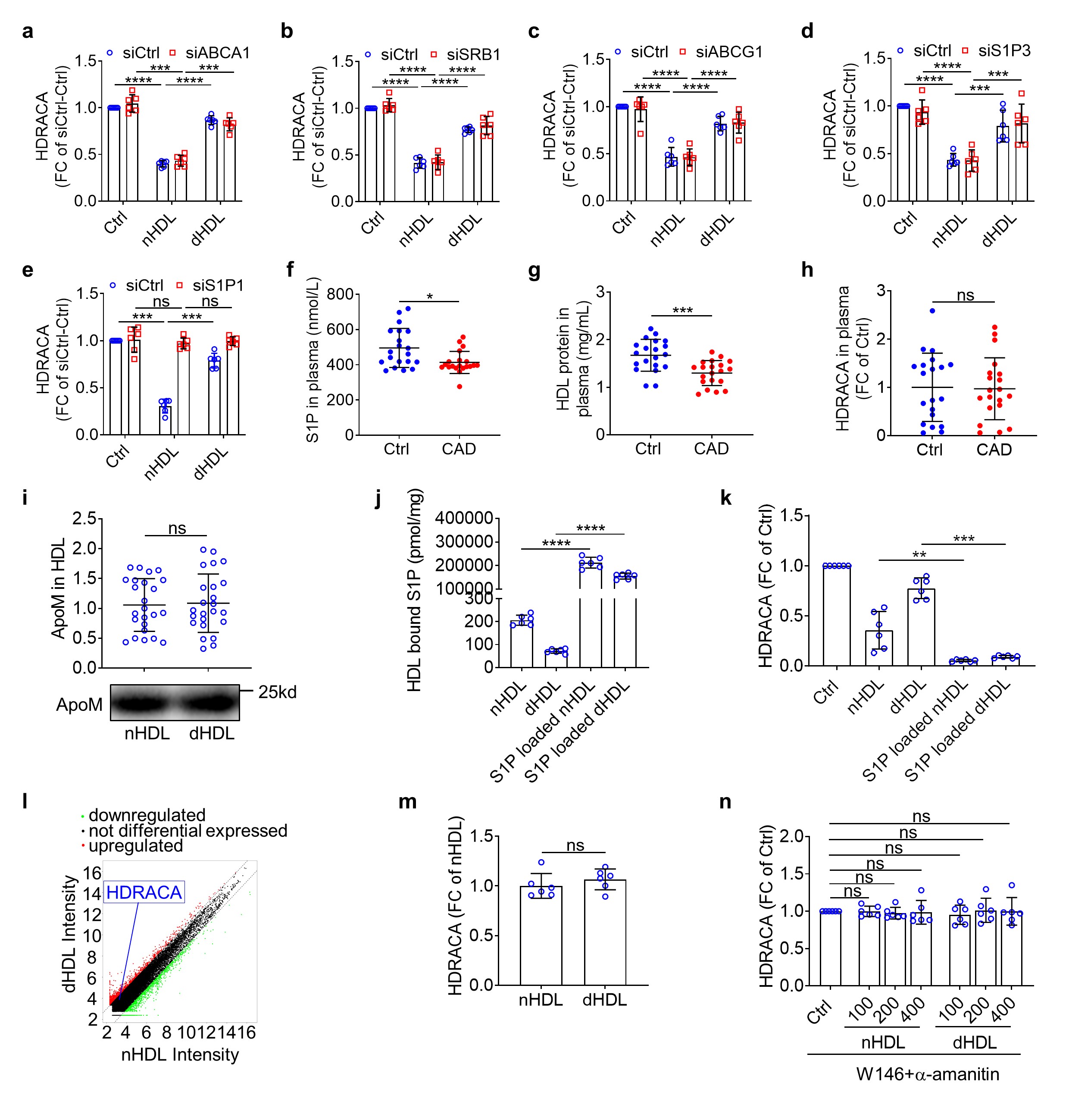


**a-e.** RT-qPCR assays showed the mRNA levels of HDRACA in human umbilical vein endothelial cells (HUVECs) treated with nHDL or dHDL after transfected with siRNAs targeting HDL receptors ATP-binding cassette transporter A1 (ABCA1) (**a**), scavenger receptor class B type 1 (SRB1) (**b**), ATP-binding cassette transporter G1 (ABCG1) (**c**), sphingosine-1-phosphate receptor 3 (S1P3) (**d**) and sphingosine-1-phosphate receptor 1 (S1P1) (**e**). **f-h.** Levels of S1P (**f**), HDL protein (**g**) and HDRACA (**h**) in the plasma of 20 patients with coronary artery disease (CAD) and 20 healthy individuals were measured. **i.** Immunoblotting for the protein levels of apolipoprotein M (ApoM) in nHDL (n=24) and dHDL (n=24). The representative plots (down) and quantification (up) are shown. **j.** S1P concentration on native and S1P loaded nHDL and dHDL were determined by liquid chromatography-tandem mass spectrometry (LC-MS/MS). **k.** RT-qPCR assays confirmed the expression of HDRACA in HUVECs treated with native or S1P loaded nHDL and dHDL. **l.** Scatter plot of nHDL and dHDL bound long noncoding RNAs (lncRNAs). **m.** HDRACA levels in nHDL and dHDL were determined by RT-qPCR assays. **n.** HDRACA levels in HUVECs treated with increasing concentrations of nHDL and dHDL (from 100 μg/ml to 400 μg/ml) after incubated with W146 (10 μM) and α-amanitin (10 μM) were determined by RT-qPCR assays. Data are presented as the mean ± SD. For **a-e**, **j**, **k**, **m**, **n**, n=6. *****p<0.05; ******p<0.01; *******p<0.001; ********p<0.0001; ns, not significant.

**Supplementary Figure S4. nHDL, dHDL and r-ApoM-S1P differently regulate KLF5 levels in the endothelial cells by interacting with S1P1**


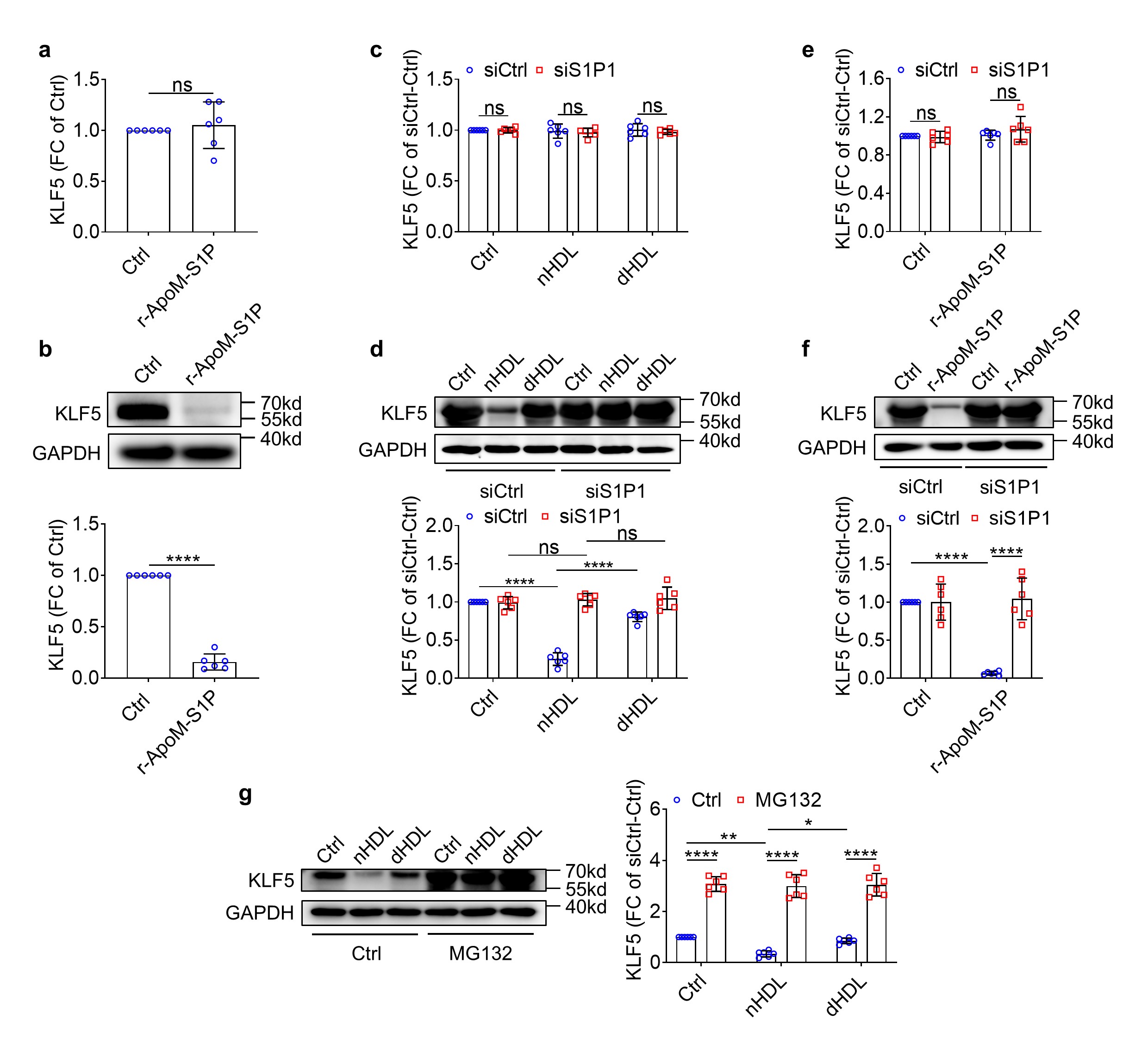


**a.** The mRNA levels of krueppel-like factor 5 **(**KLF5) in human umbilical vein endothelial cells (HUVECs) treated with recombinant human ApoM-bound sphingosine-1-phosphate (r-ApoM-S1P) (1 μM) were determined by RT-qPCR assays. **b.** Immunoblotting for KLF5 in HUVECs treated with r-ApoM-S1P (1 μM). The representative plots (up) and quantification (down) are shown. **c.** RT-qPCR assays showed the mRNA levels of KLF5 in HUVECs treated with nHDL or dHDL after transfected with negative control siRNA or S1P receptor 1 (S1P1)-siRNA. **d.** Immunoblotting for the expression of KLF5 in HUVECs treated with nHDL or dHDL after transfected with negative control siRNA or S1P1-siRNA. The representative plots (up) and quantification (down) are shown. **e.** RT-qPCR assays confirmed the mRNA levels of KLF5 in HUVECs treated with r-ApoM-S1P (1 μM) after transfected with negative control siRNA or S1P1-siRNA. **f.** Immunoblotting for the expression of KLF5 in HUVECs treated with r-ApoM-S1P (1 μM) after transfected with negative control siRNA or S1P1-siRNA. The representative plots (up) and quantification (down) are shown. **g.** Immunoblotting for the expression of KLF5 in HUVECs treated with nHDL or dHDL and with or without the proteasome inhibitor MG132 (10 μM). The representative plots (left) and quantification (right) are shown. Data are presented as the mean ± SD. For all the experiments, n=6. *****p<0.05; ******p<0.01; ********p<0.0001; ns, not significant.

**Supplementary Figure S5. r-ApoM-S1P activates WWP2 to promote the ubiquitination and degradation of KLF5**


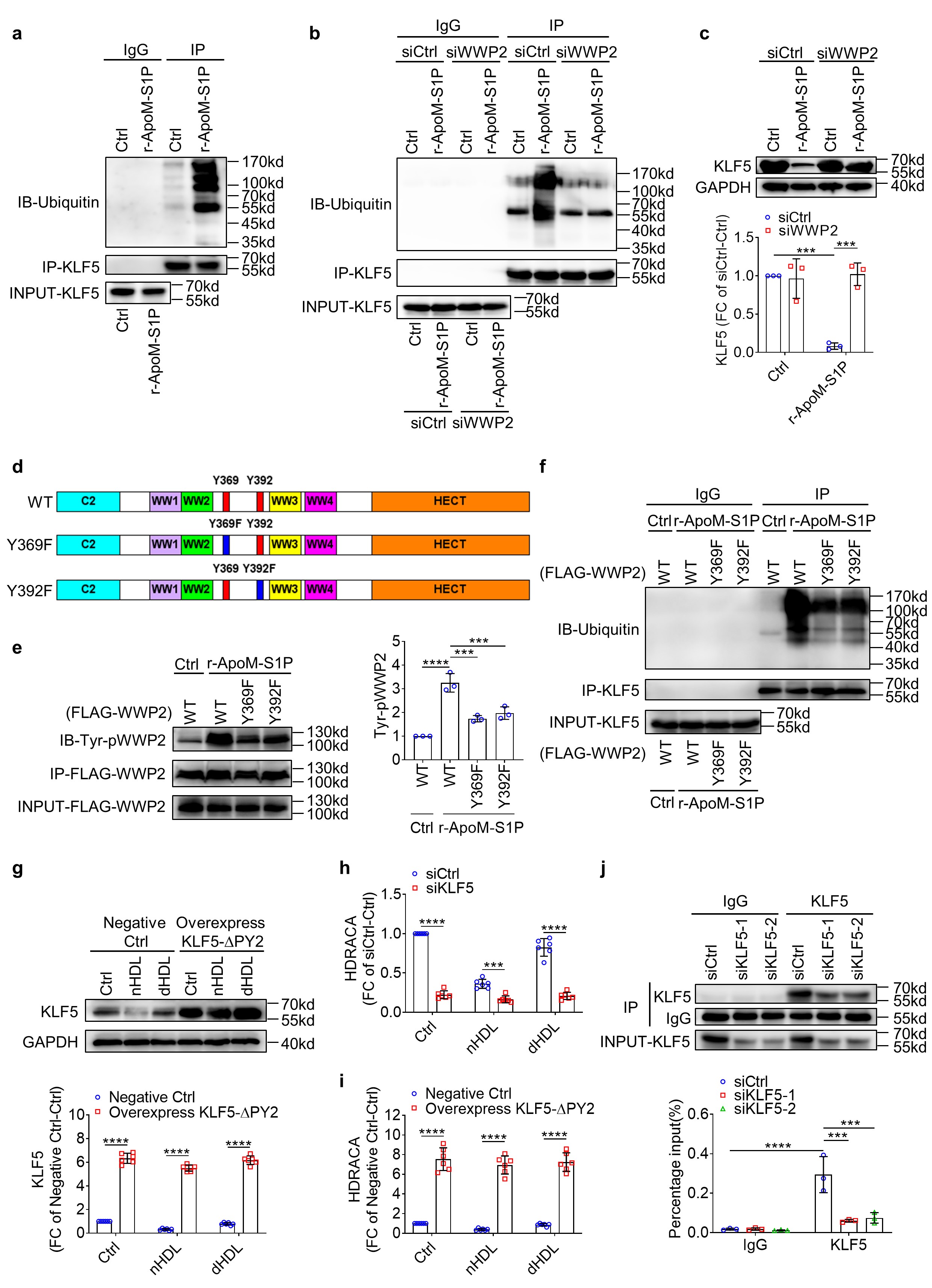


**a.** Krueppel-like factor 5 (KLF5) ubiquitination in human umbilical vein endothelial cells (HUVECs) treated with recombinant human apolipoprotein M-bound sphingosine-1-phosphate (r-ApoM-S1P) (1 μM) was assayed by immunoprecipitation (IP) and immunoblotting (IB). MG132 (10 μM) was added to inhibit KLF5 degradation. **b.** KLF5 ubiquitination in HUVECs treated with r-ApoM-S1P (1 μM) after transfected with negative control siRNA or WW domain-containing E3 ubiquitin protein ligase 2 (WWP2)-siRNA was assayed by immunoprecipitation (IP) and immunoblotting (IB). MG132 (10 μM) was added to inhibit KLF5 degradation. **c.** Immunoblotting for the expression of KLF5 in HUVECs treated with r-ApoM-S1P (1 μM) after knocking down WWP2. The representative plots (up) and quantification (down) are shown. **d.** Schematic diagram showed that either tyrosine at 369 (Y369) or 392 (Y392) sites of FLAG tagged WWP2 proteins were mutated to phenylalanine (Y369F or Y392F). **e.** Immunoblotting detected the effect of r-ApoM-S1P (1 μM) on FLAG tagged WWP2 tyrosine phosphorylation in HUVECs with or without Y369F or Y392F. The representative plots (left) and quantification (right) are shown. **f.** KLF5 ubiquitination in HUVECs treated with r-ApoM-S1P (1 μM) after transducing FLAG tagged wild type or tyrosine mutations of WWP2 were detected by Immunoprecipitation (IP) and immunoblotting (IB). MG132 (10 μM) was added to inhibit KLF5 degradation. **g.** Immunoblotting for the expression of KLF5 in HUVECs treated with nHDL or dHDL after overexpressing KLF5 of PY2 deletion (KLF5-ΔPY2). The representative plots (up) and quantification (down) are shown. **h.** RT-qPCR assays showed the expression of HDRACA in HUVECs treated with nHDL or dHDL after transfected with negative control siRNA or KLF5-siRNA. **i.** The expression of HDRACA in HUVECs treated with nHDL or dHDL after transducing with negative control lentiviruses or KLF5-ΔPY2-overexpressing lentiviruses was determined by RT-qPCR assays. **j.** ChIP-RT-qPCR analysis (down) for KLF5 binding to the HDRACA promoter in HUVECs after knocking down KLF5. Normalized data are shown as percentages of the input controls. The whole-cell lysate (INPUT) and immunoprecipitated proteins (IP) of each immunoprecipitation were analyzed by immunoblotting (up) for KLF5 or IgG. Data are presented as the mean ± SD. For **a-f** and **j**, n=3. For **g-i**, n=6. *******p<0.001; ********p<0.0001.

**Supplementary Figure S6. HDRACA has the greatest effect on the cell cycle of endothelial cells, but has no effect on apoptosis**


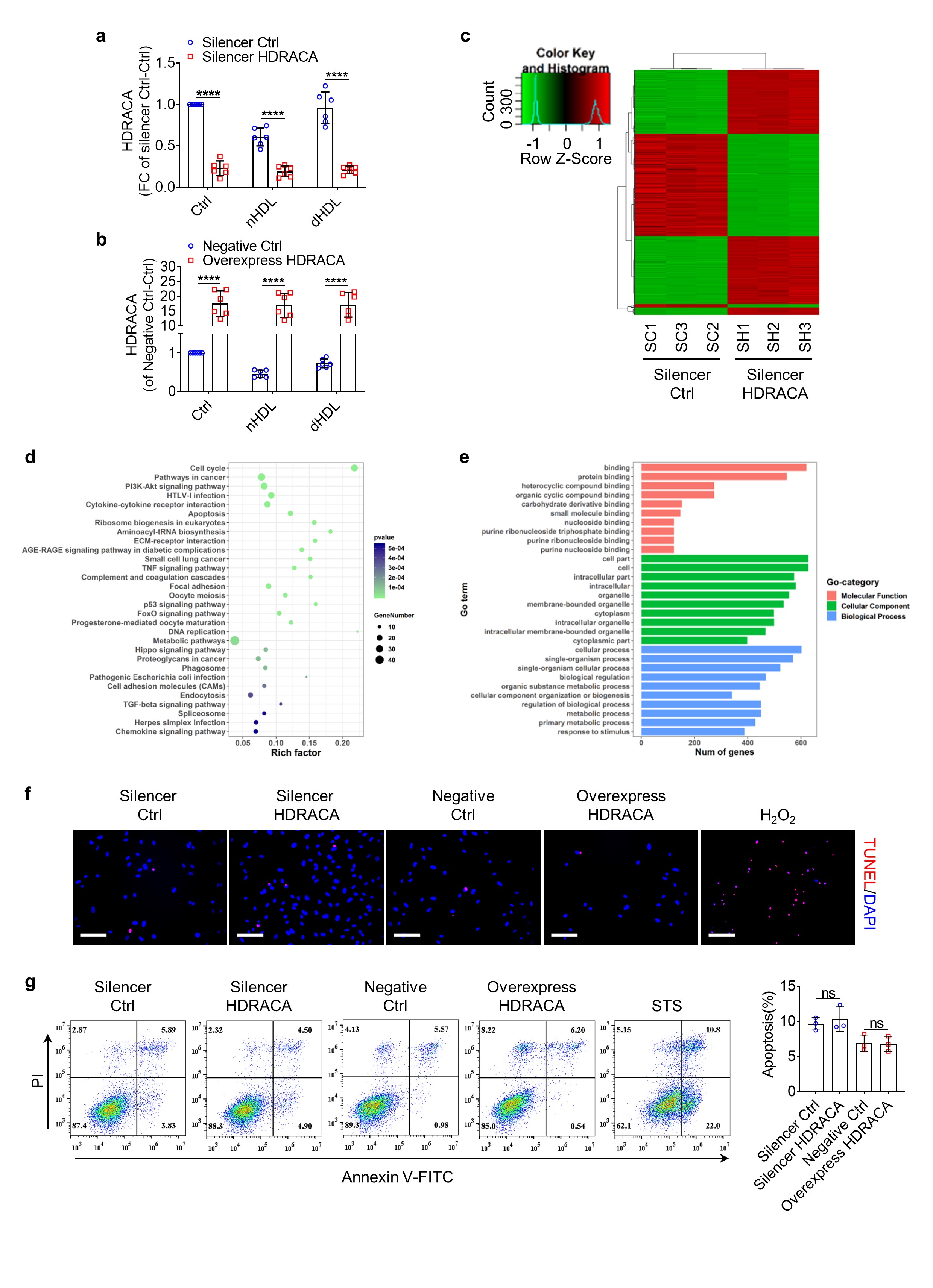


**a.** RT-qPCR assays showed the expression of HDRACA in human umbilical vein endothelial cells (HUVECs) treated with nHDL or dHDL after transfected with Negative Control Smart Silencer (Silencer Ctrl) or HDRACA lncRNA Smart Silencer (Silencer HDRACA). **b.** The expression of HDRACA in HUVECs treated with nHDL or dHDL after transfected with negative control lentiviruses or HDRACA-overexpressing lentiviruses were determined by RT-qPCR assays. **c-e.** Sequencing heatmap (**c**), KEGG pathway analysis (**d**), and GO enrichment analysis (**e**) of differentially expressed genes in HUVECs transfected with Silencer Ctrl (n=3) versus Silencer HDRACA (n=3). **f.** Representative images of TdT-mediated dUTP nick end labelling (TUNEL) in HUVECs after silencing (up) or overexpressing (down) HDRACA. HUVECs treated with H_2_O_2_ were used as a positive control. The TUNEL positive cells (red) represent apoptotic HUVECs and the nuclei were stained with 4',6-diamidino-2-phenylindole (DAPI) (blue). Scale bars, 100 μm. **g.** Flow cytometry analysis for Annexin V-FITC and propidium iodide (PI) staining in HUVECs after silencing or overexpressing HDRACA. The HUVECs treated with 1 μM of staurosporine (STS) were used as a positive control. Representative plots (left) and quantification (right) are shown. Data are presented as the mean ± SD. For **a** and **b**, n=6. For **f** and **g**, n=3. ********p<0.0001; ns, not significant.

**Supplementary Figure S7. nHDL, dHDL and r-ApoM-bound S1P differently regulate HDRACA levels to affect proliferation and tube formation but not migration in endothelial cells**


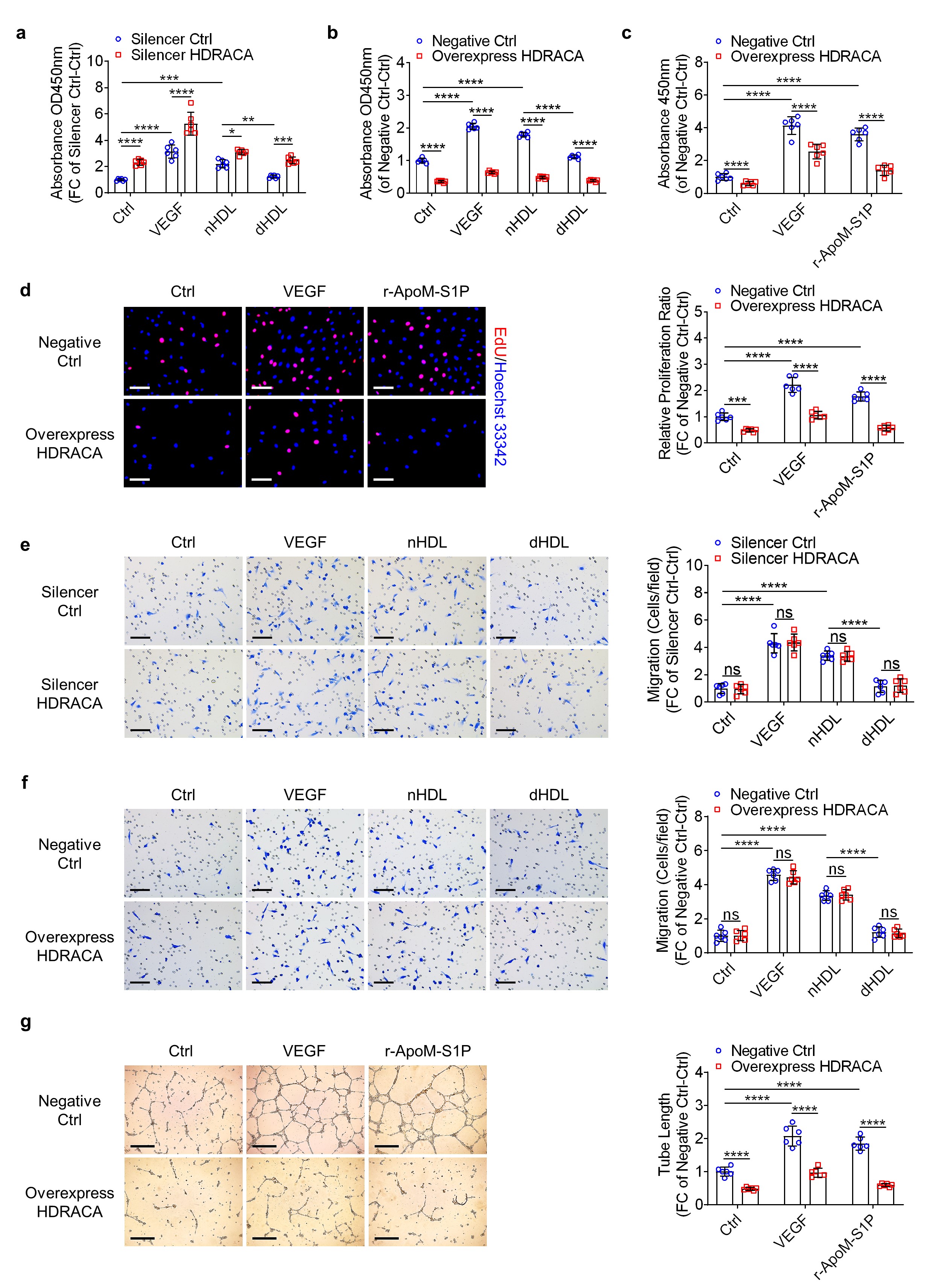


**a, b.** Cell counting kit-8 (CCK8) proliferation assays indicated the proliferation of human umbilical vein endothelial cells (HUVECs) treated vascular endothelial growth factor (VEGF) or nHDL or dHDL after silencing (**a**) or overexpressing (**b**) HDRACA. **c.** HUVECs proliferation treated recombinant human apolipoprotein M-bound sphingosine-1-phosphate (r-ApoM-S1P) (1 μM) after transfected with negative control lentiviruses or HDRACA-overexpressing lentiviruses were determined by CCK8 proliferation assays. **d.** The representative images (left) and quantification (right) of 5-Ethynyl-2’-deoxyuridine (EdU) incorporation assay in HUVECs treated with r-ApoM-S1P (1 μM) after transfected with negative control lentiviruses or HDRACA-overexpressing lentiviruses. The proliferative HUVECs were labeled with EdU (red) and the nuclei were stained with Hoechst 33342 (blue). Scale bars, 100 μm. **e, f.** The representative images (left) and quantification (right) of transwell migration assay in HUVECs treated with VEGF or nHDL or dHDL after silencing (**e**) or overexpressing (**f**) HDRACA. Scale bars, 100 μm. **g.** The representative images (left) and quantification (right) of HUVECs tube formation assay treated with r-ApoM-S1P (1 μM) after transfected with negative control lentiviruses or HDRACA-overexpressing lentiviruses. Scale bars, 500 μm. Data are presented as the mean ± SD. For **a-g**, n=6. *****p<0.05; ******p<0.01; *******p<0.001; ********p<0.0001; ns, not significant.

**Supplementary Figure S8. Effects of KLF5 on endothelial cell proliferation and tube formation regulated by nHDL and dHDL**


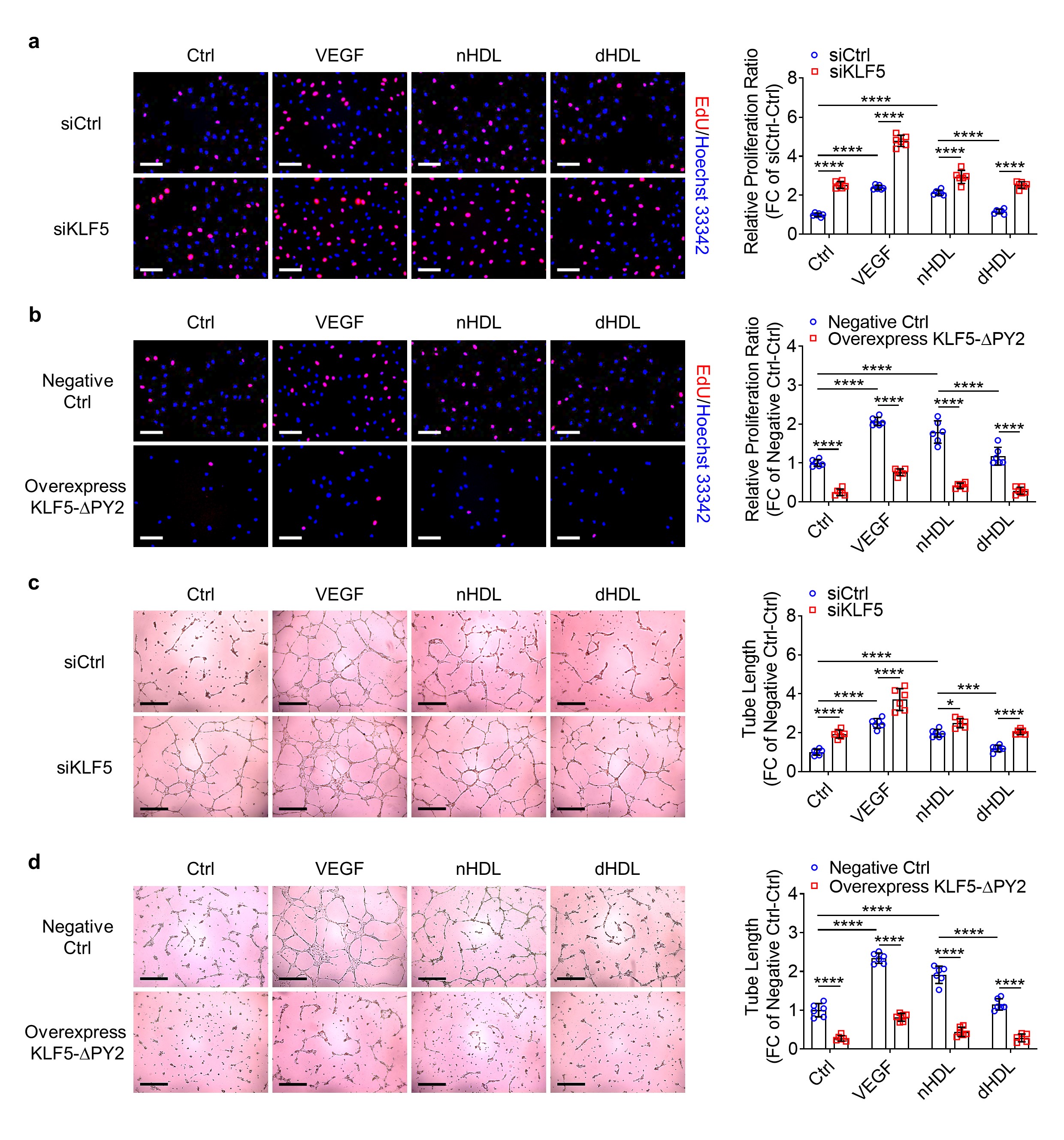


**a, b.** The representative images (left) and quantification (right) of 5-Ethynyl-2’-deoxyuridine (EdU) incorporation assay in human umbilical vein endothelial cells (HUVECs) treated with vascular endothelial growth factor (VEGF) or nHDL or dHDL after knocking down krueppel-like factor 5 (KLF5) (**a**) or overexpressing KLF5 of PY2 deletion (KLF5-ΔPY2) (**b**). The proliferative HUVECs were labeled with EdU (red) and the nuclei were stained with Hoechst 33342 (blue). Scale bars, 100 μm. **c, d.** The representative images (left) and quantification (right) of tube formation assay in HUVECs treated with VEGF or nHDL or dHDL after knocking down KLF5 (**c**) or overexpressing KLF5-ΔPY2 (**d**). Scale bars, 500 μm. Data are presented as the mean ± SD. For all experiments, n=6. *****p<0.05; *******p<0.001; ********p<0.0001.


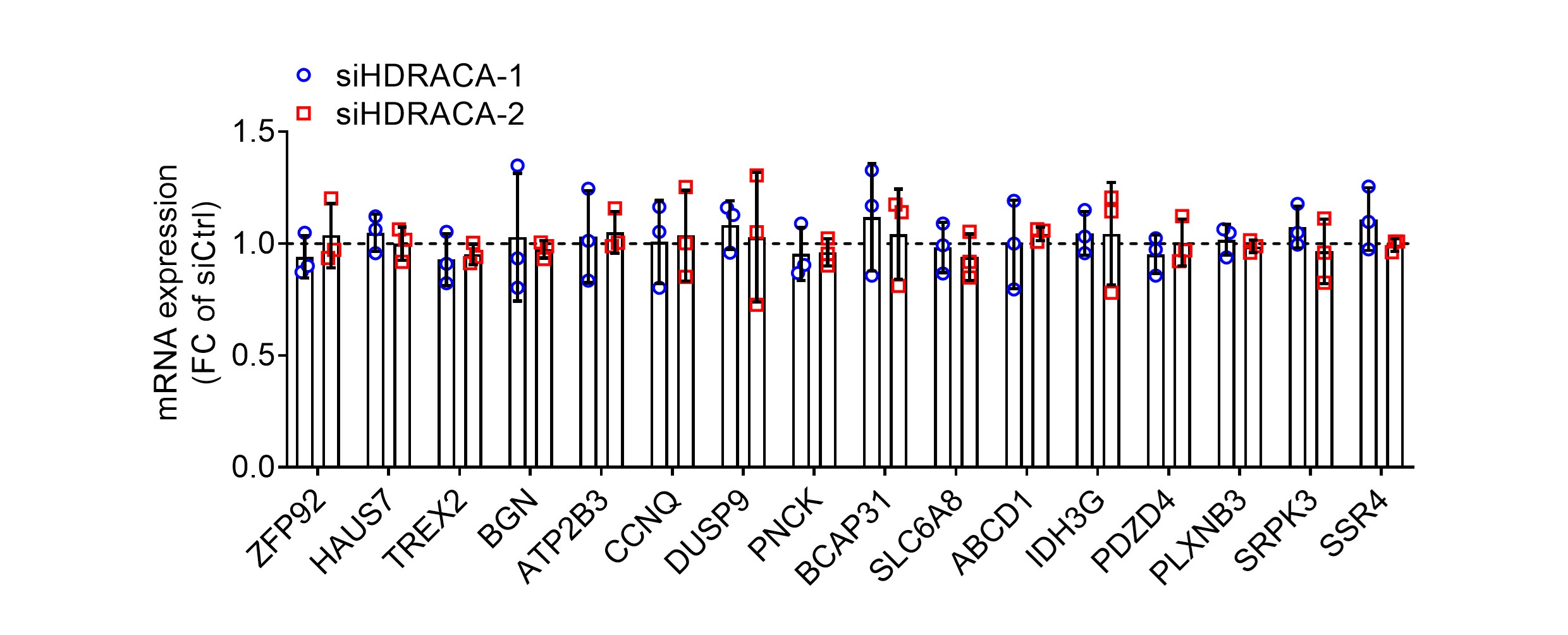


**Supplementary Figure S9. HDRACA doesn’t affect the expression of neighboring genes in the endothelial cells**

The expression of neighboring protein coding genes of HDRACA in HUVECs transfected with negative control siRNA or HDRACA-siRNAs were determined by RT-qPCR assays. siHDRACA-1 and siHDRACA-2 represent siRNAs targeting two different sites of HDRACA. Data was normalized to negative control siRNA group (siCtrl), and the dotted lines represent the values of the siCtrl group. Data are presented as the mean ± SD. n=3.

**Supplementary Figure S10. nHDL and dHDL differently regulate the expression of HDRACA to affect the interaction between RAIN and vigilin**


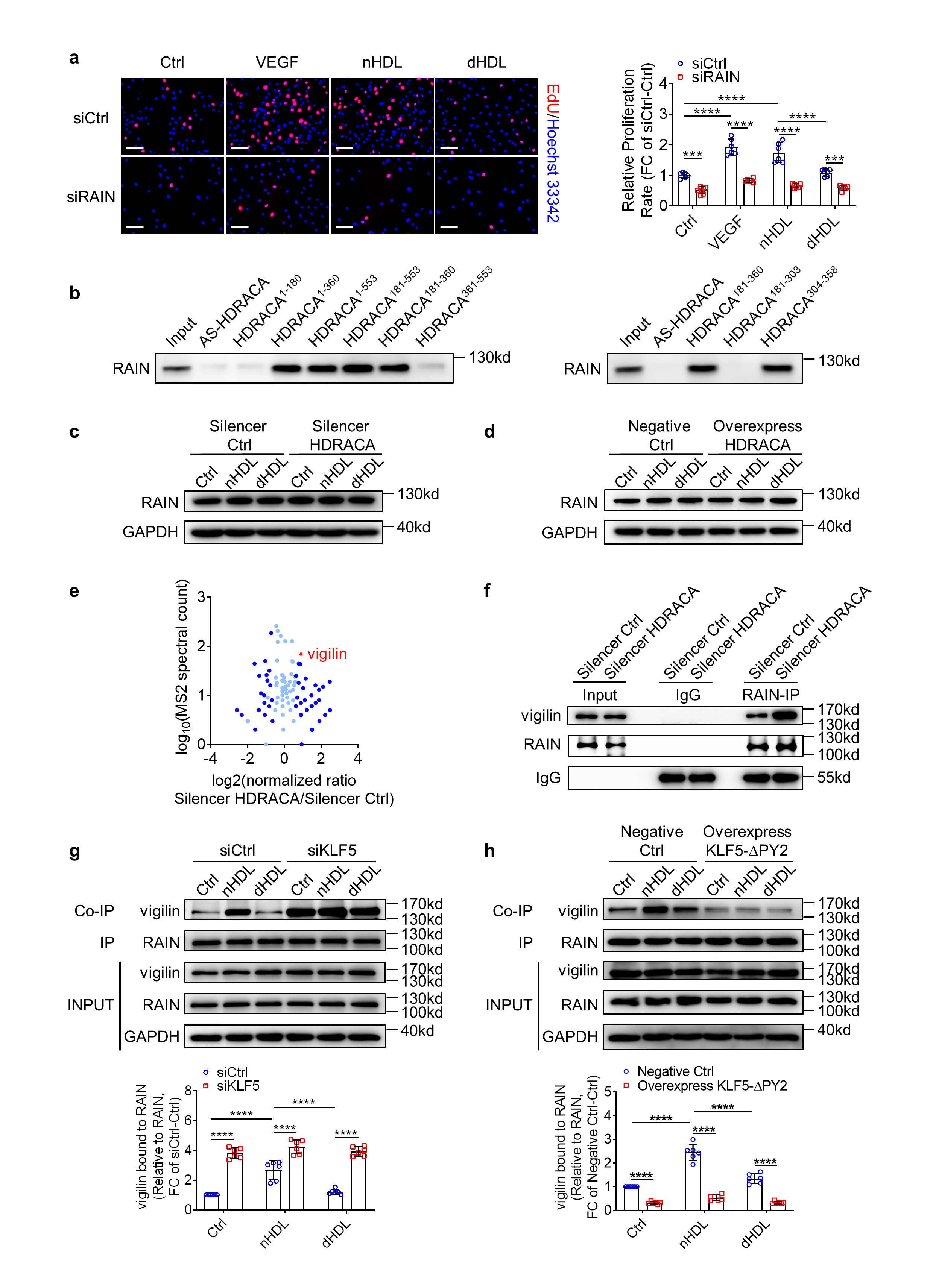


**a.** The representative images (left) and quantification (right) of 5-Ethynyl-2’-deoxyuridine (EdU) incorporation assay in human umbilical vein endothelial cells (HUVECs) treated with vascular endothelial growth factor (VEGF) or nHDL or dHDL after transfected with negative control siRNA or Ras-interacting protein 1 (RAIN)-siRNA (siRAIN). The proliferative HUVECs were labeled with EdU (red) and the nuclei were stained with Hoechst 33342 (blue). Scale bars, 100 μm. **b.** The interaction between HDRACA truncations and RAIN was determined by RNA pull down. **c, d.** Immunoblot of the level of RAIN in HUVECs treated with nHDL or dHDL after silencing (**c**) or overexpressing (**d**) HDRACA. **e.** Scatter plot of protein MS2 spectral count from HUVECs transfected with Negative Control Smart Silencer or HDRACA lncRNA Smart Silencer. Significantly different proteins were colored in dark blue (Fold Change>log_2_(1.5) or Fold Change<-log_2_(1.5) and unique peptide≥2). Other proteins are shown in light blue and vigilin is shown in red. **f.** Immunoprecipitation analysis of the interaction of RAIN and vigilin in HUVECs after silencing HDRACA. **g, h.** Immunoprecipitation analysis of the interaction of RAIN and Vigilin in HUVECs treated with nHDL or dHDL after knocking down KLF5 (**g**) or overexpressing KLF5-ΔPY2 (**h**). Representative plots (up) and quantification (down) are shown. Data are presented as the mean ± SD. For a-h, n=6. *******p<0.001; ********p<0.0001.

**Supplementary Figure S11. HDRACA competes with Vigilin to bind the Dilute domain of RAIN**


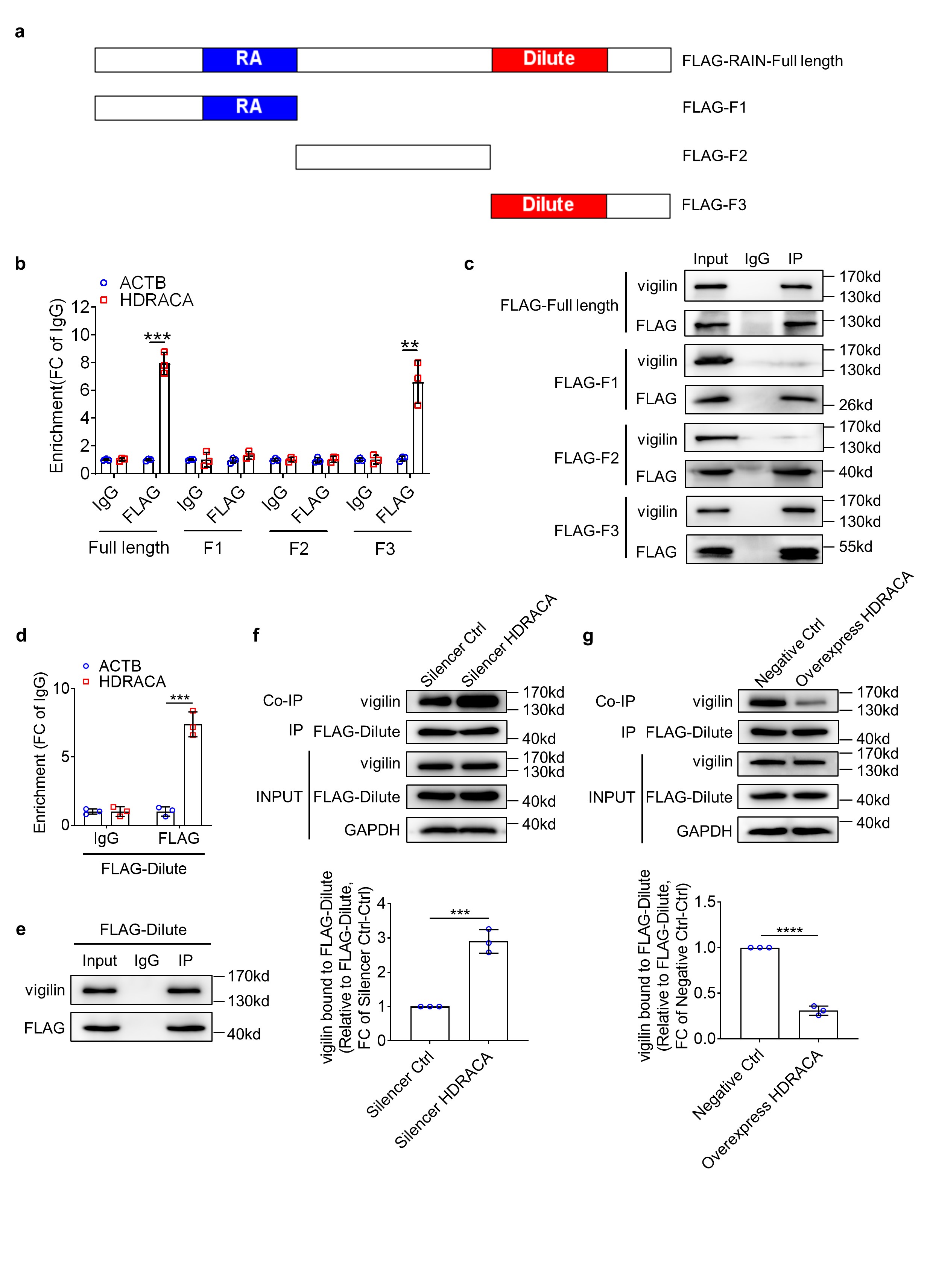


**a.** Schematic diagram of FLAG tagged Ras-interacting protein 1 (RAIN) truncated mutants. **b.** The interaction between HDRACA and FLAG tagged RAIN truncated mutants in human umbilical vein endothelial cells (HUVECs) were determined by RNA-immunoprecipitation (RIP) assays. **c.** The interaction between FLAG tagged RAIN truncated mutants and Vigilin in HUVECs were evaluated by Immunoprecipitation analysis. **d.** The interaction between HDRACA and FLAG tagged Dilute domain of RAIN in HUVECs were demonstrated by RIP assays. **e.** Immunoprecipitation analysis showed the interaction between FLAG tagged Dilute domain of RAIN and Vigilin in HUVECs. **f, g.** Immunoprecipitation analysis showed the interaction of FLAG tagged Dilute domain of RAIN and Vigilin in HUVECs after silencing (**f**) or overexpressing (**g**) HDRACA. Representative plots (up) and quantification (down) are shown. Data are presented as the mean ± SD. For **b-g**, n=3. ******p<0.01; *******p<0.001; ********p<0.0001.


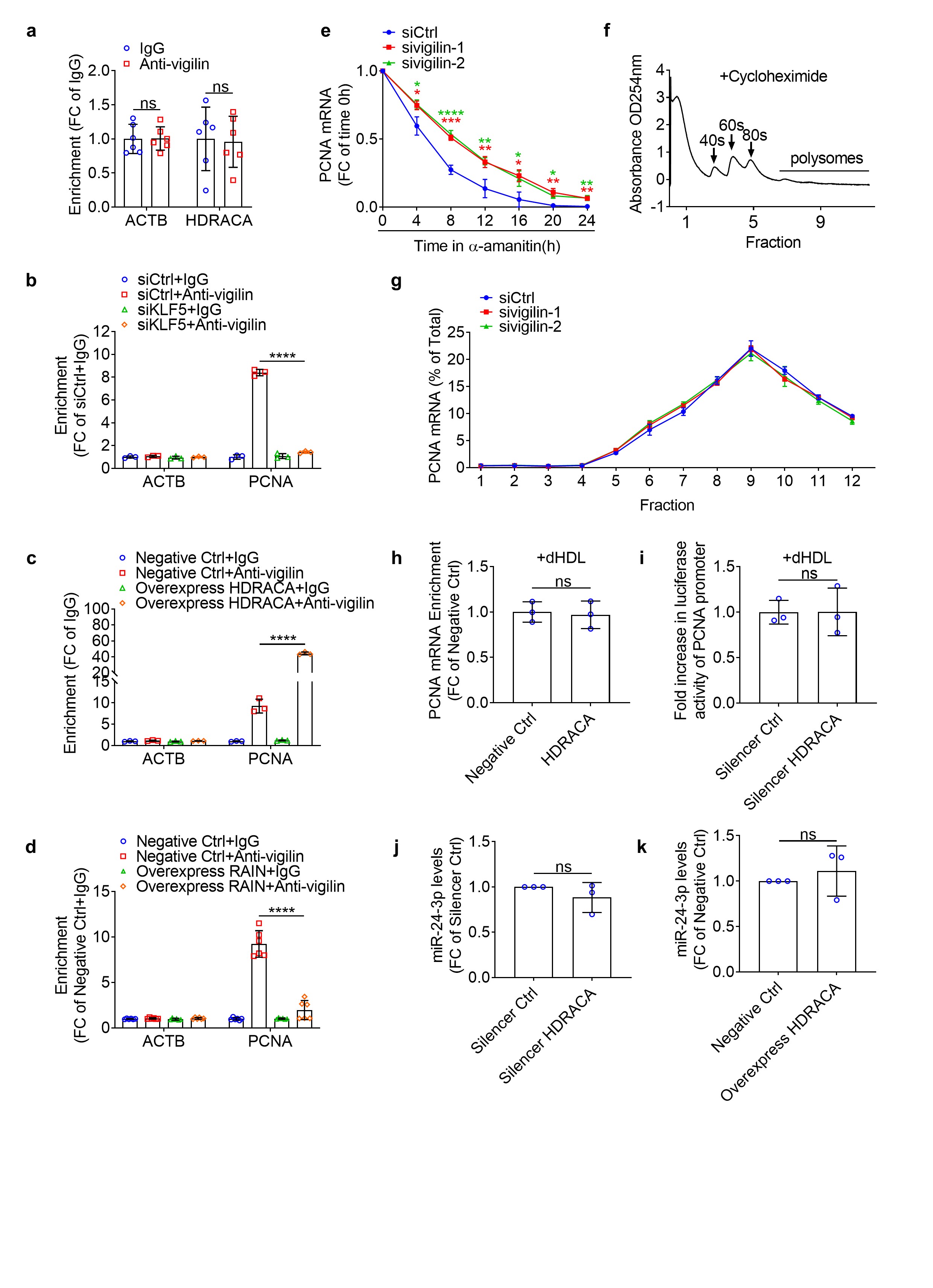


**Supplementary Figure S12. KLF5-HDRACA-RAIN signaling regulates the interaction between Vigilin protein and PCNA mRNA to affect the stability of PCNA mRNA**

**a.** The binding of Vigilin protein with HDRACA in human umbilical vein endothelial cells (HUVECs) was determined by RNA-immunoprecipitation (RIP) assay. **b-d.** RIP assays demonstrated the interaction between Vigilin protein and proliferating cell nuclear antigen (PCNA) mRNA in HUVECs after knocking down krueppel-like factor 5 (KLF5) (**b**), or overexpressing HDRACA (**c**) or overexpressing Ras-interacting protein 1 (RAIN) (**d**). **e.** RT-qPCR assays showed time-course of PCNA mRNA levels in HUVECs incubated with α-amanitin (10 μM) after transfected with negative control siRNA or Vigilin-siRNAs. **f, g.** Polysome gradient analysis of HUVECs transfected with negative control siRNA or Vigilin-siRNAs. (**f**) Global RNA polysome profiles generated by the density gradient fractionation system are shown. (**g**) The relative distribution of the PCNA mRNA over the sucrose gradient was assayed by RT-qPCR. **h.** RNA antisense purification (RAP) assays showed the interaction between HDRACA and PCNA mRNA in dHDL-treated HUVECs. **i.** Transcription activities of PCNA promoter after silencing HDRACA in dHDL-treated HUVECs were detected by luciferase reporter assays. **j.** The levels of miR-24-3p in HUVECs after silencing HDRACA were determined by RT-qPCR. **k.** The levels of miR-24-3p in HUVECs after overexpressing HDRACA were determined by RT-qPCR. Data are presented as the mean ± SD. For **a** and **d**, n=6. For **b**, **c**, **e-k**, n=3. For **a-d**, **h-k**, ********p<0.0001; ns, not significant. For **e**, *****p<0.05; ******p<0.01; *******p<0.001; ********p<0.0001 compared to negative control siRNA group (siCtrl). Red symbols represents siVigilin-1 versus siCtrl. Green symbols represent siVigilin-2 versus siCtrl.

**Supplementary Figure S13. HDRACA is successfully expressed in the hindlimbs of mice, without affecting RAIN and Vigilin levels in the endothelial cells**


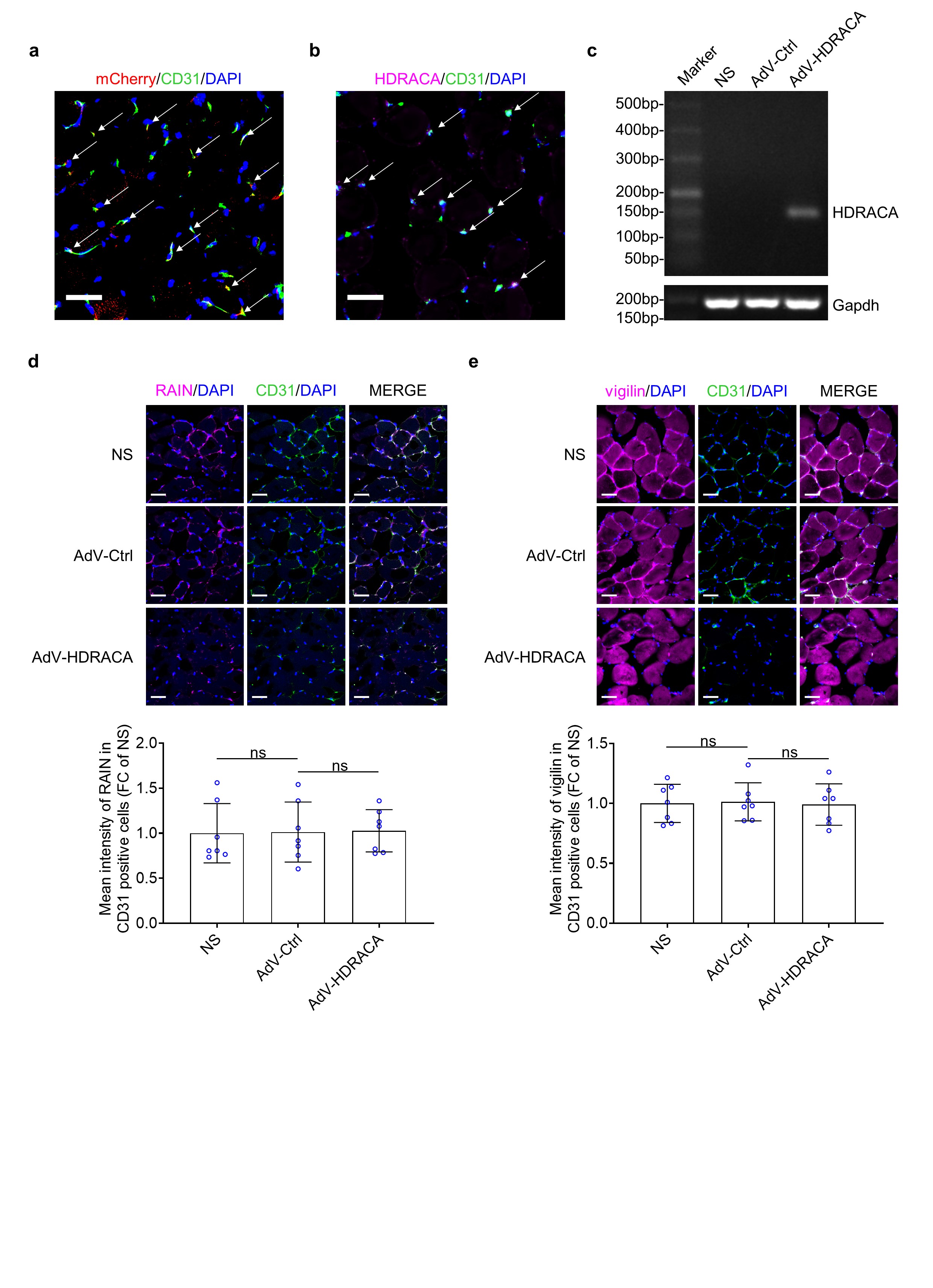


**a.** CD31 staining (green) in sections from adenovirus-injected limbs. mCherry fluorescence is shown (red). The white arrows indicated the cells expressing both mCherry and CD31. The nuclei were stained with 4',6-diamidino-2-phenylindole (DAPI) (blue). Scale bars, 50 μm. **b.** Fluorescence in situ hybridization (FISH)-immunofluorescence showed the colocalization of HDRACA (magenta) and CD31 (green) in the hindlimb adductor muscle at day 7 after adenoviral transfer. The white arrows indicated the cells expressing both HDRACA and CD31. The nuclei were stained with DAPI. Scale bars, 50 μm. **c.** Reverse transcription PCR (RT-PCR) showed the expression of human HDRACA in the hindlimb adductor muscle of C57BL/6 mice model at day 7 post-surgery. **d.** The representative images (up) and quantification (down) of CD31 (green) and RAIN (magenta) co-staining at day 14 after femoral artery ligation in C57BL/6 mice treated with normal saline (NS), control adenovirus vector (AdV-Ctrl) or adenovirus vector carrying HDRACA (AdV-HDRACA). The nuclei were stained with DAPI. Scale bars, 50 μm. **e.** The representative images (up) and quantification (down) of CD31 (green) and vigilin (magenta) co-staining at day 14 after femoral artery ligation in C57BL/6 mice treated with normal saline (NS), control adenovirus vector (AdV-Ctrl) or adenovirus vector carrying HDRACA (AdV-HDRACA). The nuclei were stained with DAPI. Scale bars, 50 μm. Data are presented as the mean ± SD. For all the experiments, n=7. ns, not significant.

**Supplementary Figure S14. Matrigel plug assays furtherly confirm the role of HDRACA in inhibiting angiogenesis.**


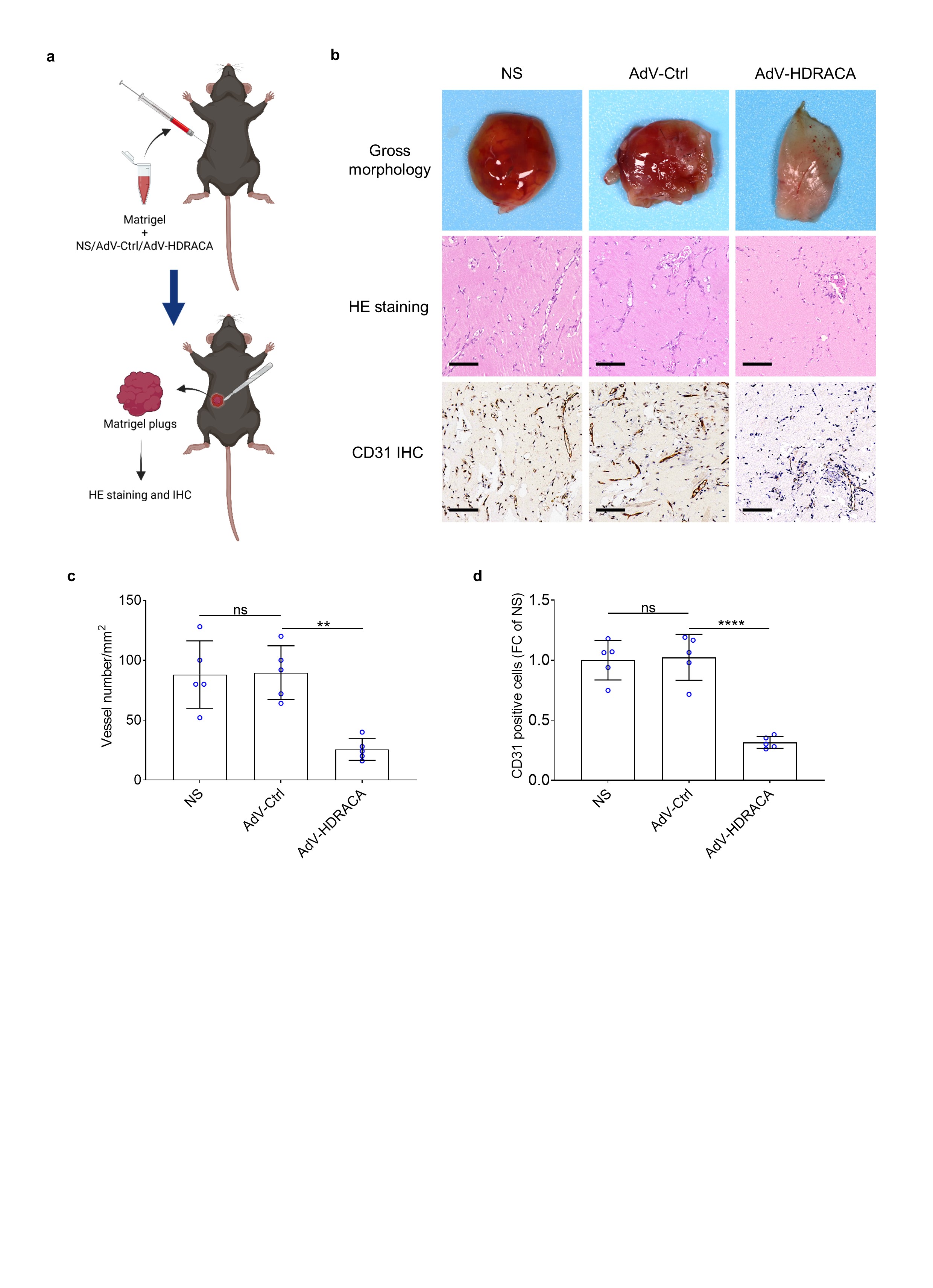


**a.** Schematic graphic of Matrigel plug assay. **b.** Gross morphology, Hematoxylin-eosin (HE) staining and CD31 immunohistochemistry (IHC) of Matrigel plug assays. Scale bars, 100 μm. **c.** Number of vessels per mm^2^ quantified from HE staining. **d.** Quantification of CD31 positive cells in CD31 immunohistochemistry. NS, normal saline; AdV-Ctrl, control adenovirus vector; AdV-HDRACA, adenovirus vector carrying HDRACA. Data are presented as the mean ± SD. For all the experiments, n=5. ******p<0.01; ********p<0.0001.


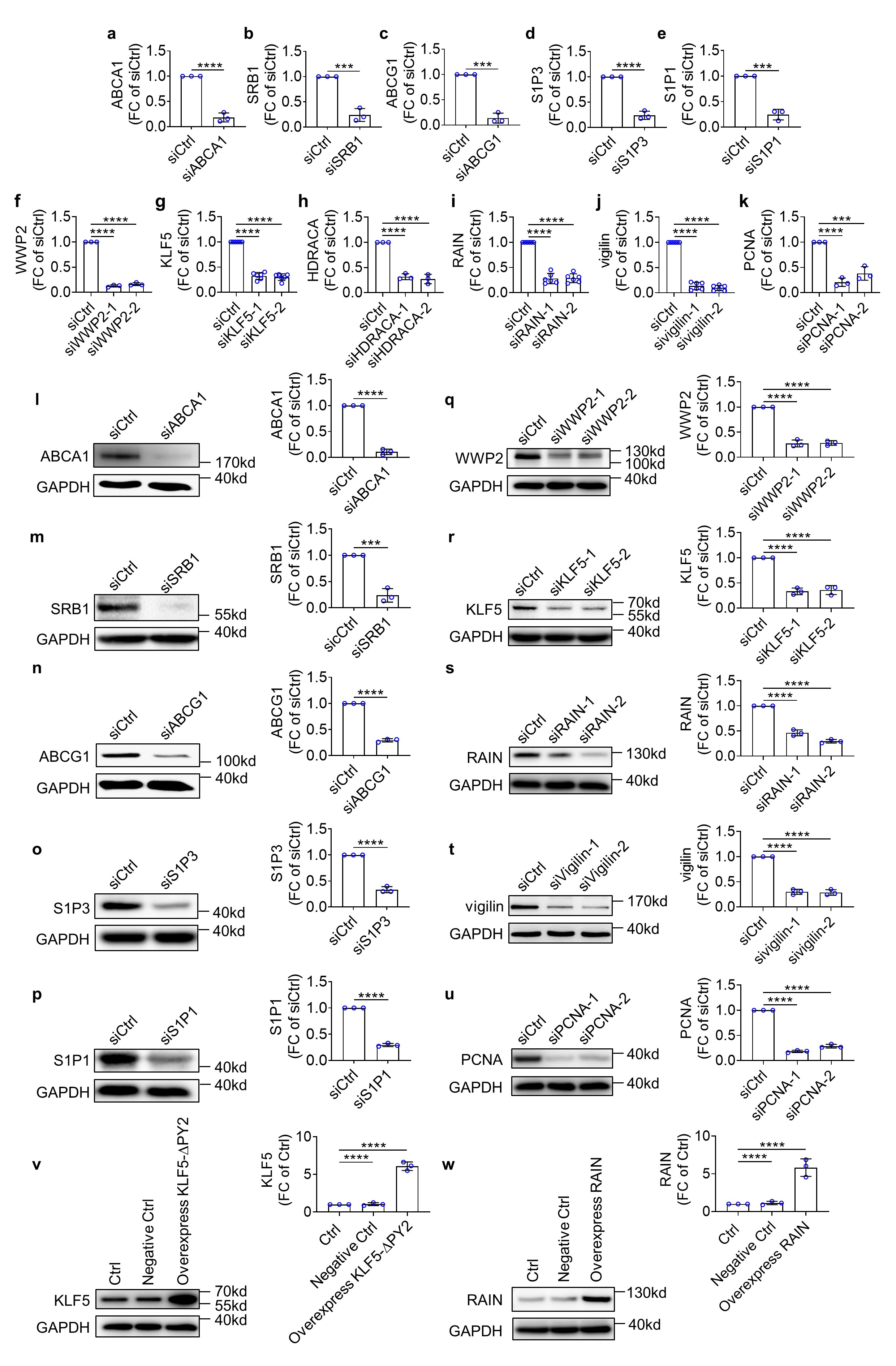


**Supplementary Figure S15. Verification of knockdown and overexpression**

**a-e.** The mRNA levels of HDL receptors of ATP-binding cassette transporter A1 (ABCA1) (**a**), scavenger receptor class B type 1 (SRB1) (**b**), ATP-binding cassette transporter G1 (ABCG1) (**c**), sphingosine-1-phosphate receptor 3 (S1P3) (**d**) and sphingosine-1-phosphate receptor 1 (S1P1) (**e**) in human umbilical vein endothelial cells (HUVECs) transfected with negative control siRNA or their siRNAs were determined by RT-qPCR. **f-k.** The mRNA levels of WW domain-containing E3 ubiquitin protein ligase 2 (WWP2) (**f**), krueppel-like factor 5 (KLF5) (**g**), HDRACA (**h**), Ras-interacting protein 1 (RAIN) (**i**), vigilin (**j**) and proliferating cell nuclear antigen (PCNA) (**k**) were determined by RT-qPCR. **l-p.** Immunoblotting for the expression of HDL receptors ABCA1 (**l**), SRB1 (**m**), ABCG1 (**n**), S1P3 (**o**) and S1P1 (**p**) in HUVECs transfected with negative control siRNA or their siRNAs. The representative plots (left) and quantification (right) are shown. **q-u.** Immunoblotting for WWP2 (**q**), KLF5 (**r**), RAIN (**s**), Vigilin (**t**) and PCNA (**u**) in HUVECs transfected with negative control siRNA or their siRNAs. The representative plots (left) and quantification (right) are shown. **v.** Immunoblotting for KLF5 in HUVECs transfected with negative control lentiviruses or KLF5-ΔPY2-overexpressing lentiviruses. The representative plots (left) and quantification (right) are shown. **w.** Immunoblotting for RAIN in HUVECs transfected with negative control lentiviruses or RAIN-overexpressing lentiviruses. The representative plots (left) and quantification (right) are shown. Data are presented as the mean ± SD. For **a-f**, **h**, **k**, **l-w**, n=3. For **g**, **i** and **j**, n=6. *******p<0.001; ********p<0.0001.

**Supplementary Table S1. Demographic and clinical data of subjects for isolating HDL or detecting S1P，HDL protein and HDRACA levels in plasma.**

|  | **CAD (n=115)** | **Controls (n=100)** | **P-value** |
| --- | --- | --- | --- |
| Male[no.(%)] | 61(53.04) | 42(42.00) | 0.11 |
| Age[years] | 54.78±0.66 | 52.88±1.05 | 0.17 |
| Smoking[no.(%)] | 37(32.17) | 22(22.00) | 0.10 |
| Body mass index[kg/m^2^] | 25.56±0.39 | 23.36±0.40 | <0.05 |
| HBA1c [%] | 5.49±0.04 | 5.50±0.04 | 0.22 |
| Heart rate[bpm] | 72.70±1.16 | 74.22±1.36 | 0.96 |
| Mean arterial pressure[mmHg] | 91.08±1.15 | 87.77±1.05 | <0.05 |
| Total cholesterol[mmol/L] | 4.56±0.12 | 4.32±0.08 | <0.05 |
| Triglycerides[mmol/L] | 1.90±0.09 | 1.15±0.05 | <0.05 |
| HDL cholesterol[mmol/L] | 0.97±0.02 | 1.19±0.03 | <0.05 |
| LDL cholesterol[mmol/L] | 2.92±0.09 | 2.66±0.05 | <0.05 |
| Creatinine[mmol/L] | 74.03±1.65 | 68.68±1.51 | 0.10 |

CAD: coronary artery disease;

HBA1c: Hemoglobin A1c;

HDL: high-density lipoprotein;

LDL: low-density lipoprotein.

**Supplemental Table S2. Demographic and clinical data of subjects for patients with ASO and control subjects.**

|  | **ASO(n=10)** | **Controls(n=10)** | **P-value** |
| --- | --- | --- | --- |
| Male[no.(%)] | 7(70%) | 6(60%) | 1 |
| Age[years] | 60.20±1.81 | 54.10±2.38 | 0.06 |
| Smoking[no.(%)] | 5(50%) | 0(0%) | <0.05 |
| Heart rate[bpm] | 85.00±4.55 | 86.20±5.36 | 0.87 |
| Mean arterial pressure[mmHg] | 93.67±4.47 | 99.77±5.03 | 0.38 |
| Diabetes mellitus[no.(%)] | 7(70%) | 0(0%) |  |
| statins[no.(%)] | 6(60%) | 0(0%) |  |

ASO: arteriosclerosis obliterans;

HDL: high-density lipoprotein;

LDL: low-density lipoprotein.

**Supplemental Table S3. HDRACA RNA-binding proteins identified by RNA-binding protein pull-down assays and mass spectrometry analysis.** Proteins specifically bound to HDRACA with emPAI≥0.1 and score>100 were listed.

| **Protein name** | **Score** | **Matches** | **Sequences** | **emPAI** |
| --- | --- | --- | --- | --- |
| Tubulin beta-3 chain | 833 | 23 | 10 | 1.13 |
| Tubulin beta-2B chain | 711 | 20 | 9 | 1.01 |
| Tubulin beta-4A chain | 633 | 19 | 10 | 1.02 |
| Tubulin alpha-4A chain | 462 | 16 | 10 | 1.58 |
| Heat shock protein beta-1 | 271 | 7 | 4 | 0.98 |
| Y-box-binding protein 3 | 250 | 5 | 5 | 0.49 |
| Protein ITPRID2 | 250 | 5 | 5 | 0.12 |
| Reticulocalbin-2 | 229 | 3 | 3 | 0.29 |
| Propionyl-CoA carboxylase alpha chain, mitochondrial | 197 | 5 | 5 | 0.22 |
| Nestin | 186 | 6 | 6 | 0.12 |
| 40S ribosomal protein S17 | 184 | 4 | 3 | 0.8 |
| Myosin regulatory light polypeptide 9 | 177 | 5 | 4 | 0.87 |
| ATP-dependent RNA helicase A | 176 | 4 | 4 | 0.1 |
| Actin-related protein 3 | 160 | 6 | 3 | 0.4 |
| Pyruvate carboxylase, mitochondrial | 160 | 4 | 4 | 0.1 |
| EH domain-containing protein 2 | 158 | 4 | 4 | 0.23 |
| Keratin, type II cytoskeletal 6B | 148 | 4 | 3 | 0.17 |
| Ras-interacting protein 1 | 145 | 4 | 4 | 0.13 |
| Calcium-binding mitochondrial carrier protein Aralar2 | 143 | 3 | 3 | 0.14 |
| Protein-glutamine gamma-glutamyltransferase 2 | 142 | 5 | 4 | 0.18 |
| Serine/arginine-rich splicing factor 1 | 137 | 4 | 3 | 0.4 |
| T-complex protein 1 subunit gamma | 136 | 2 | 2 | 0.11 |
| 40S ribosomal protein SA | 134 | 1 | 1 | 0.1 |
| Tropomodulin-3 | 122 | 2 | 2 | 0.17 |
| Coatomer subunit epsilon | 117 | 3 | 2 | 0.31 |
| T-complex protein 1 subunit theta | 115 | 2 | 2 | 0.11 |
| Serpin H1 | 112 | 2 | 2 | 0.15 |
| Dolichyl-diphosphooligosaccharide--protein glycosyltransferase subunit 2 | 111 | 2 | 2 | 0.1 |
| Cofilin-1 | 109 | 2 | 2 | 0.39 |
| ATPase family AAA domain-containing protein 3A | 108 | 4 | 3 | 0.14 |
| Serine hydroxymethyltransferase, mitochondrial | 102 | 3 | 3 | 0.19 |

**Supplemental Table S4. Proteins binding to RAIN that were reduced after transfection with lncRNA Smart Silencer targeting HDRACA compared with Negative Control Smart silencer.** Proteins with log_2_(normalized ratio Silencer HDRACA/Silencer Ctrl)<- log_2_(1.5) and unique peptides ≥2 were listed.

| **Symbol** | **Unique.peptides** | **MS2 spectral counts** | **iBAQ** | **log_2_(FC)** |
| --- | --- | --- | --- | --- |
| KRT9 | 33 | 186 | 201920000 | -0.725184706 |
| KRT5 | 2 | 50 | 22878000 | -1.045373986 |
| DSP | 34 | 44 | 681790 | -1.626410336 |
| KPRP | 14 | 32 | 4044000 | -0.816692787 |
| JUP | 10 | 26 | 1842500 | -0.969556329 |
| DSG1 | 18 | 25 | 1526900 | -1.365968243 |
| FLG2 | 10 | 20 | 930390 | -0.925395652 |
| HEL113 | 12 | 16 | 1047000 | -0.767609318 |
| HRNR | 9 | 11 | 381460 | -1.270499856 |
| DSC1 | 6 | 10 | 944210 | -1.100574855 |
| RPS6 | 5 | 8 | 5757700 | -0.685537355 |
| PRRC2A | 6 | 8 | 120670 | -0.685537355 |
| RPLP2 | 5 | 7 | 3153800 | -1.089047314 |
| HEL-S-2a | 3 | 6 | 1253600 | -0.826012909 |
| KRT77 | 3 | 6 | 367880 | -0.826012909 |
| ALB | 5 | 6 | 236090 | -0.826012909 |
| HBB | 4 | 5 | 1472700 | -2.584962501 |
| TGM3 | 2 | 5 | 219330 | -1.388193028 |
| SERPINB12 | 3 | 4 | 2185100 | -2.321928095 |
| ARG1 | 3 | 4 | 858930 | -1.066264933 |
| C1QBP | 3 | 4 | 827350 | -1.066264933 |
| CAT | 3 | 4 | 720670 | -1.066264933 |
| hCG_1811539 | 3 | 4 | 557980 | -1.066264933 |
| HEL-S-102 | 3 | 4 | 434580 | -1.066264933 |
| IGHG1 | 3 | 4 | 270940 | -1.066264933 |
| PIP | 3 | 4 | 88358 | -1.066264933 |
| HBA1 | 2 | 3 | 2675300 | -2 |
| PKP1 | 3 | 3 | 1233300 | -2 |
| CDC26 | 2 | 3 | 876110 | -0.651227433 |
| CSRP1 | 2 | 3 | 845250 | -0.651227433 |
| HNRPCL1 | 2 | 3 | 731030 | -0.651227433 |
| KRT80 | 2 | 3 | 613680 | -0.651227433 |
| RPN1 | 2 | 3 | 401180 | -0.651227433 |
| RPS18 | 2 | 3 | 339900 | -0.651227433 |
| RPS7 | 2 | 3 | 260900 | -0.651227433 |
| XP32 | 2 | 3 | 185130 | -0.651227433 |
| AZGP1 | 3 | 3 | 101430 | -0.651227433 |
| KCTD5 | 3 | 3 | 84663 | -0.651227433 |
| PABPC1 | 3 | 3 | 59292 | -0.651227433 |
| ECM1 | 2 | 2 | 2248500 | -1.584962501 |
| FUS | 2 | 2 | 234480 | -1.584962501 |
| LOR | 2 | 2 | 225740 | -1.584962501 |
| RPS17 | 2 | 2 | 123990 | -1.584962501 |
| SFPQ | 2 | 2 | 55906 | -1.584962501 |
| TGM1 | 2 | 2 | 45425 | -1.584962501 |

Symbol: Protein gene symbol.

Unique.peptides: The total number of unique peptides associated with the protein group (i.e. these peptides are not shared with another protein group).

MS2 spectral counts: MS2 spectra number map to this protein group.

iBAQ: Intensity-based absolute quantification (iBAQ) is an approximation of protein copy numbers based on the sum of peptide-feature intensities of all peptides matching to a protein divided by the number of theoretically observable peptides.

FC: ratio of the normalized MS2 spectral counts of Silencer HDRACA/Silencer Ctrl.

**Supplemental Table S5. Proteins binding to RAIN that were increased after transfection with lncRNA Smart Silencer targeting HDRACA compared with Negative Control Smart Silencer.** Proteins with log_2_(normalized ratio Silencer HDRACA/Silencer Ctrl)>log_2_(1.5) and unique peptides ≥2 were listed.

| **Symbol** | **Unique.peptides** | **MS2 spectral counts** | **iBAQ** | **log_2_(FC)** |
| --- | --- | --- | --- | --- |
| HDLBP | 38 | 71 | 4906000 | 0.896928052 |
| CKAP4 | 21 | 44 | 4980500 | 0.775905818 |
| LMNA | 25 | 43 | 4195900 | 0.869015222 |
| TUBA1B | 2 | 25 | 4788700 | 0.638161055 |
| HEL-S-72p | 13 | 25 | 2091800 | 0.638161055 |
| NCL | 12 | 22 | 4978300 | 1.152734228 |
| RRBP1 | 18 | 19 | 257430 | 2.457126355 |
| VWF | 13 | 18 | 181130 | 0.768269714 |
| TUBB | 3 | 16 | 2362600 | 1.681763599 |
| FLNB | 15 | 15 | 199340 | 2.096801098 |
| RPL8 | 7 | 14 | 10797000 | 1.014662445 |
| GOLPH2 | 9 | 14 | 1316900 | 1.014662445 |
| FLNC | 11 | 13 | 117340 | 1.876090005 |
| DKFZp686F1345 | 8 | 10 | 119280 | 1.464968001 |
| RPS25 | 4 | 9 | 7082400 | 0.691822301 |
| RPL7 | 5 | 8 | 3907800 | 1.1068598 |
| OSBPL3 | 7 | 8 | 294240 | 1.88195927 |
| RPS2 | 2 | 7 | 2856700 | 0.887804754 |
| MX1 | 5 | 7 | 218730 | 1.691822301 |
| RPL17 | 3 | 6 | 3807700 | 0.629429743 |
| DDX3X | 5 | 6 | 509570 | 0.629429743 |
| FHL2 | 5 | 6 | 252020 | 0.629429743 |
| MVP | 5 | 6 | 161290 | 1.472767255 |
| MAP4 | 6 | 6 | 129470 | 1.472767255 |
| HNRNPA1 | 4 | 5 | 552430 | 1.214392243 |
| ILF3 | 4 | 5 | 465940 | 1.214392243 |
| ATP5B | 3 | 5 | 205760 | 2.472767255 |
| RPS20 | 2 | 4 | 9593400 | 0.899425145 |
| LRRC59 | 3 | 4 | 3889600 | 0.899425145 |
| RPS13 | 3 | 4 | 2008400 | 0.899425145 |
| EHD4 | 4 | 4 | 762850 | 0.899425145 |
| XRCC6 | 4 | 4 | 746140 | 0.899425145 |
| RPL27A | 3 | 4 | 127700 | 2.214392243 |
| SDPR | 4 | 4 | 50467 | 2.214392243 |
| SYNCRIP | 3 | 3 | 132190 | 1.899425145 |
| AFP | 2 | 2 | 2450100 | 1.495915186 |
| ATG9A | 2 | 2 | 1022300 | 1.495915186 |
| CEP97 | 2 | 2 | 357400 | 1.495915186 |
| DKFZp686J01190 | 2 | 2 | 279880 | 1.495915186 |
| EPB41L3 | 2 | 2 | 176120 | 1.495915186 |
| FXR1 | 2 | 2 | 147870 | 1.495915186 |
| GNB2L1 | 2 | 2 | 136150 | 1.495915186 |
| IFI16 | 2 | 2 | 123440 | 1.495915186 |
| MRE11A | 2 | 2 | 107380 | 1.495915186 |
| RAD50 | 2 | 2 | 102080 | 1.495915186 |
| RPL10 | 2 | 2 | 67030 | 1.495915186 |
| RPL23 | 2 | 2 | 58746 | 1.495915186 |
| SAMHD1 | 2 | 2 | 47426 | 1.495915186 |
| SERPINH1 | 2 | 2 | 35477 | 1.495915186 |
| TRA1 | 2 | 2 | 33487 | 1.495915186 |
| EIF2S3 | 4 | 2 | 15597 | 1.495915186 |
| MAGT1 | 2 | 1 | 921640 | 0.933735067 |
| SPATS2L | 2 | 1 | 115370 | 0.933735067 |
| TUBB2C | 2 | 1 | 675400 | 0.933735067 |

Symbol: Protein gene symbol.

Unique.peptides: The total number of unique peptides associated with the protein group (i.e. these peptides are not shared with another protein group).

MS2 spectral counts: MS2 spectra number map to this protein group.

iBAQ: Intensity-based absolute quantification (iBAQ) is an approximation of protein copy numbers based on the sum of peptide-feature intensities of all peptides matching to a protein divided by the number of theoretically observable peptides.

FC: ratio of the normalized MS2 spectral counts of Silencer HDRACA/Silencer Ctrl.

**Supplementary Data**

**Supplementary table S6.** The sequences for siRNAs, ASONs, lncRNA Smart Silencers, primers and probe used in this study

**Supplementary table S7.** Differentially expressed genes in the RNA-seq analysis of HUVECs transfected with Negative Control Smart Silencer or HDRACA lncRNA Smart Silencer
